# Topological Fermi arc interference on low-symmetry Weyl surfaces

**DOI:** 10.1038/s41467-026-76639-5

**Published:** 2026-08-13

**Authors:** Cong Li, Zhilong Yang, Hongxiong Liu, Magnus H. Berntsen, Francesco Scali, Dibya Phuyal, Jianfeng Zhang, Timur K. Kim, Jacek Osiecki, Balasubramanian Thiagarajan, Youguo Shi, Tao Xiang, Quansheng Wu, Oscar Tjernberg

**Affiliations:** 1 Beijing National Laboratory for Condensed Matter Physics, Institute of Physics, Chinese Academy of Sciences, Beijing, China; 2 Department of Applied Physics, KTH Royal Institute of Technology, Stockholm, Sweden; 3 School of Mathematics and Physics, University of Science and Technology Beijing, Beijing, China; 4 Dipartimento di Fisica, Politecnico di Milano, Milano, Italy; 5 Diamond Light Source, Harwell Campus, Didcot, UK; 6 MAX IV Laboratory, Lund University, Lund, Sweden

**Keywords:** Electronic properties and materials, Topological matter

## Abstract

Topological materials are defined by the correspondence between bulk topology and boundary states, yet this correspondence becomes enigmatic on low-symmetry surfaces where bulk and surface periodicities may not coincide within a conventional first bulk Brillouin zone projection. Here we study the (103) surface of the Weyl semimetal NdAlSi and identify Fermi arc interference in the boundary spectrum. Angle-resolved photoemission spectroscopy uncovers loop-like Fermi-arc connectivity and characteristic replica modulations that are not observed on high symmetry surfaces. Crucially, the topological surface states themselves are reconstructed because Fermi arcs from phase-shifted bulk-zone projections overlap and hybridize, producing connectivity patterns unique to low-symmetry facets. We show that these emerge from incomplete bulk projection and multi-cell interference governed by a least-common-multiple framework. Least-common-multiple guided density functional theory and Green’s-function calculations reproduce the reconstructed periodicity and dominant replica structure in the spectra, providing a broadly applicable commensuration guideline. These findings resolve the apparent bulk-boundary correspondence paradox on low-symmetry surfaces and provide an operational route to model and interpret boundary spectra on complex facets.

## Abstract (web summary)

Bulk-boundary correspondence in Weyl semimetals is difficult to interpret on low-symmetry facets. Here, the authors resolve this issue on the NdAlSi(103) surface, showing that phase-shifted bulk-zone projections hybridize Fermi arcs into reconstructed, replica-rich surface spectra.

## Introduction

Since the discovery of topological materials, a central question in condensed-matter physics has been how bulk topology manifests at material boundaries. Nowhere is this connection more striking than in Weyl semimetals^1–11^, where open Fermi arcs on high-symmetry surfaces such as (001)/(100) of tetragonal or orthorhombic lattices^5–7,12–14^ and the (0001) facet of hexagonal lattices^15,16^ directly connect the surface projections of bulk Weyl points. These canonical surfaces, where bulk and surface periodicities are trivially commensurate, have accordingly dominated both experimental and theoretical studies of topological semimetals.

In contrast, low-symmetry surfaces remain largely unexplored^17–19^, despite offering a qualitatively new regime of topology. The nontrivial relation between the projected bulk Brillouin zone (BZ) and the surface BZ (SBZ) on low-symmetry facets can complicate the conventional one-to-one mapping between bulk Weyl points and surface Fermi arcs, raising the question of how bulk-boundary correspondence (BBC) should be formulated when the surface spectrum requires multi-zone projection and folding. Whether BBC in this regime survives in a modified form or requires a new organizing principle remains unknown, as no direct experimental observation or coherent theoretical framework has yet addressed it. This gap stems from challenges on both fronts: experimentally, low-symmetry facets rarely cleave into atomically uniform planes^19^, and even when achieved, disorder and multiple terminations obscure intrinsic surface states in angle-resolved photoemission spectroscopy (ARPES) measurements; theoretically, using an undersized surface cell or restricting bulk-to-surface mapping to the first bulk BZ can miss the correct surface repeat unit and the corresponding folding/replica structure, complicating quantitative comparisons between density functional theory (DFT) and experiment. Because Weyl semimetals themselves originate from broken symmetries, low-symmetry surfaces naturally extend this principle into the boundary, where reduced crystalline constraints are expected to generate qualitatively new boundary phenomena–ranging from anisotropic or reconstructed Fermi arcs^13,20,21^ to nonlinear transport^22–25^ and higher-order boundary states^26^. A compact framework that identifies the minimal commensurate surface repeat unit and connects it to experimentally observable spectral reconstruction is therefore essential for advancing this frontier.

Here, we overcome these barriers by realizing a well-defined (103) surface of the Weyl semimetal NdAlSi through precise pre-orientation and pre-cutting of single crystals, followed by controlled cleavage that exposes atomically uniform terraces. This preparation enables high-resolution ARPES measurements that reproducibly resolve multiple surface-state configurations and reveal a reconstructed periodicity accompanied by replica features. We show that this reconstruction follows from treating bulk-to-surface mapping as a multi-BZ projection problem. Successive bulk BZ projections appear on the surface as laterally phase-shifted replicas and close only after a least-common-multiple (LCM) commensuration cycle, which we demonstrate, both mathematically and through direct experimental consistency, to be equivalent to the standard intercept-based surface-cell construction. We further emphasize that a complete three-dimensional to two-dimensional bulk-to-surface projection is the general starting principle. In many common cases, the LCM condition gives ${N}_{\min }=1$, so the result reduces to an effective first-BZ picture. LCM-consistent surface calculations capture the reconstructed periodicity and dominant replica structure observed in ARPES, while finer aspects of the arc connectivity, including loop-like features, depend on microscopic boundary conditions such as termination mixing, reconstruction/disorder, and enhanced hybridization. This establishes an operational route to reconcile low-symmetry surface spectra with bulk topology and realistic boundary conditions.

## Results

**Fig. 1:**
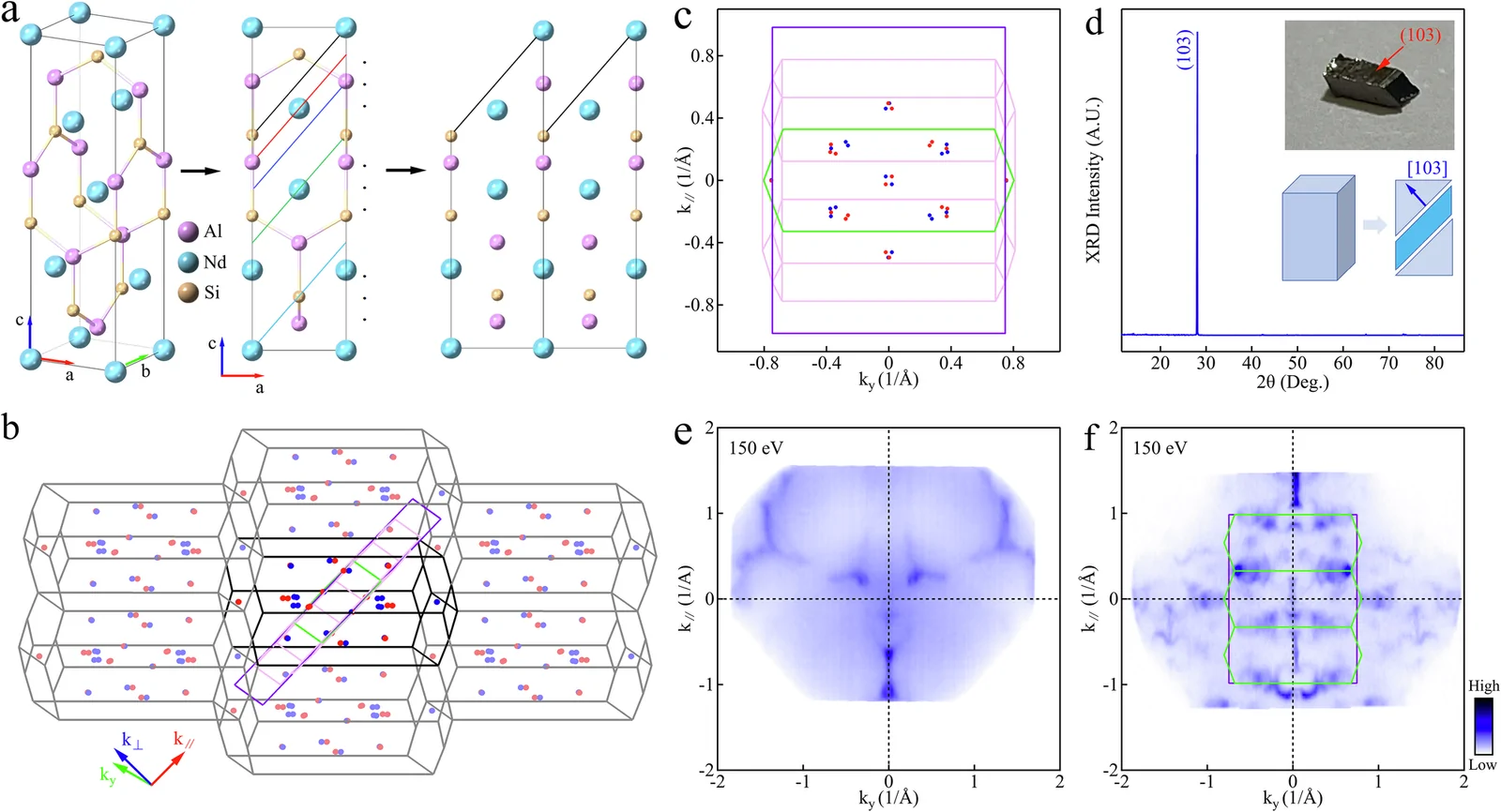
Crystal structure and electronic projections of NdAlSi (103) surface. **a** Crystal structure of NdAlSi with the (103) surface cut. The differently colored cuts represent the potential cleavage planes. The rightmost panel shows one possible cleavage plane. **b** BZ construction and projection onto the (103) surface. **c** Periodicity mismatch between the SBZ and the first bulk BZ projection. The red (blue) dots corresponding to Weyl points with chirality +1 (−1). **d** XRD confirming the exposed (103) surface. **e**, **f** Fermi surfaces measured at 150 eV on uneven (disordered) (**e**) and flat (well defined) (**f**) regions of the (103) surface. Purple lines denote the (103) theoretical SBZ, whose size is determined by the (103) cross section of half a primitive unit cell. Since the body-centered lattice halves the real-space periodicity along the [001] direction, the corresponding BZ along *k*_*z*_ is doubled. For consistency, the (103) SBZ is likewise defined by the cross section of half a primitive unit cell (see Supplementary Information Section 4 for the details). Green lines denote the experimentally observed periodicity extracted from (**f**); pink lines denote the first bulk BZ projected onto the (103) surface.

We begin with the Weyl semimetal NdAlSi^12–14,27^, a non-centrosymmetric member of the RAlSi (R = rare-earth) family^28–31^, which hosts well-characterized Weyl points and high-quality single crystals ideally suited for surface-sensitive spectroscopy. We focus on the low-symmetry (103) surface, which has not been systematically explored by ARPES, yet provides a natural setting to probe how BBC manifests when bulk and surface periodicities are incompatible. The crystallographic structure and orientation of the (103) surface are shown in Fig. 1a, while Fig. 1b illustrates the bulk BZ and its projection. Unlike high-symmetry facets such as (001) or (100), the (103) projection exhibits an intrinsic mismatch between the periodicities of the SBZ (purple lines) and the projected bulk BZ (pink lines), as shown in Fig. 1c–this incommensurability forms the experimental starting point of our study.

The nontrivial bulk-to-surface projection geometry motivates direct experimental verification on an atomically well-defined (103) surface. The surface orientation was confirmed by X-ray diffraction (XRD), which verifies the high-quality exposure of the (103) plane (Fig. 1d; see “Methods” for sample preparation). Fermi-surface maps acquired at 150 eV further highlight the crucial role of surface quality: uneven regions (Fig. 1e) show diffuse features dominated by bulk states, whereas well-defined terraces (Fig. 1f) exhibit sharp surface states that follow the periodicity of the SBZ (purple lines). A closer inspection, however, reveals an additional modulation with a smaller period (green lines), indicating a more subtle reconstruction. On the uneven region, the suppression of coherent surface states^14^ exposes bulk-like bands whose periodicity does not coincide with that of the SBZ. These observations demonstrate that the mismatch between bulk and surface periodicities is not merely a geometric construct but a directly observable phenomenon on the (103) surface. This raises a central question: if Weyl points follow the bulk BZ periodicity while surface Fermi arcs adhere to the SBZ, does the BBC break down on low-symmetry surfaces?

**Fig. 2:**
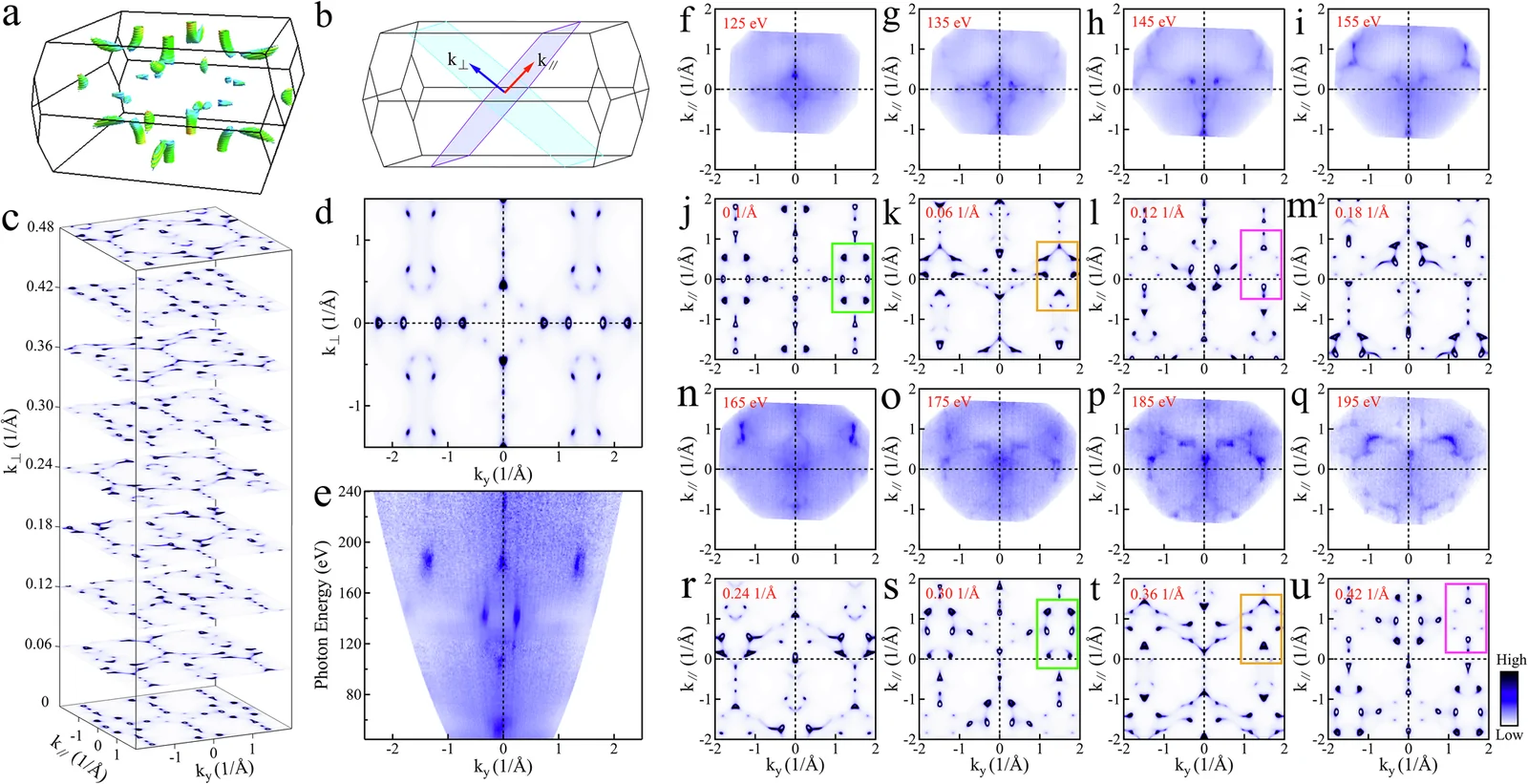
Bulk electronic structures of the (103) surface. **a** DFT calculated 3D Fermi surface of NdAlSi. **b** The (103) surface and its perpendicular cross-section in momentum space are expected to be probed with photon energies ranging from approximately 125 to 205 eV. **c** DFT calculated bulk Fermi surface of the momentum-space slices along the [103] direction (*k*_⊥_). **d** DFT-calculated bulk Fermi surface of the plane [cyan plane in (**b**)] perpendicular to the (103) surface [purple plane in (**b**)]. **e** Photon-energy dependent ARPES spectral intensity map at the Fermi level along the *k*_*y*_ direction on a relatively strong disordered (103) surface, where both surface states and SBPSs are nearly suppressed. **f**–**i**, **n**–**q** Photon energy dependent bulk Fermi surface mappings (125–195 eV) revealing *k*_⊥_ dispersion. **j**–**m**, **r**–**u** Corresponding DFT calculations. Each slice produces a shifted replica of the bulk Fermi surface within the SBZ, reflecting the intrinsic periodicity of bulk states along the [103] direction; the green, orange, and pink boxes highlight features that are shifted as *k*_⊥_ changes. The features do not appear strictly identical here because only discrete *k*-slices are shown, but denser sampling would reveal exact repetitions.

To understand the nature of the electronic states on the (103) surface and answer the above question, we performed systematic ARPES measurements combined with bulk DFT calculations. The 3D bulk Fermi surface from DFT is shown in Fig. 2a, while Fig. 2b illustrates the (103) surface orientation and its perpendicular cross-section in momentum space. Calculated bulk Fermi surface slices along the [103] direction (k_⊥_) and perpendicular to the (103) surface (cyan plane in Fig. 2b) are presented in Fig. 2c, d, serving as theoretical benchmarks.

Experimentally, the photon energy dependent ARPES spectral intensity map at the Fermi level along k_*y*_ (Fig. 2e) reveals strong k_⊥_ modulation, a hallmark of bulk-derived states. Systematic Fermi surface measurements over photon energies from 125 to 195 eV (Fig. 2f–i, n–q) further confirm this k_⊥_ dispersion. The corresponding DFT calculations (Fig. 2j–m, r–u) reproduce the experimental features with remarkable accuracy. More detailed bulk Fermi surfaces of the NdAlSi (103) surface are provided in Supplementary Information Section 3.

Beyond establishing the bulk band structure, these results provide critical insight into the apparent breakdown of the BBC. In particular, the evolution of bulk Fermi surface slices along [103] reveals a systematic in-plane translation of features as *k*_⊥_ is varied. Each slice produces a shifted replica of the bulk Fermi surface within the SBZ, reflecting the intrinsic periodicity of bulk states along the [103] direction. As successive bulk BZs are considered, these replicas accumulate into an effective superlattice periodicity that is distinct from the primitive bulk BZ but naturally commensurate with the SBZ.

From this perspective, the apparent mismatch between the periodicity of projected Weyl points and that of the surface Fermi arcs is a direct consequence of restricting the projection to the first bulk BZ. The bulk projections along [103] demonstrate experimentally that multiple BZs must be included: only then does the accumulated superlattice restore the periodicity needed to match the surface states. This insight provides the experimental foundation for resolving the BBC paradox, as developed below.

**Fig. 3:**
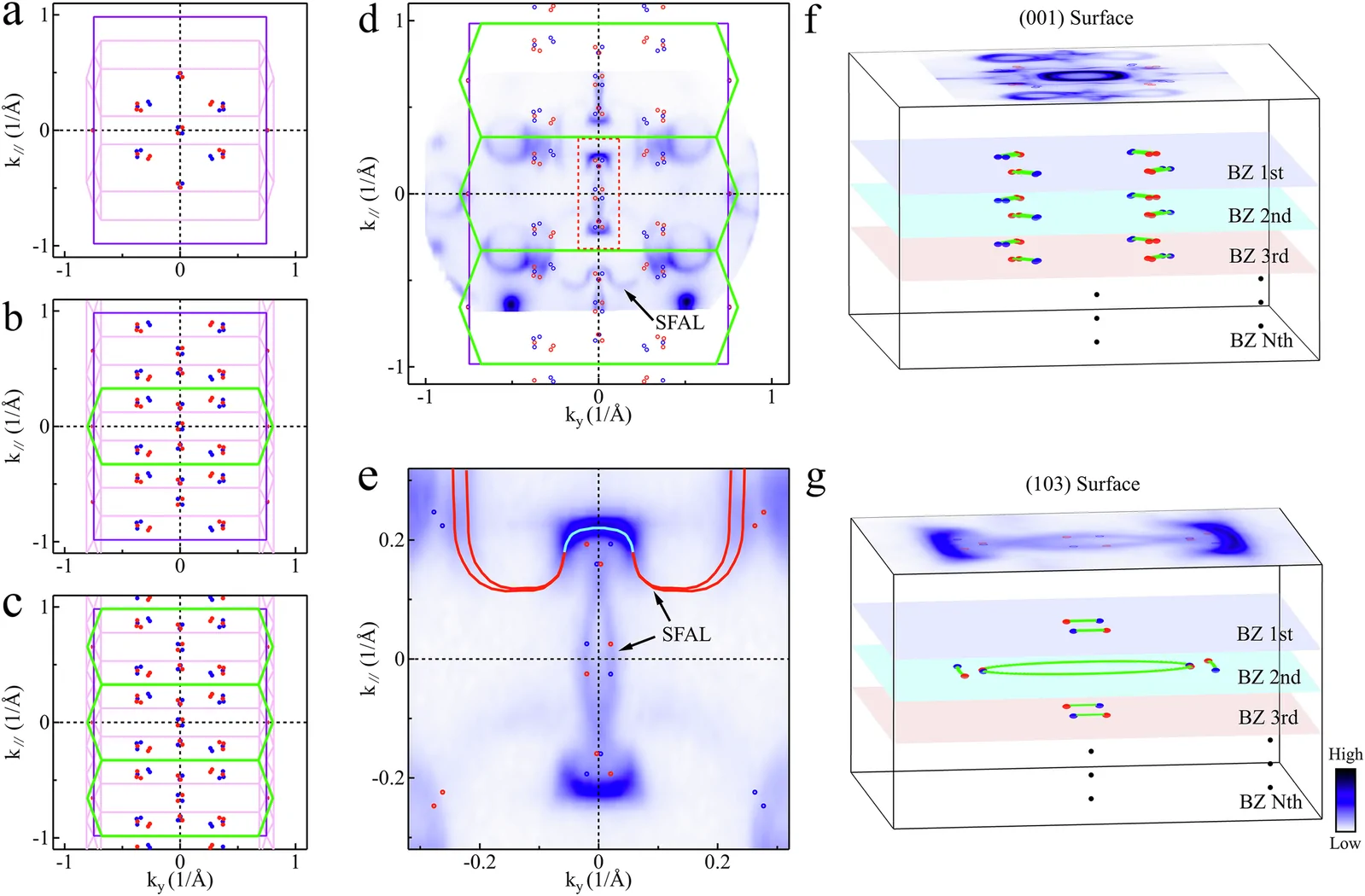
Resolving the BBC paradox on the (103) surface. **a** Projection of Weyl points onto the (103) surface considering only the first bulk BZ, showing a mismatch with the surface periodicity. **b**, **c** Inclusion of the second-order (**b**) and third-order (**c**) bulk BZs along *k*_*z*_ progressively restores the periodicity and brings the bulk projection into agreement with the surface states. Red (blue) dots denote Weyl points of chirality +1 (−1). **d**, **e** Comparison between the bulk projection Weyl points and the measured surface states demonstrates that multiple BZs are required to recover the correct periodicity. **e** is the magnified view of (**d**). The red and cyan lines represent surface Fermi arcs, which hybridize to form SFALs as indicated by the black arrows. **f**, **g** General scheme for different surfaces: for the (001) surface (**f**), the first bulk BZ is sufficient, whereas for the (103) surface (**g**), more bulk BZ projections are need to generate a superlattice periodicity consistent with the SBZ. The Fermi surface in (**g**) is an enlarged view of the red dashed boxed region in (**d**). Purple lines denote the (103) theoretical SBZ; green lines denote the newly formed (103) SBZ, corresponding to the SBZ observed in experiment; pink lines denote the first bulk BZ projected onto the (103) surface. The connection of the surface Fermi arcs in (**g**) is not the actual connectivity, but rather a schematic illustration indicating that Fermi arcs from different bulk BZ may hybridize upon projection onto the (103) surface (see Supplementary Information Section 6 for details).

To clarify how this resolves the apparent BBC paradox, we track how the bulk-projection periodicity evolves as higher-order BZs are included. If only the first bulk BZ is considered, the projected Weyl points (Fig. 3a) clearly mismatch the theoretical SBZ (purple lines) and the experimental SBZ (green lines). Adding the second-order (Fig. 3b) and third-order (Fig. 3c) BZs and Weyl points along *k*_*z*_ progressively restores the periodicity, bringing the bulk projection closer to the surface states. This restoration follows a LCM criterion: the projected displacement between successive bulk BZs closes only after an integer multiple of the in-plane reciprocal vectors. For the (103) surface of NdAlSi, the minimum closure occurs after ${N}_{\min }=3$ steps (see Supplementary Information Section 4). As illustrated in Fig. 3d, the projected Weyl points recover the theoretical SBZ periodicity (purple lines) once higher-order BZs are included.

Beyond this restoration, the higher–order projections reveal an emergent smaller repeating unit: a “new SBZ” (green lines in Fig. 3d) whose period along *k*_∥_ is one-third of the theoretical SBZ. This “new SBZ” coincides with the one observed experimentally. Equivalently, this effect can be viewed as a fixed lateral shift between successive bulk projections (see Supplementary Information Section 4), which manifests as a momentum-space Moiré-like pattern, a weak superlattice modulation with a period one-third of the original SBZ (Fig. 3d). In general, whether a single bulk BZ suffices depends on surface symmetry. For high-symmetry surfaces such as (001) of NdAlSi, the first bulk BZ already coincides with the SBZ (Fig. 3f). For low-symmetry surfaces such as (103), however, successive bulk projections are laterally shifted (Fig. 3g), and only after ${N}_{\min }$ steps does the bulk periodicity close. This comparison clarifies why a first BZ projection often appears sufficient on high-symmetry facets, whereas a complete 3D to 2D projection becomes essential whenever successive bulk-zone projections do not register, as also evidenced in known cases where more than one bulk BZ is required to account for surface-state phenomenology^32^. In a Weyl semimetal, this has an additional topological consequence: the phase-shifted multi-zone projections displace the surface Fermi arc manifolds so that they overlap and hybridize, thereby reconstructing the boundary connectivity beyond a simple folded SBZ picture. Exact spectral reconstruction is achieved only in the infinite-projection limit (see Supplementary Information Section 4). Thus, the observed mismatch is not a breakdown of the BBC but rather the natural consequence of incomplete bulk projection, while the emergent new SBZ reflects the Moiré-like interference among multiple shifted projections. Here we display one representative surface-state configuration; additional terminations observed on the same cleaved surface are presented in Supplementary Information Section 5.

This reconstruction has direct consequences for the surface Fermi arcs. On high-symmetry surfaces such as (001) of NdAlSi, arcs from successive bulk BZs project in perfect registry, typically preserving the canonical picture of open arcs directly connecting projected Weyl points (Fig. 3f). In sharp contrast, on low-symmetry surfaces like (103), the lateral mismatch between successive projections causes arcs associated with different bulk zones (displaced both in-plane and out-of-plane; Fig. 3g) to overlap and reconstruct through hybridization, so that the observed features are not simple replicas but collective superpositions. In certain cases, this reconstruction yields closed surface Fermi-arc loops (SFALs) within the reduced periodicity shown in Fig. 3d (see Supplementary Information Section 6 for details), a boundary connectivity unusual on high-symmetry facets.

Our framework, together with the best ARPES resolution presently achievable, provides compelling evidence for the emergence of SFALs. These loop-like states enrich the boundary phenomenology of Weyl semimetals: their closed *k*-space trajectories provide natural channels for quantum interference and oscillatory responses, while the concentrated Berry curvature they enclose may enhance nonlinear or nonreciprocal transport. Moreover, overlapping arc segments can promote nesting instabilities, offering a route to correlated boundary orders. Future high-resolution probes, such as spin-resolved ARPES or quantum oscillations, will provide complementary confirmation and deeper insight into the microscopic structure of these loops.

**Fig. 4:**
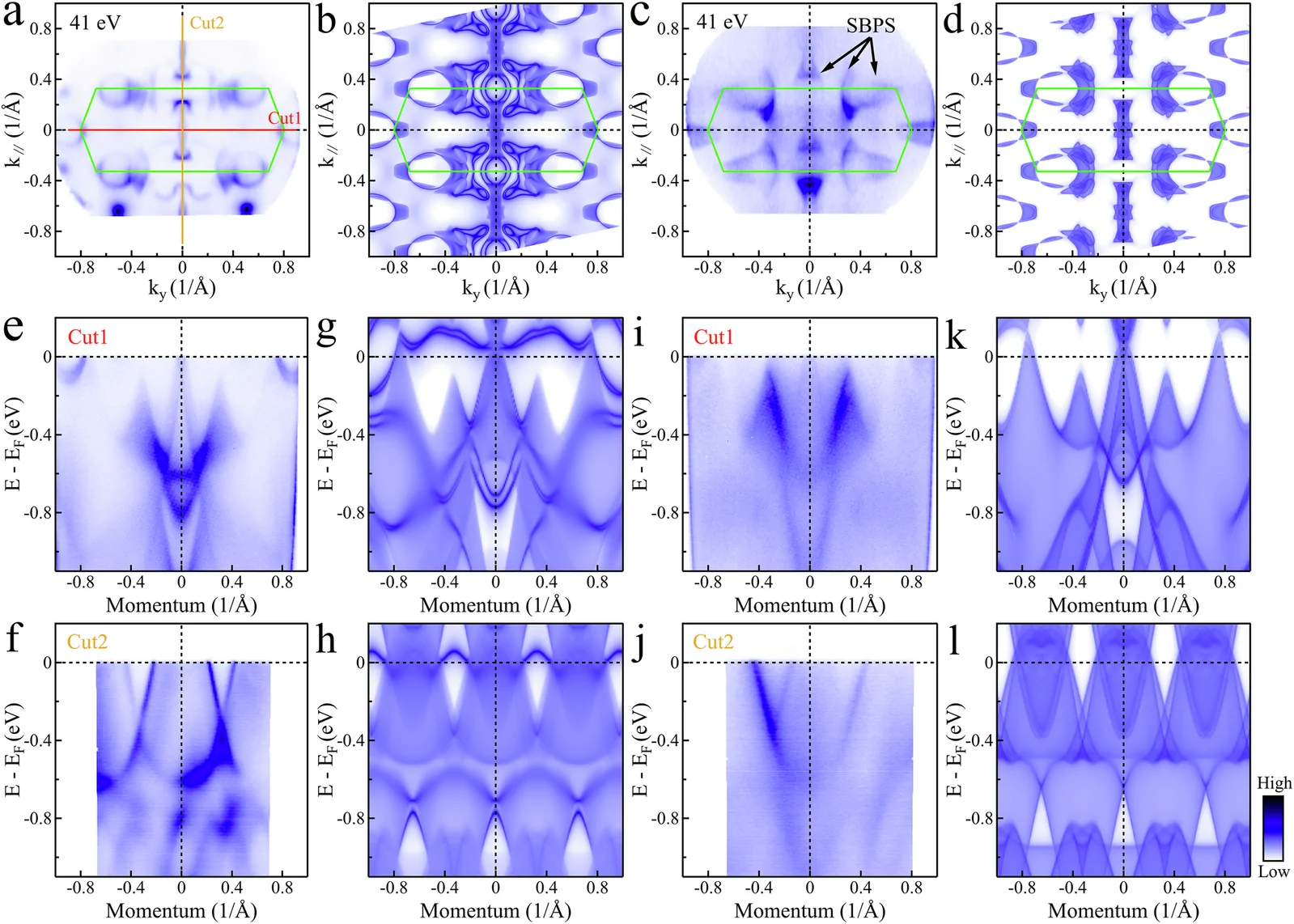
Comparison of experimental and DFT results for surface states and SBPSs on NdAlSi (103) surface. **a** Fermi surface of the NdAlSi (103) surface measured on a flat region with the photon energy of 41 eV, revealing surface states. **b** Surface projected DFT calculation of the (103) Fermi surface for a Si atom terminated surface cleavage at the Si-Al layer (see Supplementary Information Section 5 for details). **c** Impurity-induced disorder filters out surface states^14^, exposing SBPS. **d** DFT calculation of the corresponding SBPSs on the (103) surface. Green lines indicate the experimental SBZ, consistent with Fig. 1e. **e**, **f** Band dispersions measured along momentum Cut1 [red line in (**a**), (**e**)] and Cut2 [orange line in (**a**), (**f**)]. **g**, **h** Surface projected DFT band structures for the Si-terminated (103) surface along momentum Cut1 (**g**) and Cut2 (**h**). **i**, **j** Band dispersions along the same cuts measured on the disordered surface, where comparison with calculations (**k**, **l**) confirms the assignment of surface states and SBPSs.

To capture such arc reconstruction and hybridization theoretically, the surface periodicity used in modeling must be commensurate with bulk projections. This requirement is fulfilled by the LCM criterion, which provides a systematic prescription for selecting appropriate surface unit cells in DFT slab calculations to ensure commensurability between bulk projections and surface periodicities. In the case of the (103) surface of NdAlSi, the minimal commensuration requires ${N}_{\min }=3$, corresponding to a three-cell surface supercell composed of three consecutive unit cells. By contrast, a single-cell slab (or restricting to the first bulk BZ) systematically fails to reproduce the experimental periodicity, because the projection closure remains incomplete. With the correct three-cell supercell, surface-projected DFT captures the reduced periodicity and dominant features of the ARPES spectra. For a Si-terminated (103) surface (Fig. 4b), the LCM-consistent calculation reproduces the emergent “new smaller SBZ”, originating from the phase offsets of 2*π*/3 between bulk BZs of three consecutive, nonequivalent (103) surface unit cells. Their hybridization yields the reduced periodicity observed in experiment.

Despite this overall agreement, finer aspects of the Fermi-arc connectivity are not fully reproduced in the idealized calculations. In ARPES, loop-like surface connectivity SFALs is observed within the reduced SBZ, whereas the present LCM-consistent slab calculation captures the reconstructed periodicity and the dominant replica structure but yields predominantly open-arc connectivity. This difference indicates that the LCM framework resolves the bulk-surface commensuration (periodicity) required for a consistent BBC on a low-symmetry facet, while the detailed arc-to-loop connectivity is additionally controlled by microscopic boundary conditions (e.g., termination mixing, surface reconstruction/disorder, and enhanced hybridization among laterally shifted bulk-projection replicas) that are not uniquely fixed in an idealized slab model. We therefore regard the observed SFAL as an emergent property of low-symmetry surfaces beyond what is captured by ideal slab calculations. In addition to the Si-terminated regions, ARPES measurements also reveal two further surface-state configurations on other areas of the same cleaved (103) surface (see Supplementary Information Fig. 10). These distinct spectra share the reduced periodicity but do not match any of the terminations tested in Supplementary Information Fig. 11, suggesting a more complex origin, such as zigzag-type reconstructions or mixed local terminations beyond the simple three-cell picture in Supplementary Information Fig. 9b. A detailed discussion of these additional states, and their relation to the LCM criterion, is provided in Supplementary Information Section 5.

Beyond the ordered regions, disorder offers a complementary perspective on the same framework. When spectra are collected on a disordered area of the (103) surface, sharp surface states are strongly suppressed (Fig. 4c)^14^, effectively acting as a natural “filter” that allows otherwise hidden surface bulk-projected states (SBPSs) to emerge. Remarkably, these SBPSs are well reproduced by the same surface-projected DFT calculations (Fig. 4b), underscoring the robustness of the LCM-guided modeling. When a surface state enters the bulk-projected region, it hybridizes with the bulk continuum and evolves into a surface bulk-resonant state (SBRS)^33–40^, producing hybridized features observable in ARPES. Momentum-resolved band dispersions along two representative cuts (Cut1 and Cut2) are shown in Fig. 4e, f. These sharp dispersive bands agree well with surface-projected DFT calculations (Fig. 4g, h). By contrast, along the same cuts on the disordered surface (Fig. 4i, j), broader and bulk-like bands appear, consistent with calculated SBPSs (Fig. 4k, l). Supplementary Information Section 8 provide more details on the identification of surface states, bulk states, SBPSs, and SBRSs.

## Discussion

By investigating the low-symmetry (103) surface of the Weyl semimetal NdAlSi, we establish an experimentally grounded picture of how boundary spectra are reconstructed when bulk-to-surface mapping requires multi-BZ projections. While Weyl points follow the intrinsic bulk periodicity, the surface spectrum can exhibit a reconstructed periodicity within the SBZ, accompanied by replica features. What appears as a mismatch under a first-BZ projection is resolved once successive bulk BZs are included: laterally phase-shifted projections close after an LCM commensuration cycle, yielding the emergent reduced periodicity observed in ARPES. Beyond restoring commensuration, the essential physics here is that fractional projection shifts on a low-symmetry Weyl facet reconstruct the topological boundary states themselves: displaced surface Fermi arcs interact and reorganize their connectivity, producing loop-like and replica-modulated surface spectra.

Within this LCM-consistent description, surface calculations capture the reconstructed periodicity and the dominant replica structure, providing a consistent basis for comparing theory and experiment on a low-symmetry facet. At the same time, the detailed arc connectivity, including loop-like features observed in ARPES, depends sensitively on microscopic boundary conditions such as termination mixing, reconstruction/disorder, and enhanced surface-bulk hybridization, which are not uniquely fixed in an idealized slab model. This separation clarifies which aspects are dictated by commensuration/folding and which encode realistic boundary complexity.

More broadly, the LCM criterion provides a strict and operational guideline for selecting the minimal commensurate surface repeat unit required for accurate surface modeling and for interpreting ARPES on low-symmetry facets. The same procedure can be applied to other topological semimetals and more complex lattice metrics, where commensuration may require larger surface cells or, in the incommensurate limit, progressively higher-order projections. From a practical standpoint, it offers a robust route to determine the minimal surface supercell needed for reliable DFT slab or surface Green’s-function calculations on low-symmetry surfaces.

In summary, our work resolves an apparent inconsistency of BBC on a low-symmetry Weyl surface by establishing an operational framework for boundary spectral reconstruction based on multi-BZ projection and LCM commensuration. We show that projection-induced phase offsets can generate Moiré-like replica modulations and reshape surface Fermi arc connectivity in ways not captured by the conventional first-zone picture. These results highlight low-symmetry facets as a controllable setting where commensuration and microscopic boundary conditions can be used to tailor topological boundary spectra, providing a practical route for exploring reconstructed surface states in complex crystalline and potentially quasiperiodic geometries.

## Methods

### Sample growth

Single crystals of NdAlSi were grown from Al as flux. Nd, Al, Si elements were sealed in an alumina crucible with the molar ratio of 1:10:1. The crucible was finally sealed in a highly evacuated quartz tube. The tube was heated up to 1273 K, maintained for 12 h and then cooled down to 973 K at a rate of 3 K per hour. Single crystals were separated from the flux by centrifuging. The Al flux attached to the single crystals were removed by dilute NaOH solution.

### Sample preparation of the (103) surface

To obtain the low-symmetry (103) surface of NdAlSi, we first determined the crystallographic orientation of the single crystals by single crystal XRD. Based on the identified orientation, the crystals were cut with a high-precision diamond wire saw to expose the desired (103) direction. Prior to mounting, a shallow laser pre-cut was introduced along the target orientation, which facilitated controlled cleavage of the sample inside the ARPES chamber. This procedure enabled reliable preparation of well-defined (103) surfaces suitable for spectroscopic measurements.

### ARPES measurements

High-resolution ARPES measurements were performed at the I05 beamline of the Diamond synchrotron and at the Bloch beamline of the MAX IV synchrotron. The total energy resolution (analyzer and beamline) was set at 15–20 meV for the measurements. The angular resolution of the analyser was ~ 0.1°. The beamline spot size on the sample was about 50 × 50 μm at the I05 beamline of the Diamond synchrotron and about 10 × 12 μm at the Bloch beamline of the MAX IV synchrotron. The samples were cleaved in situ and measured in ultrahigh vacuum with a base pressure better than 1.0 × 10^−10^ mbar at about 8 K at the I05 beamline of the Diamond synchrotron and about 18 K at the Bloch beamline of the MAX IV synchrotron.

### DFT calculations

The electronic structure calculations for NdAlSi were performed based on the DFT^41,42^ as implemented in the VASP package^43,44^. The exchange-correlation functional was treated within the generalized gradient approximation in the form of Perdew–Burke–Ernzerhof^45^. The projector augmented wave method^46,47^ was used to describe the electron-ion interactions. Since the measurement temperature is above the Curie temperature, NdAlSi is non-magnetic; therefore, the Nd pseudopotential without 4f electrons was employed in the calculations. A plane-wave kinetic energy cutoff of 360 eV was used. BZ sampling was performed using a 6 × 6 × 6 Monkhorst-Pack k-point grid ^48^. A Gaussian smearing width of 0.1 eV was applied to approximate the Fermi-Dirac distribution. Spin-orbit coupling (SOC) was included as a second-order perturbation.

The bulk Fermi surface of NdAlSi was computed using a 60 × 60 × 60 k-point grid including SOC. The projection of bulk states onto the plane for different *k*-space slices was calculated via the Green’s function method, using a Fermi broadening of 0.01 eV to obtain the spectral function.

For the (103) surface states and Fermi arcs, we employed an iterative Green’s function method to model a semi-infinite surface and compute its surface states. Among the four possible terminations of the (103) surface, the Si-Al plane termination yielded results consistent with experimental observations.

## Supplementary information

Supplementary Information

Transparent Peer Review file

## Data Availability

The authors declare that all data supporting the findings of this study are available within the paper and its Supplementary Information files.
