# Supplementary material for: Topological Fermi arc interference on low-symmetry Weyl surfaces
Source: Nat Commun. 2026 Aug 13;17:9706. doi: 10.1038/s41467-026-76639-5 (PMC13562659; doi:10.1038/s41467-026-76639-5)
Supplement: Supplementary file 1 [file 41467_2026_76639_MOESM1_ESM.pdf]

Supplementary Information for

Topological Fermi Arc Interference on Low-Symmetry Weyl Surfaces

Cong Li[1,2,♯,*], Zhilong Yang[3,♯,*], Hongxiong Liu[1], Magnus H. Berntsen[2], Francesco Scali[4], Dibya Phuyal[2], Jianfeng Zhang[1], Timur K. Kim[5], Jacek Osiecki[6], Balasubramanian Thiagarajan[6], Youguo Shi[1], Tao Xiang[1], Quansheng Wu[1], Oscar Tjernberg[2,*]

[1]*Beijing National Laboratory for Condensed Matter Physics,*
Institute of Physics, Chinese Academy of Sciences, Beijing 100190, China

[2]*Department of Applied Physics, KTH Royal*
Institute of Technology, Stockholm 11419, Sweden

[3]*School of Mathematics and Physics,*
University of Science and Technology Beijing, Beijing 100083, China

[4]*Dipartimento di Fisica, Politecnico di Milano,*
Piazza Leonardo da Vinci 32, 20133 Milano, Italy

[5]*Diamond Light Source, Harwell Campus,*
Didcot, OX11 0DE, United Kingdom

[6]*MAX IV Laboratory, Lund University, 22100 Lund, Sweden*

[♯]*These people contributed equally to the present work.*

[*]*Corresponding authors: licong@iphy.ac.cn, zhlyang@ustb.edu.cn, oscar@kth.se*

1. Distinguishing surface states from bulk states

To verify the origin of the observed states, we systematically measured the Fermi surface of NdAlSi (103) surface using different photon energies, as shown in Supplementary Information Figure 1. The persistence of identical features across photon energies confirms that the observed signals originate from surface states (SSs) rather than bulk states (BSs). This establishes that the Fermi surface shown in Fig. 1f in the main text are indeed intrinsic to the SS of (103) surface.

Supplementary Information Figure 2 presents the X-ray photoemission spectroscopy (XPS) spectra measured with a photon energy of 200 eV, showing nearly identical core-level features for both flat and uneven surfaces, consistent with an unchanged chemical termination. A weak additional shoulder peak appears near the Si 2p core level on the uneven surface, indicating slight modifications of the local chemical environment that may arise from surface disorder. Nevertheless, the overall spectral shape and peak positions remain essentially the same. These results confirm that the suppression of SSs on uneven surfaces (Fig. 1e in the main text) is not due to a change in chemical termination, but rather originates from disorder-induced suppression of coherent SSs. Subsequent photon energy dependent measurements further confirm the bulk nature of the Fermi surface shown in Fig. 1e in the main text.

2. Polarization dependent SS and dichroism measurements of NdAlSi (103) surface

Supplementary Information Figures 3a-3d display the measured Fermi surfaces of the NdAlSi (103) surface under linear horizontal (LH), linear vertical (LV), positive circular (PC), and negative circular (NC) polarizations. The overall features remain robust across different incident polarizations, reflecting their intrinsic electronic origin. Supplementary Information Figures 3e and 3f present the corresponding linear dichroic (LD = (LH–LV)/(LH+LV)) and circular dichroic (CD = (PC–NC)/(PC+NC)) Fermi surfaces, respectively. The strong dichroic contrast highlights orbital-selective contributions and provides an additional handle for disentangling SSs from bulk bands, as well as for tracking the evolution of topological SSs.

3. Bulk Fermi surfaces of NdAlSi (103) surface

Supplementary Information Figures 4–6 compare photon energy dependent bulk Fermi surface measurements (Supplementary Information Figure 4) with DFT calculations of momentum-space slices along [103] (Supplementary Information Figure 5) and perpendicular to [103] (Supplementary Information Figure 6). The measured Fermi surfaces exhibit pronounced out-of-plane dispersion, with spectral features shifting systematically as photon energy varies clear evidence of bulk-derived states. The corresponding DFT slices taken along and perpendicular to the [103] direction capture the same overall topology of the Fermi-surface pockets, their relative distribution in the surface plane, and the periodic shifts with changing $k_\perp$ and $k_{//}$. The close correspondence between experiment and theory confirms the bulk origin of these features and further illustrates the quasi-periodic in-plane translations of bulk projections that are essential for understanding the superlattice periodicity discussed in the main text.

4. Commensurability of bulk projections with surface Brillouin zone periodicity

The periodicity of the two-dimensional surface Brillouin zone (SBZ) is determined by the primitive in-plane reciprocal vectors associated with a given facet. To analyze the relation between bulk and surface periodicities, let $\{\mathbf{b}_1, \mathbf{b}_2, \mathbf{b}_3\}$ denote the primitive reciprocal vectors of the bulk lattice with metric tensor

$$M_{ij} = \mathbf{b}_i \cdot \mathbf{b}_j,$$

which encodes the lattice constants and inter-axial angles. For a surface defined by Miller indices (hkl), the bulk reciprocal vector normal to the plane is

$$\mathbf{G} = h\,\mathbf{b}_1 + k\,\mathbf{b}_2 + l\,\mathbf{b}_3.$$

The projector onto the surface plane is

$$P = \mathbb{I} - \frac{\mathbf{G} \otimes \mathbf{G}}{\mathbf{G} \cdot \mathbf{G}}.$$

We choose the surface-BZ primitive basis as

$$\mathbf{g}_1 = P\mathbf{b}_1, \qquad \mathbf{g}_2 = P\mathbf{b}_2.$$

Any bulk translation projected onto the surface, for example $P\mathbf{b}_3$, can be decomposed as

$$P\mathbf{b}_3 = a_1\,\mathbf{g}_1 + a_2\,\mathbf{g}_2. \qquad (1)$$

a. From the decomposition to a linear system. Taking the inner product of Eq. (1) with $\mathbf{g}_1$ and $\mathbf{g}_2$ gives

$$\mathbf{g}_1 \cdot (P\mathbf{b}_3) = a_1(\mathbf{g}_1 \cdot \mathbf{g}_1) + a_2(\mathbf{g}_1 \cdot \mathbf{g}_2), \tag{2}$$

$$\mathbf{g}_2 \cdot (P\mathbf{b}_3) = a_1(\mathbf{g}_2 \cdot \mathbf{g}_1) + a_2(\mathbf{g}_2 \cdot \mathbf{g}_2). \tag{3}$$

Equations (2)–(3) are exactly the 2×2 linear system written in matrix form as

$$\underbrace{\begin{pmatrix} \mathbf{g}_1 \cdot \mathbf{g}_1 & \mathbf{g}_1 \cdot \mathbf{g}_2 \\ \mathbf{g}_2 \cdot \mathbf{g}_1 & \mathbf{g}_2 \cdot \mathbf{g}_2 \end{pmatrix}}_{G \text{ (Gram matrix)}} \begin{pmatrix} a_1 \\ a_2 \end{pmatrix} = \begin{pmatrix} \mathbf{g}_1 \cdot (P\mathbf{b}_3) \\ \mathbf{g}_2 \cdot (P\mathbf{b}_3) \end{pmatrix}. \tag{4}$$

Because $\mathbf{g}_1, \mathbf{g}_2$ are linearly independent for any well-defined facet, the Gram matrix G is symmetric positive-definite and hence invertible. Then, the coefficients (a_1, a_2) are obtained by solving the 2×2 linear system.

b. Closed-form solution for (a_1, a_2). Let

$$\Delta = \det G = (\mathbf{g}_1 \cdot \mathbf{g}_1)(\mathbf{g}_2 \cdot \mathbf{g}_2) - (\mathbf{g}_1 \cdot \mathbf{g}_2)^2 > 0.$$

Using Cramer's rule (or G^{-1}) yields

$$a_1 = \frac{(\mathbf{g}_2 \cdot \mathbf{g}_2)\left[\mathbf{g}_1 \cdot (P\mathbf{b}_3)\right] - (\mathbf{g}_1 \cdot \mathbf{g}_2)\left[\mathbf{g}_2 \cdot (P\mathbf{b}_3)\right]}{\Delta}, \tag{5}$$

$$a_2 = \frac{(\mathbf{g}_1 \cdot \mathbf{g}_1)\left[\mathbf{g}_2 \cdot (P\mathbf{b}_3)\right] - (\mathbf{g}_1 \cdot \mathbf{g}_2)\left[\mathbf{g}_1 \cdot (P\mathbf{b}_3)\right]}{\Delta}. \tag{6}$$

If an orthogonalized surface basis is chosen such that $\mathbf{g}_1 \cdot \mathbf{g}_2 = 0$, Eqs. (5)–(6) simplify to

$$a_1 = \frac{\mathbf{g}_1 \cdot (P\mathbf{b}_3)}{\|\mathbf{g}_1\|^2}, \qquad a_2 = \frac{\mathbf{g}_2 \cdot (P\mathbf{b}_3)}{\|\mathbf{g}_2\|^2}.$$

In the special case of an orthogonal bulk lattice (cubic, tetragonal, or orthorhombic), these coefficients can be written in closed form directly in terms of the Miller indices (hkl). One finds

$$a_1 = -\frac{h}{l}, \qquad a_2 = -\frac{k}{l}, \quad (l \neq 0),$$

which shows that the in-plane displacement per bulk BZ step is controlled entirely by the ratios of Miller indices. If $l = 0$, then we need to consider selecting other primitive basis vectors.

c. Commensurability along the out-of-plane stacking direction. Here we ask whether the projected shift between successive bulk BZs along the surface normal closes after a finite number of repetitions. This amounts to finding the smallest $n \in \mathbb{N}$ such that

$$na_1 \in \mathbb{Z}, \quad na_2 \in \mathbb{Z}.$$

If $a_i = p_i/q_i \in \mathbb{Q}$ in lowest terms, define $\mathrm{den}(a_i) = q_i$; if $a_i = 0$, then $\mathrm{den}(0) = 1$. If either a_i is irrational, $N_{\min} = \infty$. Otherwise,

$$N_{\min} = \mathrm{lcm}\big[\mathrm{den}(a_1), \mathrm{den}(a_2)\big].$$

Here, lcm denotes the least common multiple (LCM). With $\mathrm{den}(0) = 1$, the definition remains consistent for zero, integer, and rational coefficients, while irrational values yield $N_{\min} = \infty$. Thus, $N_{\min}$ reflects only the closure of the one-dimensional stacking sequence.

d. Minimal surface repeat unit for computation. An immediate corollary of the above is that the surface cell enlarged by $N_{\min}$ along directions where a_i has a nontrivial denominator is exactly the *minimal surface repeat unit* commensurate with bulk projections. This cell is therefore the correct choice for DFT slab or surface Green's-function calculations. Any smaller cell would fail to satisfy the bulk–surface commensurability condition and thus misrepresent the surface-state periodicity.

More explicitly, the logic is as follows: each successive bulk BZ projects into the SBZ displaced by $a_1\mathbf{g}_1 + a_2\mathbf{g}_2$. If a_i are rational, then after $N_{\min}$ repetitions the total displacement equals an integer linear combination of $\mathbf{g}_1$ and $\mathbf{g}_2$. This integer relation means that all distinct projections are now identified modulo the SBZ lattice. Hence, extending the surface unit cell by $N_{\min}$ along those directions generates a surface reciprocal lattice that exactly matches the closure of the projected bulk sequence. By contrast, choosing a smaller cell ($n < N_{\min}$) would imply that $na_1, na_2 \in \mathbb{Z}$, which contradicts the minimality of $N_{\min}$. Therefore, the $N_{\min}$-extended unit cell is not just sufficient but also necessary.

In practice, this prescription is straightforward:

1. Examine the coefficients (a_1, a_2);
2. If either is fractional, enlarge the corresponding real-space lattice vector by the least common multiple $N_{\min}$;
3. Construct the slab using this enlarged cell.

The resulting SBZ is guaranteed to be the smallest one consistent with bulk periodicity. This ensures that calculated surface spectral functions will display the correct band folding and SSs as observed in ARPES, without artificial replicas.

e. Finite-cutoff matching between adjacent SBZs. The geometric commensurability discussed above (with period $N_{\text{min}} = \text{lcm}[\text{den}(a_1), \text{den}(a_2)]$) guarantees that the *translation class* of bulk-to-surface projections closes after N_{min} bulk BZs, i.e. $N_{\text{min}}\, P\mathbf{b}_\perp \in \Lambda_{\text{SBZ}}$. However, when one compares the *finite-sum* projection obtained by summing only over a truncated set of bulk zones $\Omega = \{1, 2, \ldots, M\}$, the content inside two adjacent SBZs generally differs unless the index set Ω is invariant under the shift $m \mapsto m + N_{\text{min}}$ induced by moving from SBZ_n to SBZ_{n+1}. Formally, if $S(\Omega)$ denotes the projected set in SBZ_1 from indices Ω, then the projected set in SBZ_2 is $S(\Omega+N_{\text{min}})$ with $\Omega+N_{\text{min}} = \{m+N_{\text{min}} \,|\, m \in \Omega\}$. To have *exact* equality $S(\Omega) = S(\Omega + N_{\text{min}})$ at finite cutoff, one needs $\Omega = \Omega + N_{\text{min}}$. The smallest nonempty finite set with this property is two consecutive periods:

$$\Omega_{\text{min}} = \{1, 2, \ldots, 2N_{\text{min}}\},$$

so the minimal cutoff ensuring equality between SBZ_1 and SBZ_2 under a finite projection is

$$M_{\text{min}}^{(\text{adj})} = 2N_{\text{min}}.$$

In the infinite-sum limit ($\Omega = \mathbb{Z}$) this equality holds trivially.

Two notions of commensurability. It is important to distinguish two related but not identical concepts:

1. *Geometric commensurability.* The parameter N_{min} reflects the closure of the one-dimensional stacking sequence defined by the projected bulk reciprocal lattice vectors. This purely geometric treatment considers only the relation between $P\mathbf{b}_i$ and the primitive basis of the SBZ, and establishes whether the projected periodicities close after a finite number of bulk BZs (commensurate case) or only in the infinite limit (incommensurate case).

2. *Spectral commensurability.* In practice, the bulk electronic structure–such as the distribution of Weyl points or band extrema–does not necessarily preserve the full symmetry of the BZ. Consequently, projections from higher-order bulk zones are not strictly

equivalent to those from the first, and additional zones (as discussed above, typically up to $2N_{\min}$) are required to reproduce even partial spectral equivalence. Moreover, because the bulk reciprocal lattice generates the entire set $\{m_1\mathbf{b}_1 + m_2\mathbf{b}_2 + m_3\mathbf{b}_3\}$, all of which project onto the surface, exact reconstruction of the full SBZ periodicity requires contributions from *infinitely many* bulk BZs. Thus, no finite cutoff can fully capture all projected Weyl points and surface-state replicas across the two-dimensional SBZ.

Example: (103) surface of tetragonal NdAlSi

For $(h, k, l) = (1, 0, 3)$ in tetragonal NdAlSi ($a = b = 4.204$ Å, $c = 14.519$ Å[1]) one obtains

$$P\mathbf{b}_3 = -\tfrac{1}{3}\,\mathbf{g}_1 + 0 \cdot \mathbf{g}_2,$$

so $a_1 = -1/3$, $a_2 = 0$, and hence

$$N_{\min} = \mathrm{lcm}(3, 1) = 3,$$

independent of the specific values of a and c. Thus, along the out-of-plane stacking direction, the projected shift closes exactly after three bulk BZs ($N_{\min} = 3$). This means that each successive bulk BZ projects onto the (103) plane as a lateral shift of $-1/3g_1$. As a result, the first, second, and third bulk BZs contribute distinct projections within the first SBZ, while the fourth bulk BZ closes the cycle by returning to a displacement of $-g_1$, which is equivalent to the first.

Worked application of the prescription. Applying the minimal repeat unit rule:

- **Step 1 (identify fractional directions):** $a_1 = -1/3$ is fractional, while $a_2 = 0$ is integer.

- **Step 2 (build the minimal cell):** enlarge the real-space surface lattice vector dual to $\mathbf{g}_1$ by a factor of 3; keep the vector dual to $\mathbf{g}_2$ primitive. The slab in-plane cell is therefore 3×1 with respect to the primitive (103) surface lattice.

- **Step 3 (compute):** construct a slab using this 3×1 in-plane cell, with sufficient thickness and vacuum. For Wannier-based semi-infinite Green's functions, choose a

principal layer compatible with the 3×1 periodicity. The resulting surface spectral function $A(\mathbf{k}_{\parallel}, \omega)$ reproduces the experimentally observed SBZ closure and the correct placement of SSs; a primitive (1×1) cell would instead yield mismatches and spurious folding.

Notes.

(i) If multiple chemical terminations exist, compute each with the same 3×1 periodicity and compare or mix them according to experiment.

(ii) Weak surface relaxation does not alter $N_{\min}$ (a property of the metric/projection), but can slightly shift surface bands; relaxing only the top few layers is usually sufficient.

(iii) If a true real-space reconstruction exists (e.g., 5×1), that reconstruction–not the LCM cycle–sets the minimal computational cell.

Remark on conventional fcc indexing. Since NdAlSi is *body-centered tetragonal (bct)* in real space, its reciprocal lattice is *face-centered tetragonal (fct)*. Under the conventional fct indexing rule (all-even/all-odd hkl), the nearest allowed reciprocal point along [001] is (002) rather than (001). Consequently, the bulk first-BZ half-width along the surface normal (k_z) doubles (e.g. from ± 0.2164 to ± 0.4328 Å^{-1}, total 0.8656 Å^{-1}). This change affects only the normal direction and does not alter the in-plane SBZ' size $(\mathbf{g}'_1, \mathbf{g}_2)$. In this convention, adjacent bulk BZs separated by $2\mathbf{b}_3$ yield an in-plane shift

$$P(2\mathbf{b}'_3) = 2\,P\mathbf{b}'_3 = -\tfrac{2}{3}\,\mathbf{g}'_1,$$

which is equivalent to the primitive-basis statement $P\mathbf{b}'_3 = -\frac{1}{3}\,\mathbf{g}'_1$ and does not modify $N_{\min}$. Equivalently, the minimal commensuration along the $\mathbf{g}'_1$ direction reads

$$3 \times \left(\tfrac{2}{3}\,\mathbf{g}'_1\right) = 2\,\mathbf{g}'_1$$

i.e. three bulk projections commensurate with two $2\,\mathrm{SBZ}'$. Hence, in this conventional fct enumeration the minimal in-plane supercell spans $2\,\mathrm{SBZ}'$ widths along $\mathbf{g}'_1$ (and 1 along $\mathbf{g}_2$). To keep the apparent three-to-one commensuration explicit under the conventional fct notation, we *identify* $2\,\mathrm{SBZ}'$ as the (redefined) SBZ and set $\mathbf{g}_1 \equiv 2\mathbf{g}'_1$. In this convention, the bulk primitive basis vectors become $\mathbf{b}_1 \equiv 2\mathbf{b}'_1$ and $\mathbf{b}_3 \equiv 2\mathbf{b}'_3$. With this unified notation, the statement "three bulk projections match one surface unit" remains valid.

Notes. According to the same convention (i.e., the conventional fct indexing rule requiring all-even or all-odd hkl), the nearest allowed reciprocal point along [001] is (002) rather than (001). Strictly speaking, and for the sake of notational consistency, this implies that the SBZ should also be doubled along $\mathbf{g}_2'$ (i.e., $\mathbf{g}_2 \equiv 2\mathbf{g}_2'$). However, since this direction is commensurate, we do not elaborate further so as to keep the focus on the incommensurate direction, whose treatment already captures the essential physics and avoids additional notational complexity.

Layer-changing second-BZ neighbors. Defining the "second-order BZ" as all six neighbors adjacent to the first (labels #1–#6 in Supplementary Information Figure 7a), their surface projections populate the in-plane displacements

$$\{ \pm\tfrac{1}{3}\,\mathbf{g}_1,\ \pm\tfrac{2}{3}\,\mathbf{g}_1 \} \ \oplus\ \mathbb{Z}\,\mathbf{g}_2.$$

Thus #1 and #5 map to $+\frac{1}{3}\,\mathbf{g}_1$, #2 and #6 to $-\frac{1}{3}\,\mathbf{g}_1$, while #3 and #4 correspond to $\pm\frac{2}{3}\,\mathbf{g}_1$. The latter coincide in position with third–BZ copies at $\pm\frac{2}{3}\,\mathbf{g}_1$, explaining the diagonal offsets visible in Supplementary Information Figure 7a.

It is important to note, however, that such "position coincidence" does not imply full spectral equivalence. The second-order neighbors do not reproduce the multiplicity and symmetry of contributions from higher orders: e.g., the $\pm\frac{2}{3}\,\mathbf{g}_1$ shifts appear only once in the second-order set, whereas the corresponding $\pm\frac{2}{3}\,\mathbf{g}_1$ displacements in the third-order set occur in a different multiplicity pattern. Consequently, the projected features inside SBZ_1 and SBZ_2 are still not identical if only the second-order BZs are included. In other words, although some higher-order points are "mimicked" in position by lower-order neighbors, their relative weights and degeneracies remain unmatched.

Geometric closure remains $N_{\min} = 3$, reflecting that three successive bulk projections along the (103) normal return to an equivalent in-plane shift. To enforce strict visual identity between adjacent SBZs under a finite projection, one must adopt a symmetric cutoff that includes both positive and negative orders up to

$$M_{\min}^{(\mathrm{adj})} = 2N_{\min} = 6.$$

The distinction between the geometric cycle ($N_{\min} = 3$) and the finite-cutoff condition ($M_{\min} = 6$) accounts for this observation. As one proceeds to even higher orders, the set

of contributing bulk BZs expands further; no finite cutoff can reproduce the full spectral content exactly. Only in the infinite limit does the superlattice generated by all bulk BZ projections become exactly commensurate with the SBZ. This is why the surface periodicity is progressively restored as more bulk BZs are added, while exact closure is only reached in the infinite-projection limit.

Example: (001) surface of tetragonal NdAlSi

For $(h, k, l) = (0, 0, 1)$ one finds $P\mathbf{b}_3 = 0$, so $a_1 = a_2 = 0$ and $N_{\min} = 1$. Both geometric and spectral commensurability are satisfied immediately, and already the first bulk BZ reproduces the full surface periodicity.

Remarks on non-orthogonal lattices

For non-orthogonal systems (e.g. hexagonal, rhombohedral, monoclinic), the coefficients (a_1, a_2) depend explicitly on ratios such as a/c and interaxial angles. In such cases a_1 or a_2 may be irrational, leading to $N_{\min} = \infty$ even for the one-dimensional stacking sequence. This highlights the genuine metric dependence of the commensurability problem.

Visualization of bulk BZ projections on the (103) surface

Supplementary Information Figure 7 illustrates how bulk BZs project onto the low-symmetry (103) surface and generate an emergent superlattice periodicity. Supplementary Information Figure 7a shows the projection of Weyl points from the first and six second-order BZs onto the (103) plane. Due to the incommensurability between bulk reciprocal vectors and the (103) surface basis, consecutive BZ projections appear shifted within the surface plane, rather than coinciding as on high-symmetry surfaces. Supplementary Information Figure 7b presents a schematic stacking view: successive second-order BZs project as laterally displaced replicas, and their superposition yields a new periodicity–a surface-consistent superlattice (a momentum-space moiré modulation). This visualization highlights how including higher-order bulk BZ projections restores the apparent mismatch between bulk and surface periodicities, thereby resolving the bulk-boundary correspondence (BBC) paradox

on low-symmetry facets.

To further illustrate this mechanism, we explicitly integrate the bulk spectral weight from first-principles calculations over finite windows in momentum space. Supplementary Information Figure 8a shows the Fermi surface obtained by integrating planes parallel to the (103) surface within $k_{\perp}$ = 0-0.5 Å^{-1}, while Supplementary Information Figure 8b presents the result for perpendicular planes within $k_{//}$ = 0-1.5 Å^{-1}. For small integration ranges, the projected bulk features appear misaligned with the surface periodicity. As the integration window expands, contributions from multiple bulk BZs are superimposed, and the apparent periodicity progressively approaches that of the (103) SBZ. This provides a direct visualization of how higher-order bulk projections gradually restore the correct surface periodicity, consistent with the generalized framework established above.

This generalized framework demonstrates that the apparent breakdown of BBC on low-symmetry surfaces is not a failure of topology but a consequence of incomplete bulk projection. By superimposing successive bulk BZs, a superlattice periodicity emerges that is fully commensurate with the SBZ, thereby restoring consistency between bulk and SSs. An immediate implication of this incommensurability is that topological surface Fermi arcs (SFAs) originating from different bulk zones may no longer project in registry. Instead, they overlap and hybridize on the surface, leading to new boundary features that have no analogue on high-symmetry facets. In particular, such hybridization provides a natural pathway for the formation of closed surface Fermi arc loops (SFALs), which we discuss in detail later.

5. Surface state calculations and comparisons

Supplementary Information Figure 9 illustrates possible cleavage terminations that may arise when exposing the (103) surface of NdAlSi. Supplementary Information Figure 9a shows one possible cleavage mode that results in a zigzag-shaped (103) surface. This geometry can be understood as choosing an equivalent cleavage layer within each primitive unit cell. Since the primitive cells are translationally equivalent, cleavage is expected to occur along symmetry-equivalent layers, leading naturally to zigzag-type terminations. Supplementary Information Figure 9b exhibits an alternative description that emerges when three consecutive primitive cells are grouped into a supercell. Within this enlarged supercell, four nonequivalent cleavage layers exist, corresponding to eight distinct terminations of the

(103) surface. The example in Supplementary Information Figure 9b illustrates one such construction, highlighting how the choice of supercell redefines the possible cleavage options. In this construction, the black parallelogram marks the minimal repeat unit of the (103) surface lattice. Because its spanning vectors are oblique, this cell is effectively monoclinic, despite the bulk crystal being body-centered tetragonal. This effective monoclinic character also shows why bulk and surface periodicities are intrinsically incommensurate when only a single surface cell is considered, and why the LCM criterion is required to restore commensuration. For the (103) surface of NdAlSi, this corresponds to a three-cell supercell that reconciles the SBZ with the projected bulk BZ.

In general, different choices of supercell lead to additional possibilities for the (103) cleavage geometry, and thus a wide variety of surface terminations may be realized. The actual cleavage plane adopted in experiment depends sensitively on the cleavage energy associated with each candidate layer: lower cleavage energy corresponds to a more favorable termination. Importantly, regardless of which cleavage mode is realized, a consistent description of the SSs requires that the LCM criterion be satisfied. Only by adopting a supercell commensurate with the LCM rule can the reconstructed SBZ and the associated topological SSs be described correctly.

Experimentally, ARPES measurements confirm the coexistence of multiple surface terminations. As shown in Supplementary Information Figure 10, besides the dominant Fermi surface structure in Supplementary Information Figure 10a, two additional distinct SS patterns were detected (Supplementary Information Figures 10b-10c). Together, these observations reveal at least three different SS configurations coexisting on the cleaved (103) surface. Importantly, these distinct spectra were obtained from different spatial regions of the same cleaved surface, demonstrating that a single macroscopic cleavage can naturally expose multiple microscopic terminations. Notably, despite their distinct dispersions, all three observed terminations exhibit the same reduced surface Brillouin zone periodicity, directly confirming that the LCM criterion governs SS commensuration independent of termination details. This experimentally confirms the universality of the LCM criterion established in Section 4. This consistency underscores the robustness of the LCM framework across different microscopic terminations. At the same time, we cannot exclude the possibility that even more variants may exist, consistent with the complexity expected for low-symmetry surfaces and the potential emergence of long-period zigzag-type reconstructions.

Based on this principle, the SS calculations shown in Supplementary Information Figure 11 were carried out using the four possible cleavage layers identified within the three-cell supercell construction of Supplementary Information Figure 9b. This approach ensures that the computed surface spectra are both physically meaningful and directly comparable with experimental observations. Supplementary Information Figures 11 and 12 present the calculated surface Fermi surfaces for the (103) orientation of NdAlSi, considering four possible cleavage planes corresponding to eight distinct surface terminations. After extensive testing of these possibilities, we find that the calculation for a Si-terminated surface cleaved at the Al-Si layer (Supplementary Information Figure 11b) reproduces the experimentally measured dominant surface Fermi surface most accurately (Supplementary Information Figure 10a), confirming that the probed region corresponds to this termination. Nonetheless, the additional surface structures observed in Supplementary Information Figures 10b-10c do not find convincing counterparts among the terminations considered here, indicating that their microscopic origin remains to be established. They are likely associated with more complex long-period zigzag-type cleavages that go beyond the four-layer options of the three-cell supercell in Supplementary Information Figure 9b, or with spatially mixed terminations within the probed regions.

Nonetheless, two important discrepancies remain. First, the additional surface structures observed in Supplementary Information Figures 10b–10c do not find convincing counterparts among the terminations considered here, indicating that their microscopic origin lies beyond the simple three-cell construction. They are likely associated with more complex long-period zigzag-type cleavages or with spatially mixed terminations within the probed regions. Second, even for the well-matched Si-terminated surface, the idealized calculations do not explicitly reproduce the closed connectivity of SFALs seen in ARPES. We attribute this mismatch to two factors: (i) the neglect of zigzag-type cleavages and mixed local terminations, which in real samples promote enhanced coupling between surface and bulk states; and (ii) the incomplete treatment of hybridization across multiple projected bulk BZs, whose lateral mismatch favors loop-like reconstructions of SSs. These considerations emphasize that while the LCM-consistent modeling captures the reduced periodicity and dominant spectral features, certain finer aspects of the connectivity remain emergent properties of real low-symmetry surfaces beyond the reach of ideal slab calculations.

Supplementary Information Figure 11 shows the results obtained using the Green's func-

tion method, which treats the system as semi-infinite and therefore automatically incorporates contributions from infinitely many bulk BZs. The output is a surface-projected spectral function. As a result, the "bulk" features appearing in these calculations are SBPSs, which inevitably couple to the boundary. When such projected BSs overlap with SSs in energy and momentum, they acquire mixed character–retaining bulk-like dispersion while carrying finite spectral weight at the surface. These mixed features correspond precisely to the SBRSs observed in ARPES.

By contrast, Supplementary Information Figure 12 shows the results of a finite six-layer slab calculation in real space. Because the finite slab thickness quantizes the out-of-plane momentum $k_\perp$, only a discrete set of bulk states is sampled, which cannot reproduce the continuous bulk projections underlying genuine SBRSs. As a result, the bulk-like features are truncated and fail to hybridize correctly with the surface. Consequently, the agreement with experiment is poorer for the (103) surface, where the incommensurability between surface and bulk periodicities requires contributions from infinitely many bulk projections. Nevertheless, slab calculations remain useful for identifying SSs, and their degree of reliability depends strongly on the surface orientation.

This comparison highlights a fundamental distinction between low- and high-symmetry surfaces. For low-symmetry facets such as (103), Green's function calculations are indispensable for capturing both SSs and SBRSs, since only the semi-infinite approach properly restores the surface periodicity. In contrast, for high-symmetry surfaces such as (001), the bulk and SBZs are commensurate; in such cases, finite slabs already reproduce the correct periodicity, and while they do not describe resonance states, their calculated SSs often agree better with experiment than those from the Green's function method[2].

In this way, the present framework not only clarifies why Green's function calculations succeed while finite slabs fail for low-symmetry surfaces, but also provides general guidance for future computational studies: slab models may suffice for high-symmetry facets, whereas low-symmetry surfaces intrinsically require semi-infinite Green's function methods to faithfully capture their electronic structure.

6. Identification and evolution of topological SFAs

Identification of SFAs

Supplementary Information Figures 13a and 13b show the Fermi surfaces measured with

LH and LV polarizations, with momentum cuts defined for further analysis. Supplementary Information Figures 13c-13f present dispersions along Cut1 and Cut2, which intersect the SFAs. The arc-like dispersions observed here are consistent with topological SFAs, as they do not close within a single SBZ. Supplementary Information Figures 13g-13j display dispersions along Loop1-Loop4 (L1-L4), which form closed contours in momentum space. In the conventional framework of BBC, the presence of a nonzero Chern number and projected chiral charge enclosed by such loops would provide unambiguous evidence for topological SFAs[2, 3].

To further clarify the SFA connectivity on the (103) surface, we performed semi-infinite Green's function calculations, as shown in Supplementary Information Figure 14. Supplementary Information Figure 14a presents the SBPSs, forming the continuum background. Supplementary Information Figure 14b shows the total surface spectrum, where both SBPSs and genuine SSs coexist. By subtracting the SBPS contribution in Supplementary Information Figure 14a, we obtain the pure SSs in panel Supplementary Information Figure 14c. Superimposed red and green markers indicate the projections of Weyl points from the first-order to third-order bulk BZs, with chirality $+1$ and -1, respectively. The comparison across Supplementary Information Figures 14a-14c highlights how projected Weyl points overlap with bulk resonances, how true SSs emerge on top of this continuum, and how the arc connectivity becomes evident once the pure surface contribution is isolated.

Taken together, the experimental identification of arc-like dispersions in Supplementary Information Figure 13, combined with the theoretical separation of SBPSs and pure SSs in Supplementary Information Figure 14, provides a consistent picture: the observed arcs are topological SFAs connecting Weyl points of opposite chirality. While certain fine details differ between the ARPES spectra and the DFT-based surface calculations, the overall agreement is good. This correspondence supports the robustness of our assignment and provides a useful framework for interpreting the overall reconstruction of surface states. We note, however, that the detailed connectivity of SFAs in experiment is not fully captured by the idealized calculations, reflecting additional hybridization across multiple bulk projections and possible surface complexity beyond the simple termination model.

Hybridization and emergence of SFALs

On the low-symmetry (103) surface, however, the situation becomes more subtle. Be-

cause successive bulk BZs project onto incommensurate positions, arcs from different zones can spatially overlap and hybridize. Such hybridization of surface Fermi arcs can occur in two ways. First, Weyl points from different bulk BZs may project onto the same surface location, while the SFA paths connecting them do not coincide, thereby forming an SFAL. Second, Weyl points from different bulk BZs project onto distinct surface locations, yet the projected surface Fermi arcs connecting them intersect and reconstruct on the surface, giving rise to a closed SFAL. In both cases, projection-induced coupling transforms open arcs into SFALs, which are no longer tied one-to-one to individual pairs of Weyl nodes. At the present highest ARPES resolution available to us, SFA1 and SFA2 in Supplementary Information Figure 13a-13b are seen to hybridize into a closed SFAL, and in addition a slender, elliptical closed SFAL (Supplementary Information Figure 13a) is discernible near the center of the SBZ. Clearer SFAL features can also be observed in the LD and CD Fermi surfaces shown in Supplementary Information Figure 3. As a result, the strict equality between the Chern number enclosed by a surface loop and the net projected chiral charge is relaxed. The bulk topology itself remains intact, but its manifestation on the surface is redistributed across an ensemble of hybridized states, including both open arcs and loops.

Generalized BBC on low-symmetry facets

This motivates a more general understanding of BBC: instead of a direct correspondence between individual arcs and Weyl-point pairs, the correspondence must be understood collectively. The bulk Chern numbers remain faithfully encoded in the SSs, but their representation may take the form of hybridized, loop-like structures. This picture is consistent with the multi-BZ projection framework developed in Section 4, where higher-order replicas generate a superlattice periodicity commensurate with the SBZ, thereby restoring BBC in a broadened form.

Physical implications of SFALs

The emergence of SFALs enriches the boundary topology of Weyl semimetals and opens new physical possibilities. Closed momentum-space loops act as natural k-space cyclotron paths, giving rise to Shubnikov-de Haas and de Haas-van Alphen oscillations, with frequencies set by loop areas and angular dependence reflecting low-symmetry geometry. Magnetic breakdown between neighboring loops or between loops and arcs may generate composite

oscillatory signatures. Overlapping loops also support phase-coherent interference, including Aharonov-Bohm-like oscillations in mesoscopic devices and interference patterns in pump-probe spectroscopy. Their extended and nearly parallel segments enhance nesting, favoring density-wave or pairing instabilities.

Moreover, reduced crystalline symmetry ensures that Berry-curvature contributions do not cancel, enabling finite Berry-curvature dipoles that can drive nonlinear Hall, rectification, and nonreciprocal transport responses. In proximity to superconductors, SFALs may mediate loop-selective Andreev reflection and unconventional Josephson interference distinct from open-arc transport. In STM quasiparticle interference, they should appear as sharp scattering vectors resembling conventional Fermi pockets, but decorated with spin-momentum locking. In nonequilibrium regimes, ultrafast ARPES could directly track their formation, hybridization, and recombination, offering insights into anisotropic relaxation and electron-phonon coupling.

7. Mismatched periodicities and emergent superlattices

The projection-induced formation of SFALs on low-symmetry facets can be understood within a broader context of emergent periodicities arising from mismatched lattices. A well-known analogue occurs in moiré physics of stacked two-dimensional (2D) materials, where slight lattice mismatches or relative rotations generate emergent moiré superlattices that fundamentally reshape the electronic structure. These moiré patterns illustrate how new periodicities emerge from incommensurate or nearly commensurate structures, giving rise to flat bands, correlated phases, and unconventional topological responses.

In crystalline Weyl semimetals, low-symmetry facets provide a closely related scenario: the mismatch between surface and bulk periodicities leads to apparent inconsistencies in BBC. These inconsistencies are resolved once multiple bulk BZ projections are taken into account, whose superposition generates emergent superlattices commensurate with the surface. This least common multiple construction of reciprocal vectors offers a systematic way to restore periodicity and reconcile surface and bulk descriptions.

From this viewpoint, our framework can be naturally interpreted as establishing a momentum-space moiré pattern. Each successive bulk BZ projection introduces a lateral shift relative to the SBZ, analogous to a real-space lattice mismatch. The accumulation of these shifts produces a long-period modulation in momentum space—a "moiré stripe"

pattern—that governs how Fermi arcs overlap, hybridize, and evolve into closed loops. Thus, the apparent paradox of BBC on low-symmetry facets is resolved by recognizing that the boundary spectrum is shaped by this emergent momentum-space moiré superlattice.

This framework naturally extends to quasicrystals, which can be viewed as the extreme case of incommensurability. In quasicrystals, the reciprocal vectors form an intrinsically incommensurate set, so that no finite number of bulk BZs suffices to restore commensurability with the surface. Instead, quasi-periodicity emerges only in the infinite projection limit, where the dense set of reciprocal vectors reconstructs the correct boundary spectrum. From this viewpoint, quasicrystals are the limiting case of our framework: high-symmetry surfaces correspond to perfectly commensurate projections, low-symmetry surfaces require multiple bulk BZs to restore commensurability, and quasicrystals represent the fully incommensurate limit where only infinite projection recovers quasi-periodicity.

This unified perspective highlights a general principle: mismatched periodicities–whether in moiré bilayers, momentum-space moiré stripes on low-symmetry crystal facets, or quasicrystals–generate emergent superlattices that govern hybridization and boundary states. In all cases, the interplay of commensurability, projection, and boundary conditions produces new forms of electronic structure beyond those expected from the parent lattice. The framework developed here therefore provides a common language that connects moiré materials, crystalline surfaces of arbitrary orientation, and quasicrystalline systems, offering broad opportunities to uncover hidden boundary phenomena and design exotic responses across diverse classes of quantum matter.

8. Determination of surface-bulk resonance states

From a spectroscopic perspective, surface-bulk projected states (SBPSs) arise from projecting the three-dimensional (3D) bulk bands of the BZ onto the two-dimensional (2D) SBZ. In surface-sensitive probes such as ARPES, this projection appears as the integrated spectral weight over the out-of-plane momentum,

$$A_{\mathrm{proj}}(\mathbf{k}_{\parallel}, E) \;=\; \int \frac{dk_{\perp}}{2\pi}\, W(\mathbf{k}_{\parallel}, k_{\perp})\, A_{\mathrm{bulk}}(\mathbf{k}_{\parallel}, k_{\perp}, E),$$

where A_{bulk} is the bulk spectral function and $W(\mathbf{k}_{\parallel}, k_{\perp})$ encodes the weight of bulk Bloch states on the top surface layers. SBPSs therefore follow the periodicity of the SBZ, yet they are not new eigenstates but rather the continuum projection of BSs, and they appear in

ARPES as diffuse spectral backgrounds.

By contrast, SSs are sharp eigenmodes localized near the surface that exist inside bulk-projected band gaps. BSs, on the other hand, are fully extended Bloch states with negligible surface weight, which dominate spectra on strongly disordered or rough surfaces. When a SS lies outside the SBPS continuum, it remains sharp and well defined. However, when a SS enters the bulk-projected region, it hybridizes with the underlying bulk continuum and evolves into a surface-bulk resonance state (SBRS). SBRSs extend throughout the bulk but retain finite amplitude near the surface, enabling them to couple to surface-sensitive probes. As a result, they appear in ARPES as boundary-related features with surface periodicity but broader linewidths compared to pure SSs.

The identification of SBRSs requires separating them from SSs, SBPSs, and BSs. To achieve this, we adopt a sequence of complementary strategies.

First, introducing surface disorder selectively suppresses coherent SSs while leaving SBPSs comparatively unaffected, since the latter originate from bulk projection. As shown in Supplementary Information Figure 15, deliberate adsorption of impurities onto initially flat surfaces strongly suppresses SSs, revealing the underlying SBPSs. Importantly, the diffuse SBPS features remain nearly unchanged between photon energies of 41 eV (Supplementary Information Figure 15a, 15c and 15d) and 53 eV (Supplementary Information Figure 15b, 15e and 15f), apart from variations in spectral weight caused by matrix-element effects or changes in the photon penetration depth, which alter the relative bulk versus near-surface contribution. This robustness further confirms the bulk-projected nature of SBPSs, in contrast to SSs that are highly sensitive to surface disorder.

Second, systematic comparison of different surface conditions distinguishes SSs, SBPSs, and BSs. As summarized in Supplementary Information Figure 16, (i) pristine flat surfaces are dominated by sharp SSs (Supplementary Information Figure 16a), (ii) moderately disordered surfaces expose SBPSs (Supplementary Information Figure 16b), and (iii) strongly disordered or rough surfaces suppress both SSs and SBPSs, leaving BSs dominant (Supplementary Information Figure 16c-16d). Notably, these comparisons also highlight a competition between SSs and SBPSs. On clean flat surfaces, coherent SSs dominate the spectral weight and partially obscure the diffuse SBPS background. When disorder is introduced, SSs are selectively suppressed, and the SBPS continuum becomes more visible. This redistribution of intensity indicates that SSs and SBPSs are not independent contributions

but strongly intertwined: SSs emerge as coherent boundary modes in the absence of strong bulk projection, SBPSs dominate when SSs are weakened, and at their overlap the competition evolves into hybridization, which naturally leads to SBRSs (demonstrated below in Supplementary Information Figure 18).

Third, photon energy dependence provides an additional check. As shown in Supplementary Information Figure 17, clean flat surfaces again display SS-dominated spectra (Supplementary Information Figure 17a), whereas mild disorder reveals SBPS features (Supplementary Information Figure 17b). On rough surfaces, BSs dominate instead (Supplementary Information Figure 17c). At intermediate photon energies of 130 eV-140 eV, disordered surfaces consistently show SBPS-dominated spectra (Supplementary Information Figures 17d–17f). These results confirm that SBPSs are robust projected continua, distinct from both SSs and BSs.

Finally, SBRSs are identified in Supplementary Information Figure 18, where photon energy dependent Fermi surface maps (41-53 eV) (Supplementary Information Figure 18a-18d) and corresponding dispersions (Supplementary Information Figure 18e-18l) along Cut1 and Cut2 are presented. When SSs overlap with the SBPS continuum, hybrid SBRS features emerge (Supplementary Information Figure 18a-18d), highlighted by their persistence across photon energies. The fact that SBRS dispersions remain nearly invariant with varying photon energies reinforces their identification as hybrid surface-bulk excitations, distinct from both SSs and pure BSs.

Taken together, the complementary evidence from Supplementary Information Figures 15-18 provides a clear and reproducible methodology for disentangling different classes of states. SSs are revealed on pristine surfaces, SBPSs appear as diffuse continua when SSs are suppressed, BSs dominate under strong disorder, and SBRSs emerge when SSs hybridize with the SBPS continuum. These results demonstrate that SBRSs form a distinct and intrinsic class of hybrid boundary excitations in NdAlSi and related Weyl semimetals.

Notably, the presence of pronounced SBRSs in NdAlSi may also explain why surface disorder is particularly effective in suppressing not only trivial SSs but also topological SSs (TSSs)[4]. Although TSSs are protected by bulk band topology, in NdAlSi they frequently overlap with bulk-projected continua and thus evolve into SBRSs. In this hybridized form, their surface localization is weakened, rendering them highly susceptible to surface disorder that selectively suppresses the surface component. As a result, the apparent fragility of

TSSs in ARPES is not inconsistent with their topological origin but rather reflects their strong hybridization with bulk continua through SBRS formation.

---

[1] C. Li *et al.*, Non-Hermitian Boundary in a Surface Selective Reconstructed Magnetic Weyl Semimetal. Adv. Mater. **37**, 2419559 (2025).

[2] C. Li *et al.*, Emergence of Weyl fermions by ferrimagnetism in a noncentrosymmetric magnetic Weyl semimetal. Nat. Commun. **14**, 7185 (2023).

[3] I. Belopolski *et al.*, Criteria for Directly Detecting Topological Fermi Arcs in Weyl Semimetals. Phys. Rev. Lett. **116**, 066802 (2016).

[4] C. Li *et al.*, Disorder-Driven Non-Anderson Transition in a Weyl Semimetal. PNAS **122**, e2508569122 (2025).

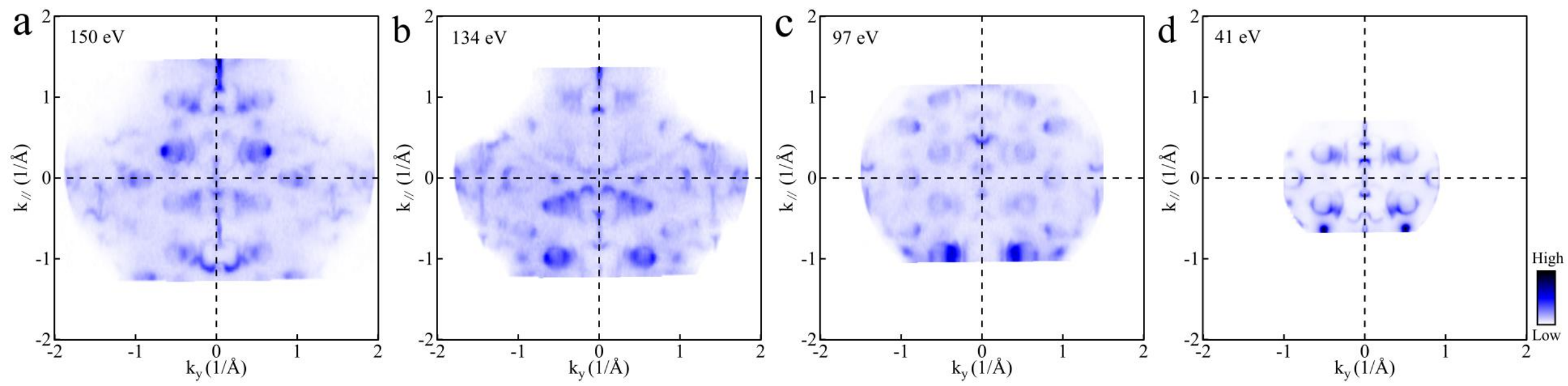

Supplementary Information Figure 1. **Photon energy dependent Fermi surface of NdAlSi (103) surface.** (a-d) Fermi surfaces of NdAlSi (103) surface measured on a flat (well defined) region at different photon energies: (a) 150 eV, (b) 134 eV, (c) 97 eV, and (d) 41 eV.

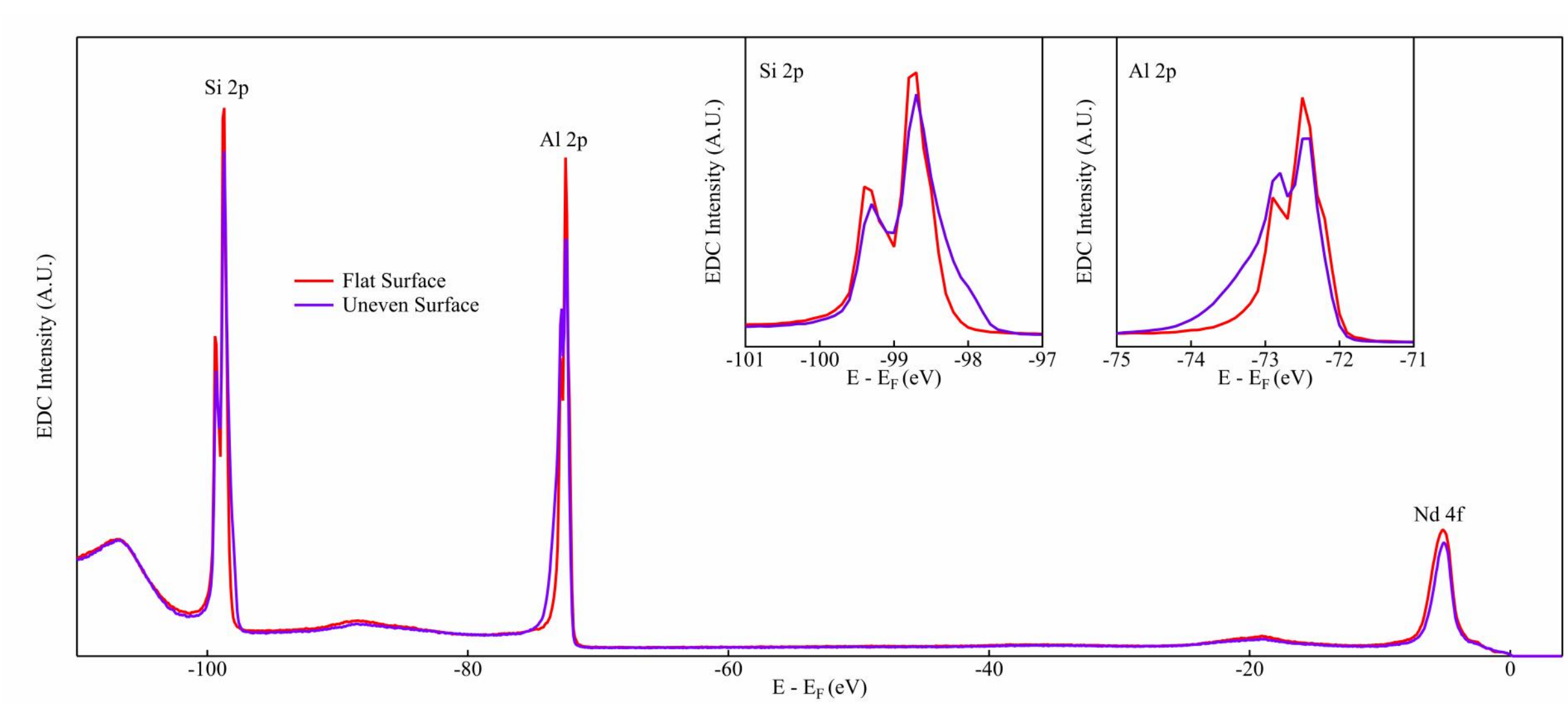

Supplementary Information Figure 2. **XPS of NdAlSi.** Comparison of core-level spectra from flat (red) and uneven (purple) surfaces. Insets show zoomed Si 2p and Al 2p peaks.

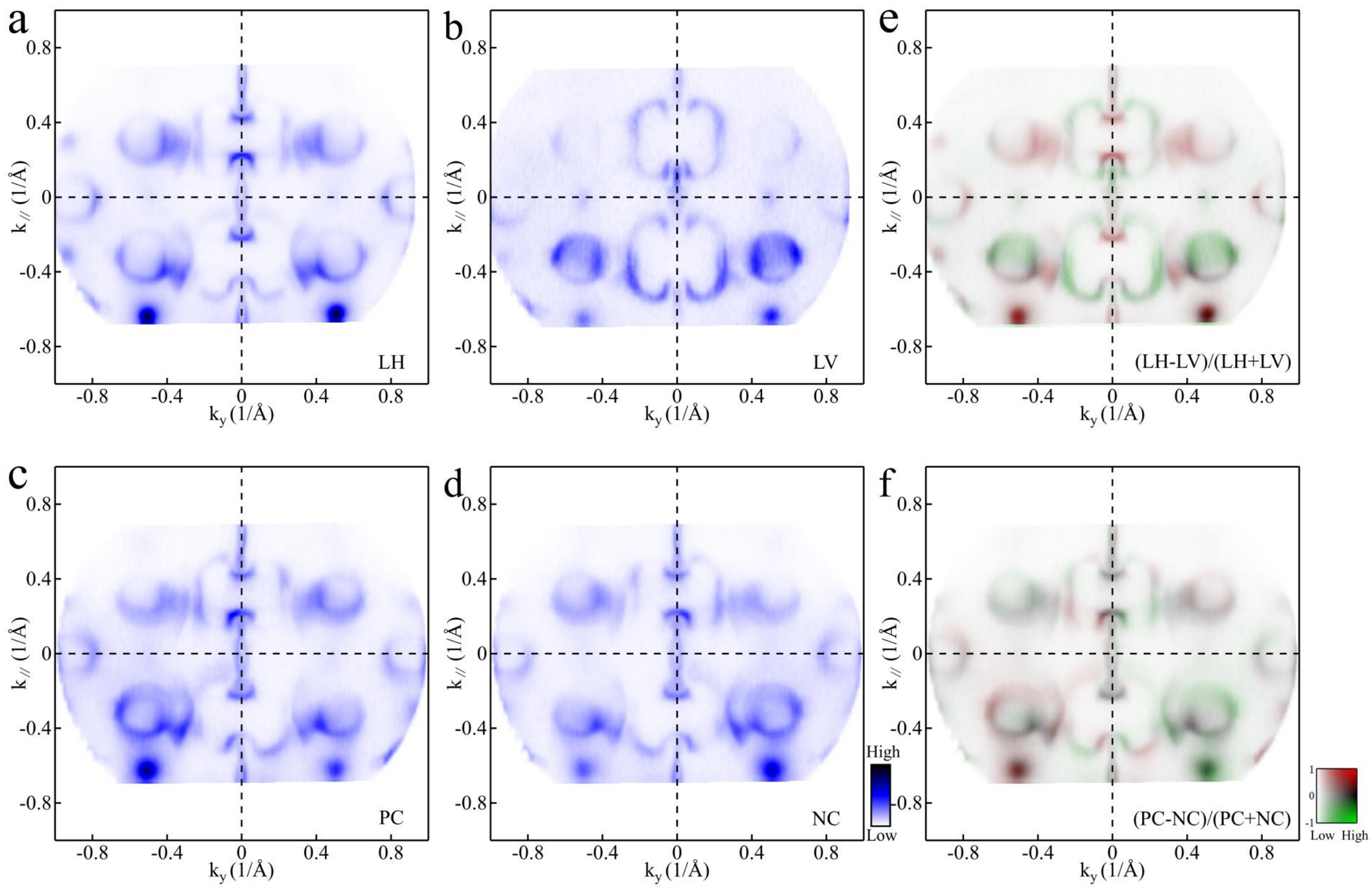

Supplementary Information Figure 3. **Polarization dependent SSs of NdAlSi (103) surface.** (a-d) Fermi surfaces of NdAlSi (103) surface measured on a well defined region at 41 eV photon energy under (a) linear horizontal (LH), (b) linear vertical (LV), (c) positive circular (PC), and (d) negative circular (NC) polarizations. (e) The linear dichroic (LD) Fermi surface obtained by the difference between (a) and (b). (f) The circular dichroic (CD) Fermi surface obtained by the difference between (c) and (d). The spectrum is plotted with the two-dimensional colorscale shown, with the horizontal axis corresponding to intensity and the vertical axis corresponding to LD or CD signal.

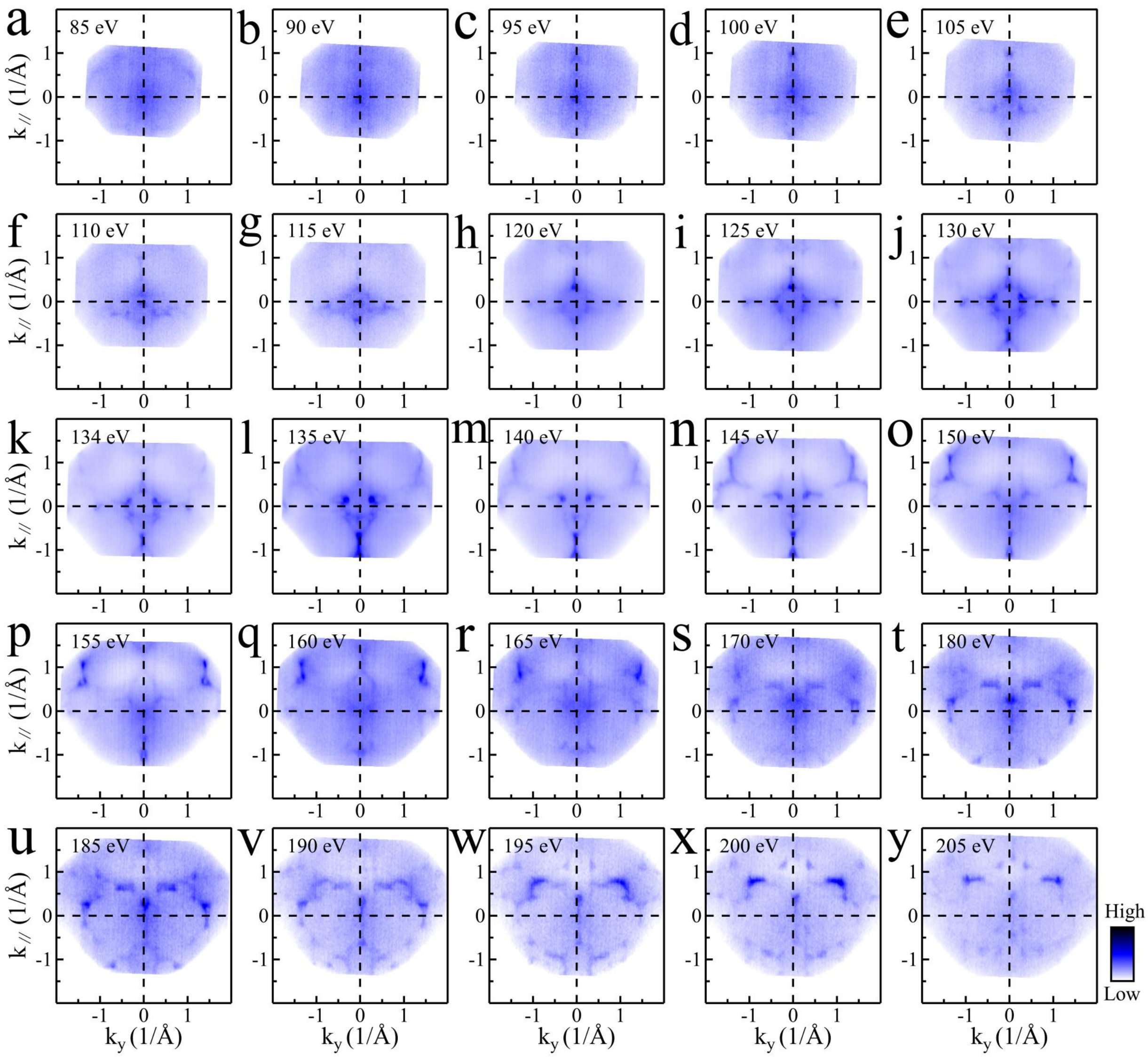

Supplementary Information Figure 4. **Photon energy dependent bulk projected Fermi surface on (103) surface.** (a-y) Bulk Fermi surfaces of NdAlSi (103) surface measured at photon energies from 85 eV to 205 eV.

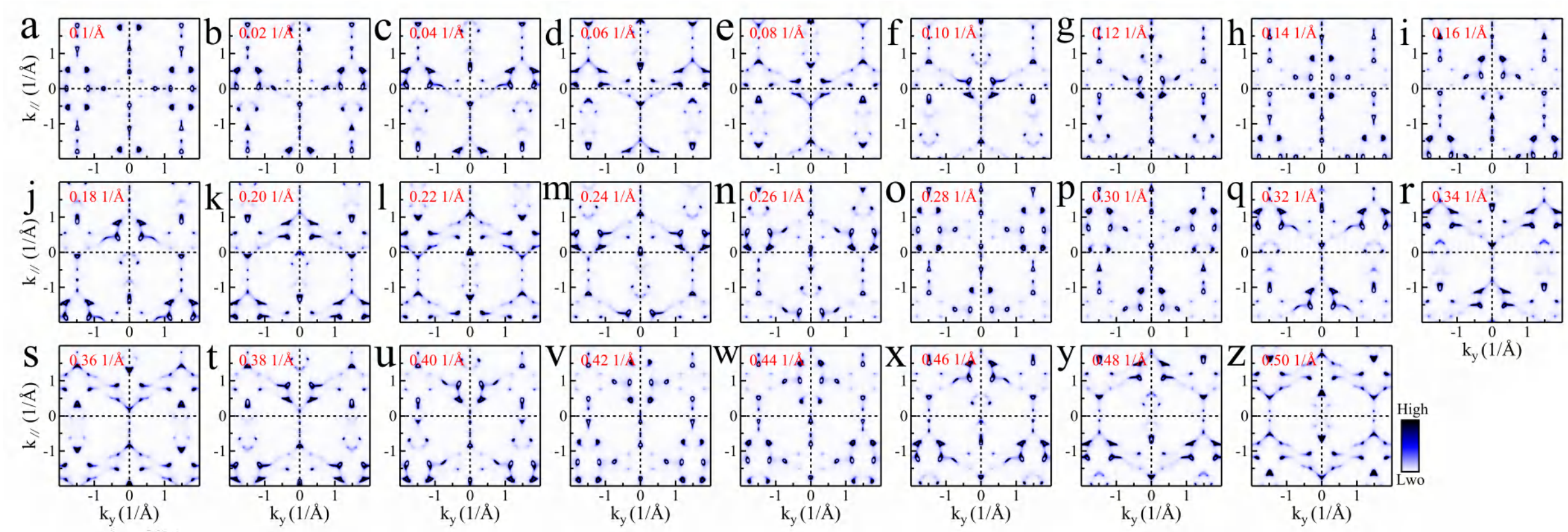

Supplementary Information Figure 5. (a-z) DFT-calculated bulk Fermi surface cross-sections along the [103] momentum direction.

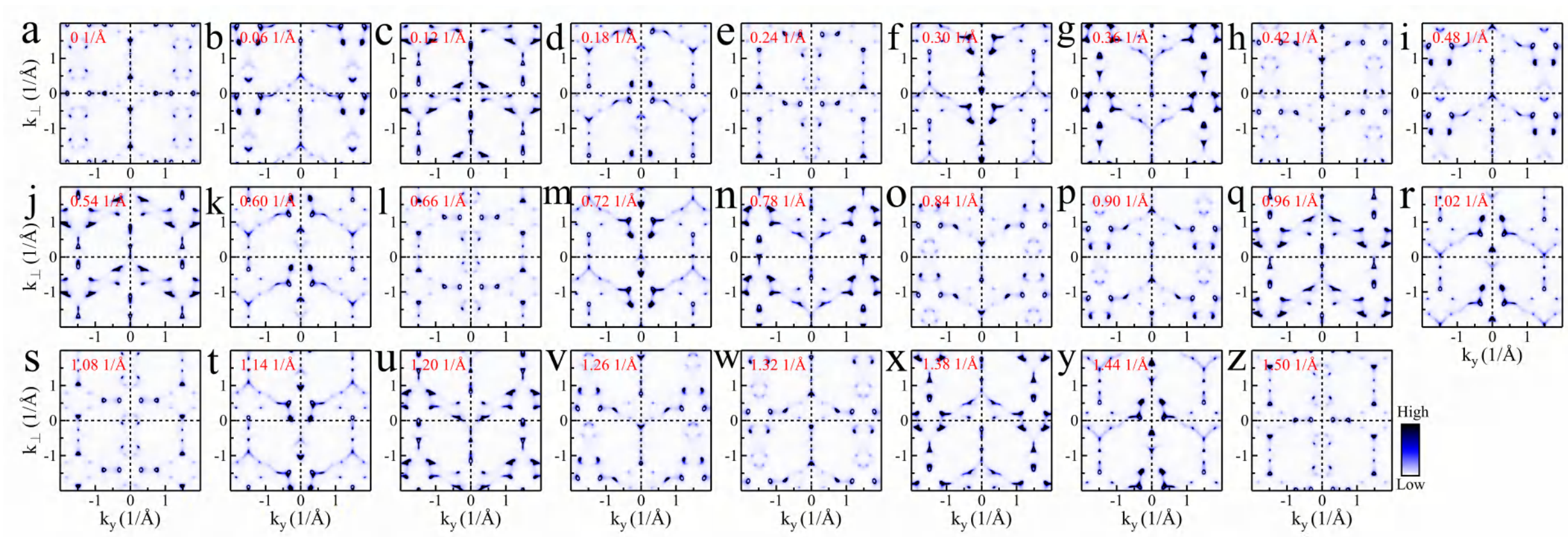

Supplementary Information Figure 6. (a-z) DFT-calculated bulk Fermi surface cross-sections taken along momentum directions perpendicular to [103].

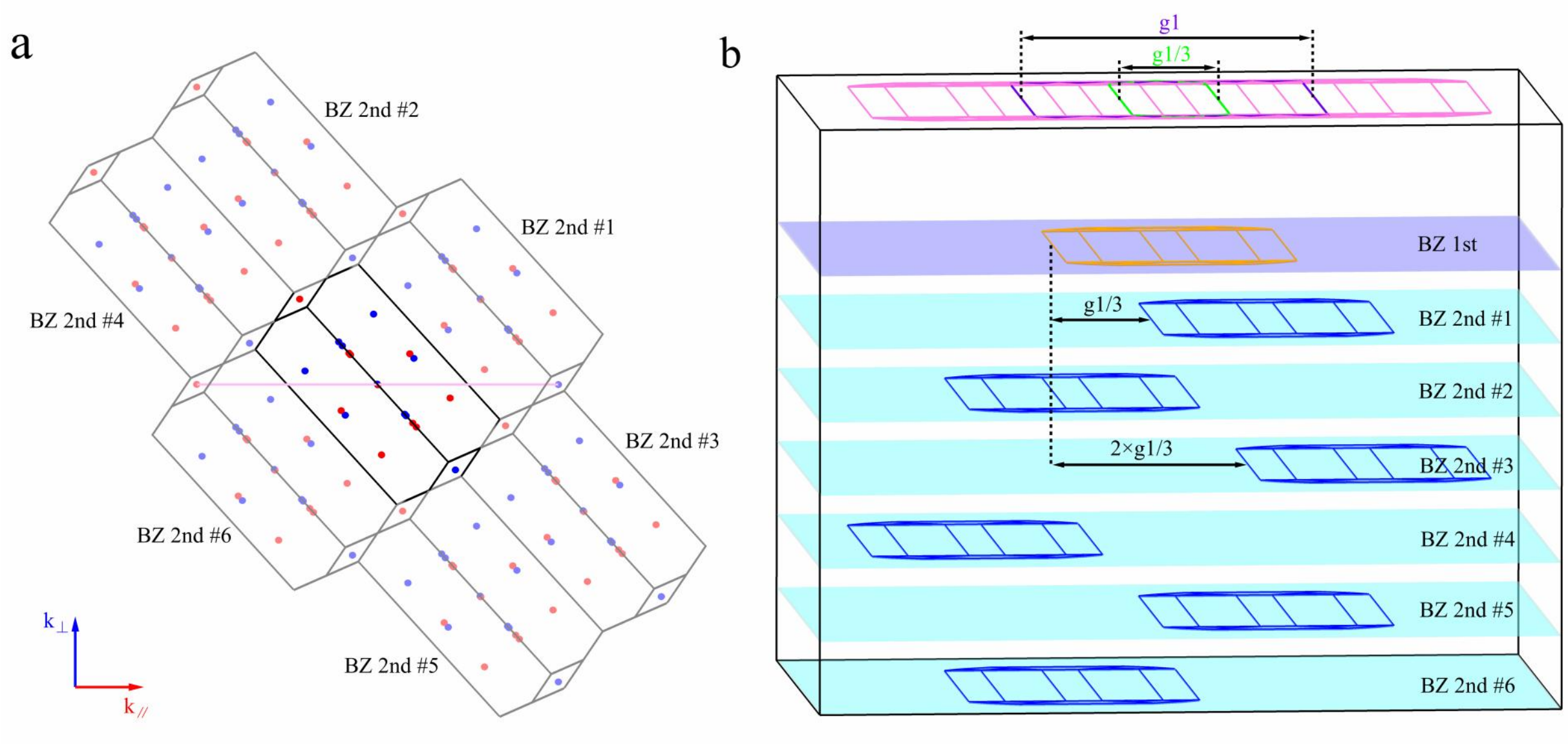

Supplementary Information Figure 7. **Projection of bulk BZ onto (103) surface.** (a) Projection of the first and six second-order bulk BZs onto the (103) plane. Successive second-order BZs project with in-plane displacements due to the incommensurability of bulk and surface reciprocal vectors. (b) Schematic stacking representation of the first (orange) and six second-order BZs (blue), projected onto the (103) surface. The displaced replicas accumulate into a superlattice periodicity that becomes commensurate with the SBZ, thereby restoring BBC on the (103) facet.

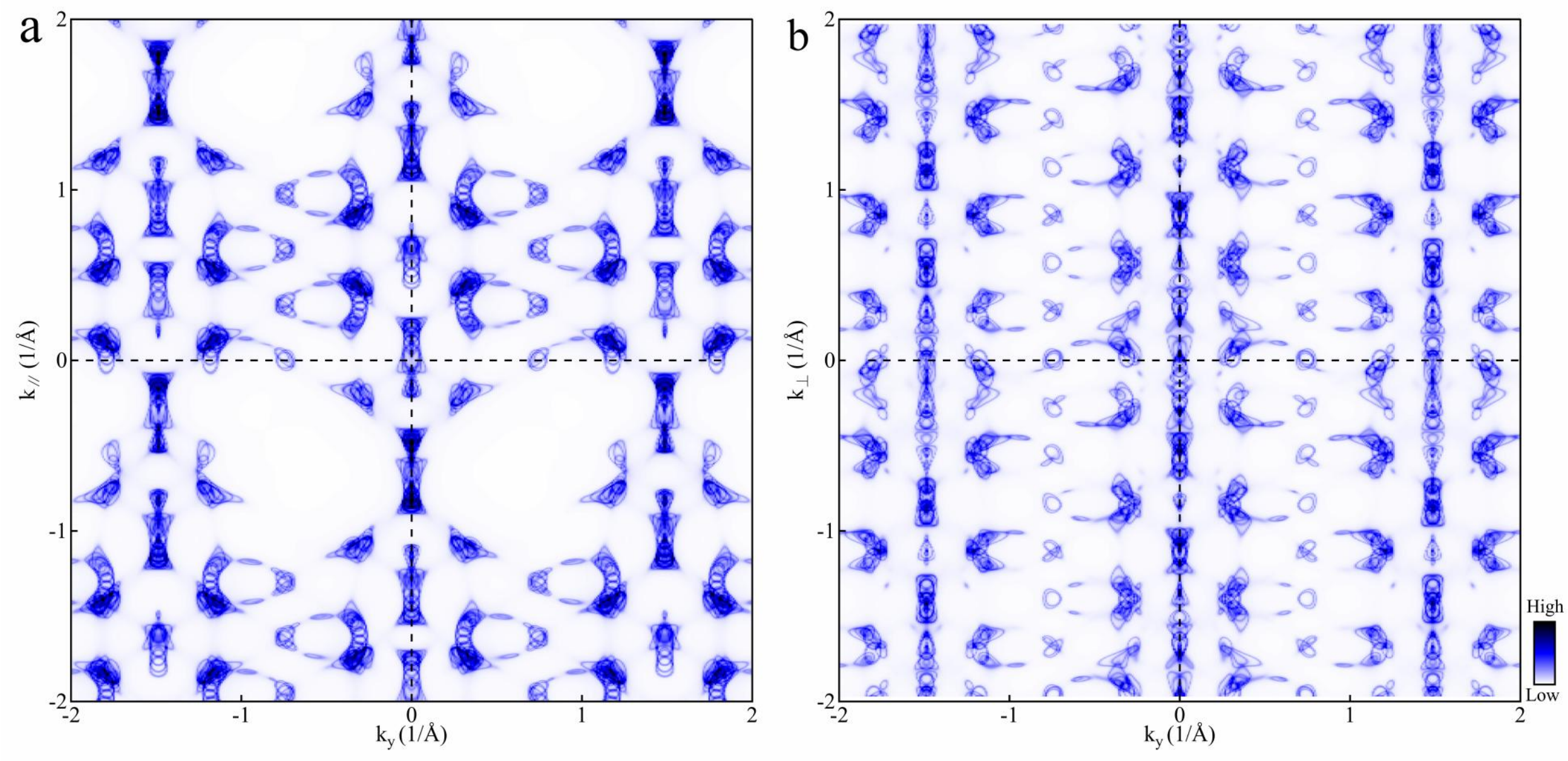

Supplementary Information Figure 8. **Bulk Fermi-surface projections and recovery of surface periodicity on the low-symmetry facet.** (a) Fermi surface obtained by integrating DFT-calculated bulk spectral weight over planes parallel to the (103) surface, with integration range $k_\perp$ = 0-0.5 Å^{-1}. This corresponds to a finite slice of momentum space along the direction perpendicular to the (103) facet. All the integrated data are taken from Supplementary Information Figure 5. (b) Same as (a), but integration is performed over planes perpendicular to the (103) surface, with range $k_{//}$ = 0-1.5 Å^{-1}, using data from Supplementary Information Figure 6. As the integration window increases, contributions from multiple bulk BZs are superimposed, and the apparent periodicity progressively converges toward that of the (103) SBZ.

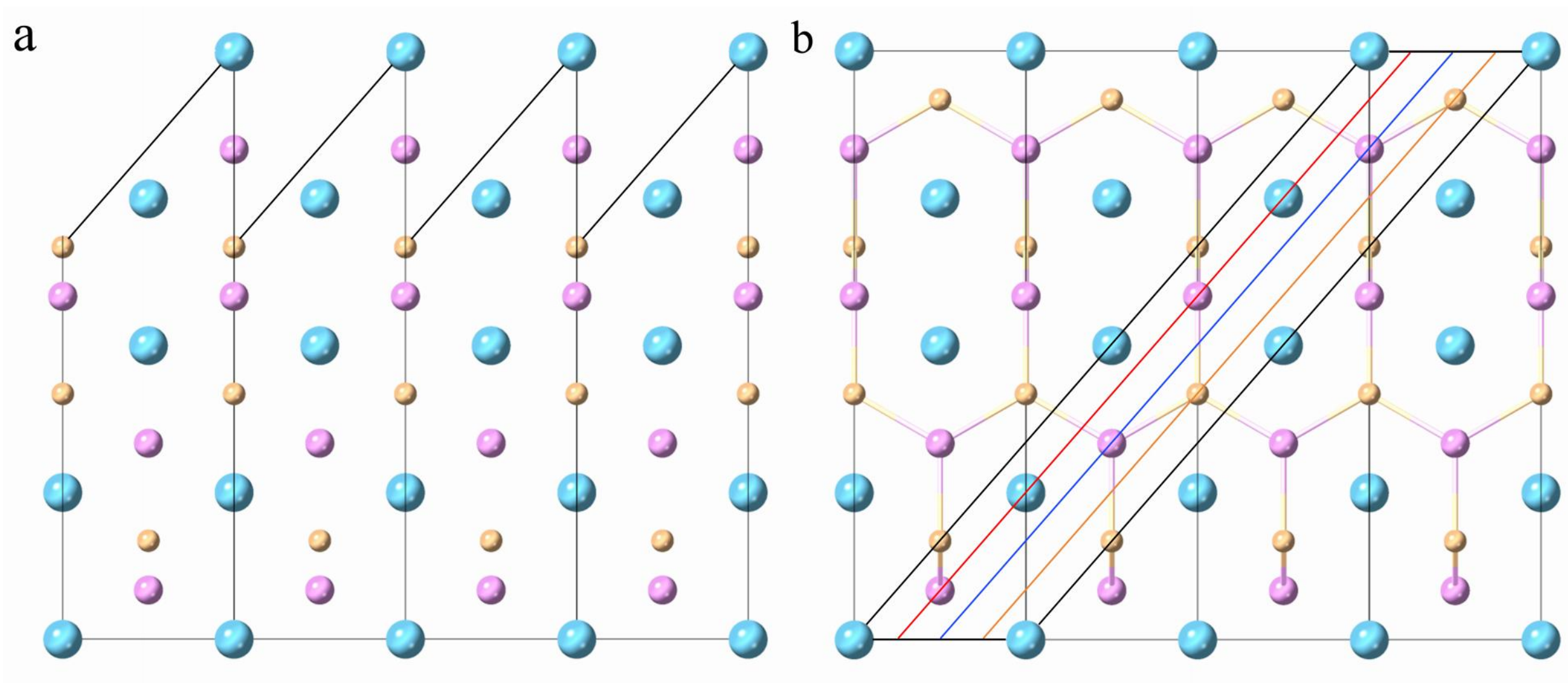

Supplementary Information Figure 9. **Possible cleavage geometries of the (103) surface.** (a) Example of a zigzag-shaped (103) cleavage obtained by cutting along equivalent layers in each primitive unit cell. (b) Cleavage constructed from a three-cell supercell, where four nonequivalent cleavage layers exist, yielding eight distinct terminations. While many cleavage geometries are possible in principle, a consistent description of the SSs requires satisfying the LCM criterion.

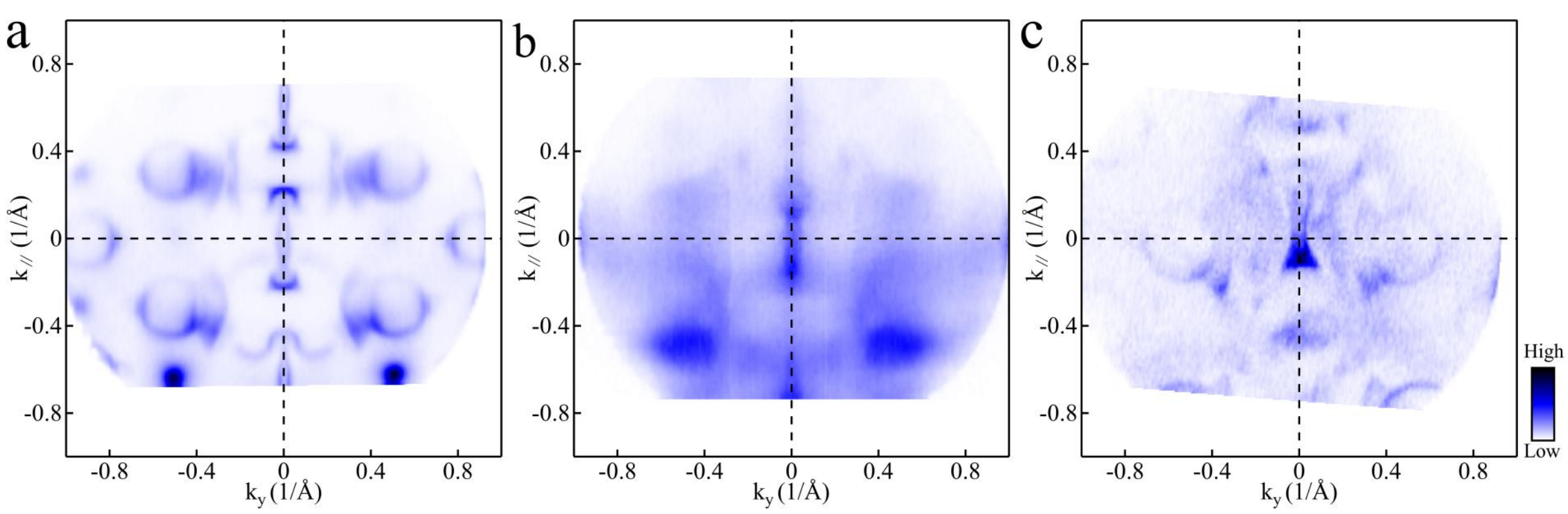

Supplementary Information Figure 10. **Multiple surface-state structures observed on the (103) surface of NdAlSi.** (a) Fermi surface structure corresponding to the termination discussed in the main text. (b-c) Two additional SS structures measured on different regions of the same (103) surface, corresponding to distinct cleavage terminations.

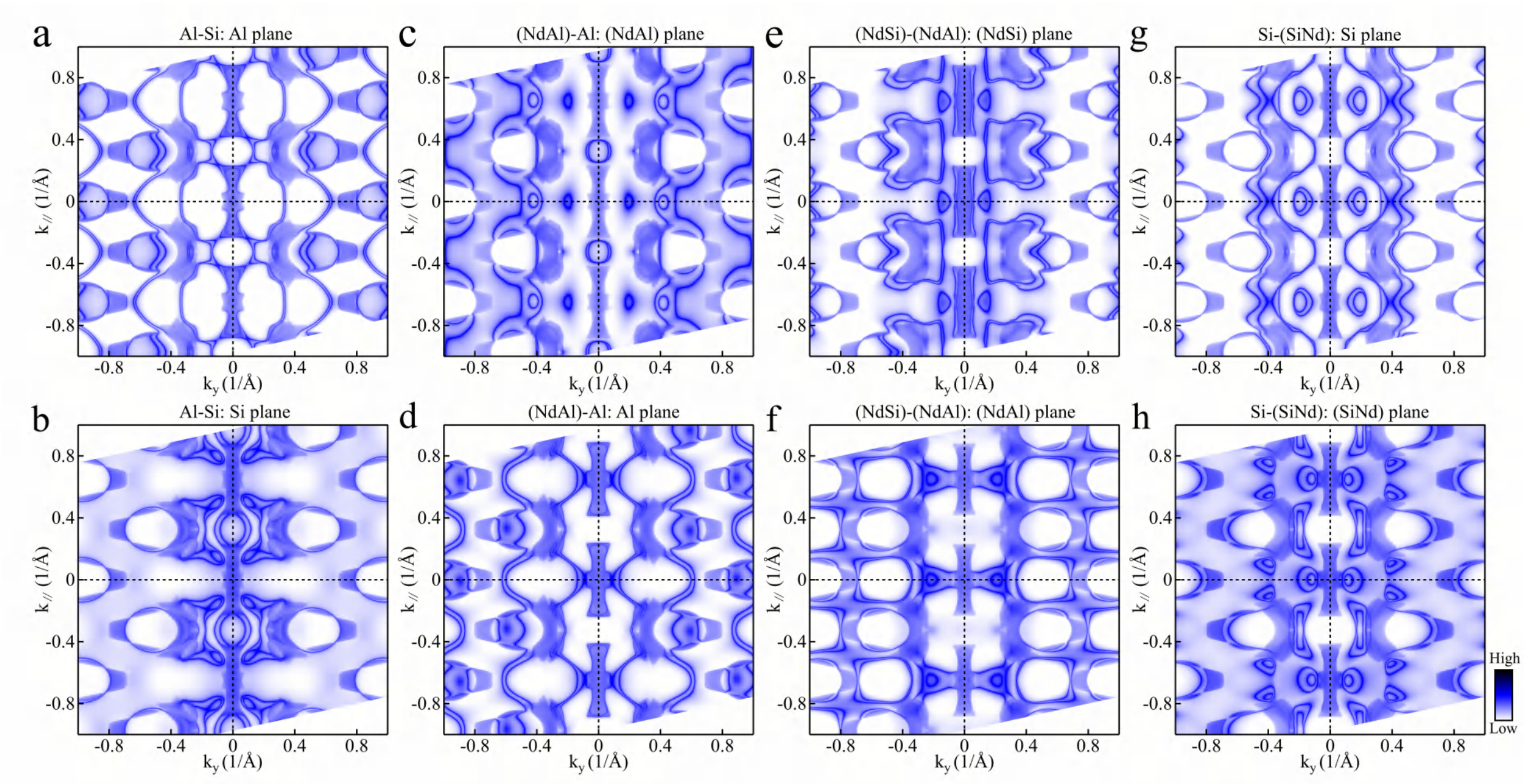

Supplementary Information Figure 11. Surface projected DFT calculations of (103) Fermi surfaces with semi-infinite Green's function for different cleavage terminations: (a) Al terminated surface cleaved at the Al-Si layer, (b) Si terminated surface cleaved at the Al-Si layer, (c) (NdAl) terminated surface cleaved at the (NdAl)-Al layer, (d) Al terminated surface cleaved at the (NdAl)-Al layer, (e) (NdSi) terminated surface cleaved at the (NdSi)-(NdAl) layer, (f) (NdAl) terminated surface cleaved at the (NdSi)-(NdAl) layer, (g) Si terminated surface cleaved at the Si-(SiNd) layer and (h) (SiNd) terminated surface cleaved at the Si-(SiNd) layer. The four cleavage layers defined within the supercell construction shown in Supplementary Information Figure 9b.

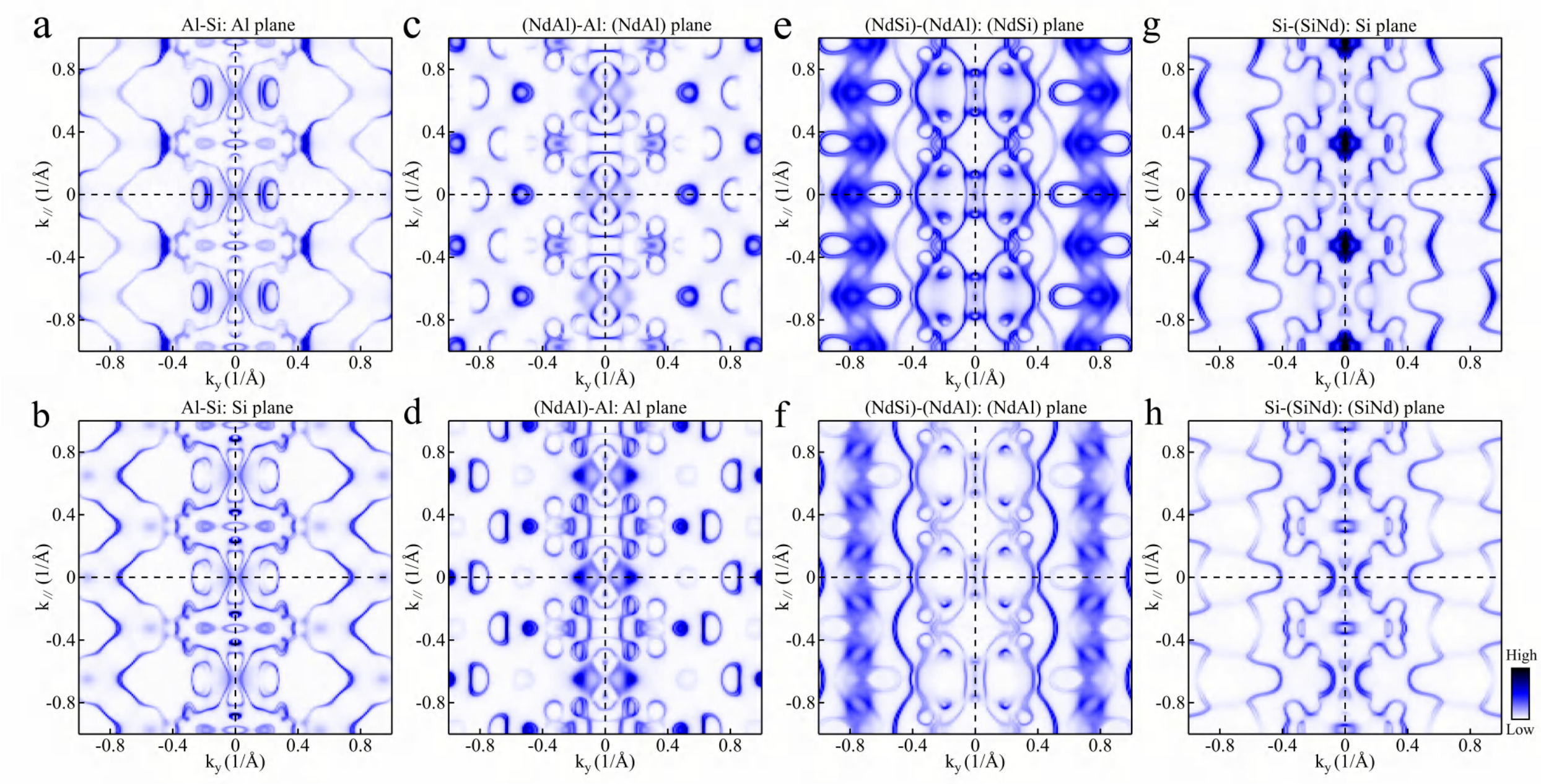

Supplementary Information Figure 12. Fermi surfaces calculated using a six-layer slab for the same cleavage terminations as in Supplementary Information Figure 11.

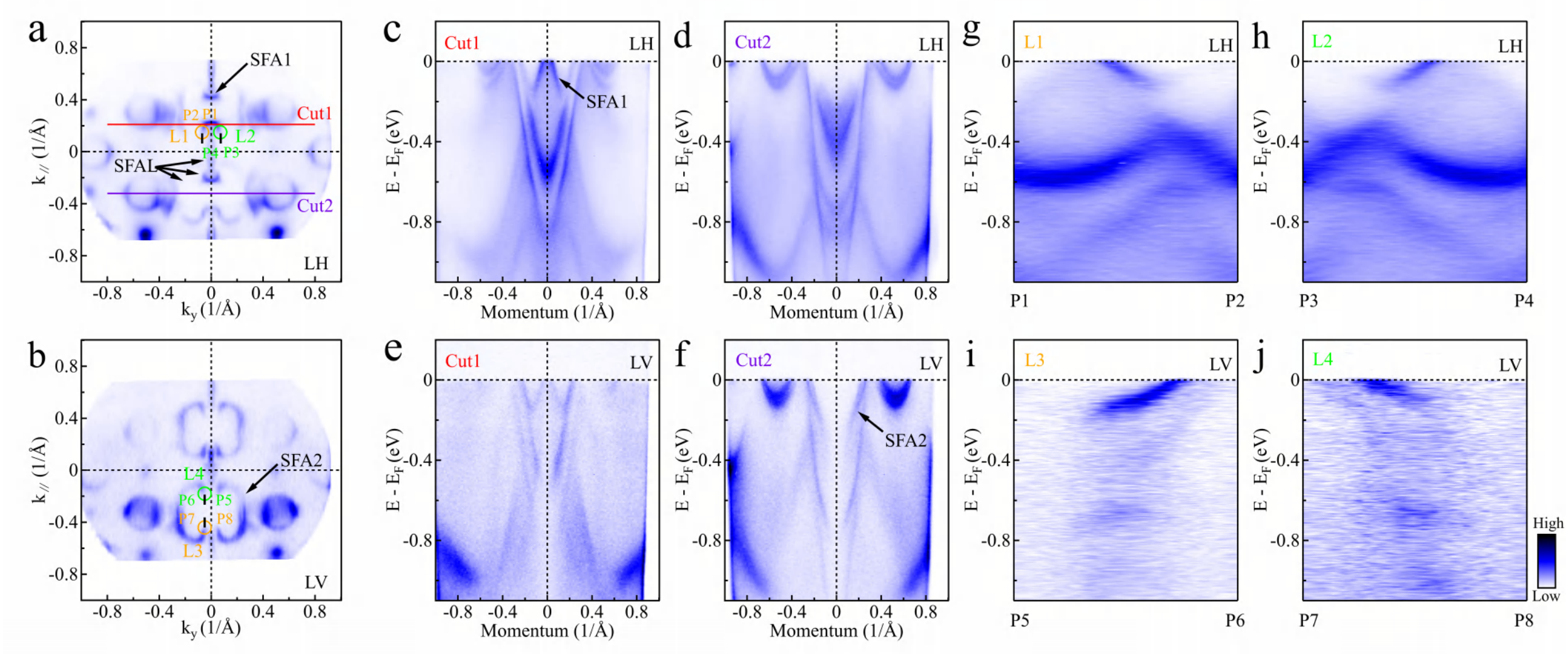

Supplementary Information Figure 13. **Identification of surface Fermi arcs.** (a-b) Fermi surfaces measured with LH (a) and LV (b) polarizations with defined momentum cuts. (c–f) Cuts through arc-like features reveal dispersions consistent with surface Fermi arcs (SFAs). (g-j) Cuts forming closed loops indicate nonzero Chern number and projected bulk charge, consistent with topological origin. On the low-symmetry (103) surface, hybridization between arcs from successive BZs can form closed SFALs marked by black arrows in (a).

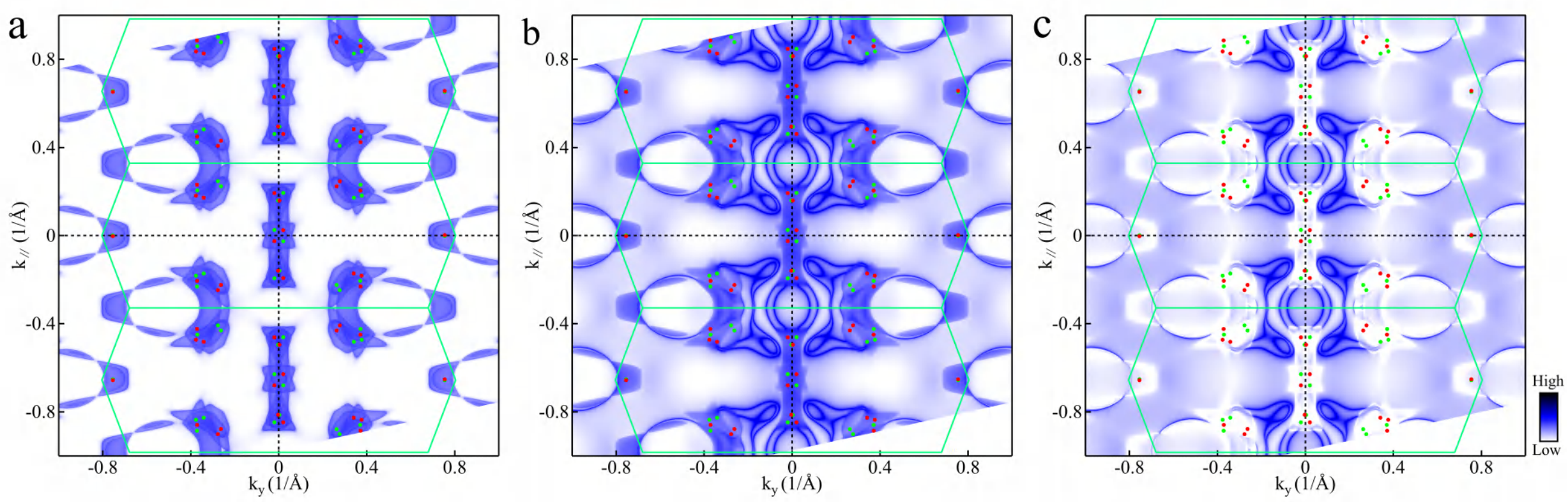

Supplementary Information Figure 14. **Calculated surface spectral functions and projected Weyl points on the (103) surface.** (a) SBPSs obtained from surface Green's function calculations. (b) Total surface spectral function including both SBPSs and true SSs. (c) Pure SSs obtained by subtracting the SBPS contribution in (a) from the total spectrum in (b). Red and green dots indicate the projections of Weyl points from the first-order to third-order bulk BZs onto the (103) SBZ, with red (green) corresponding to chirality +1 (−1). The green lines represent the SBZs.

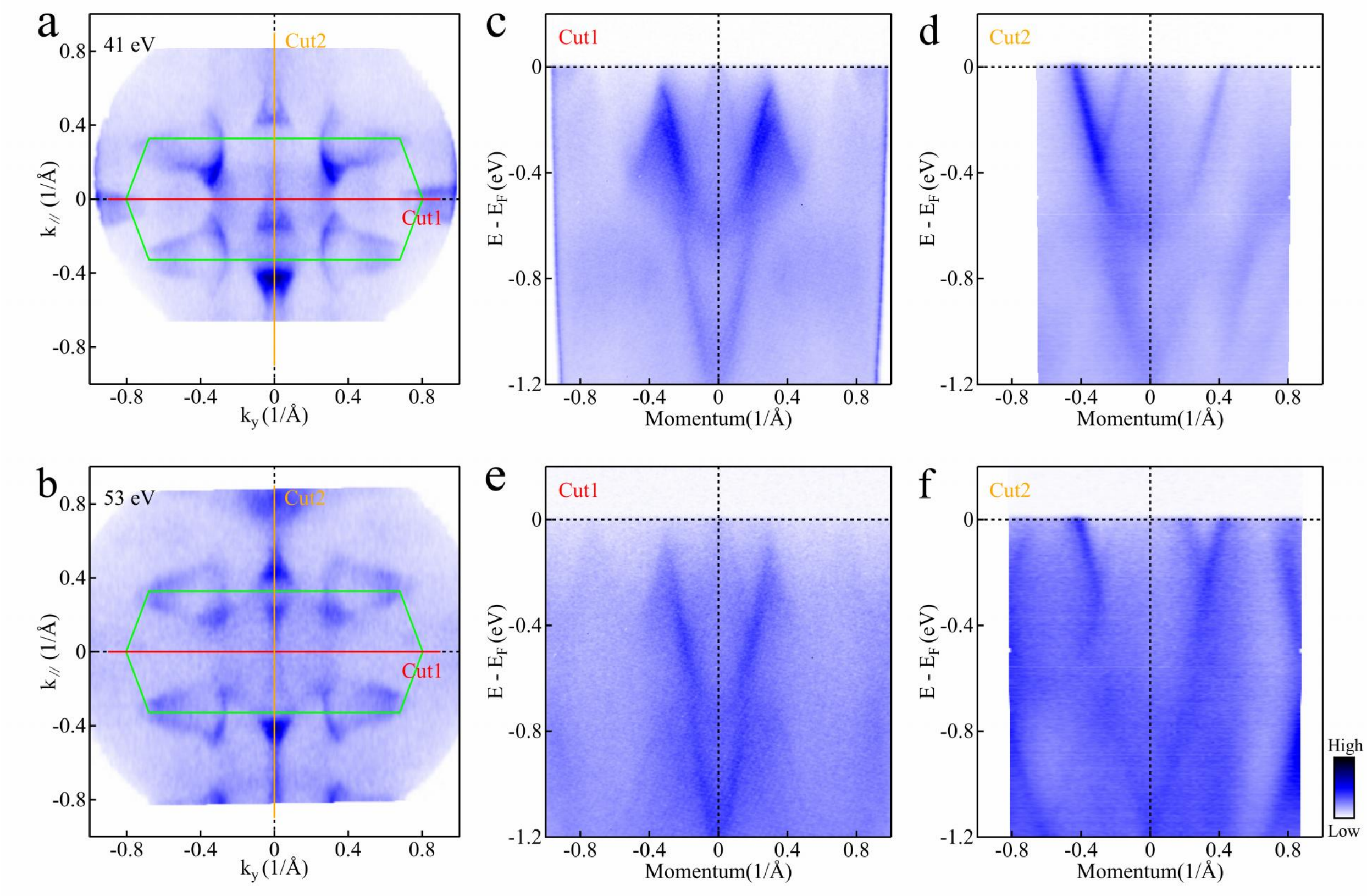

Supplementary Information Figure 15. **Suppression of SSs and emergence of surface-bulk resonance states.** (a-b) Fermi surface maps on well defined surfaces with photon energies of 41 eV (a) and 53 eV (b) after deliberate adsorption of impurities. (c–d) Corresponding band dispersions along high-symmetry cuts Cut1 (red) and Cut2 (orange) measured with photon energy of 41 eV. (e-f) Similar measurements as (c-d) but measured with photon energy of 53 eV.

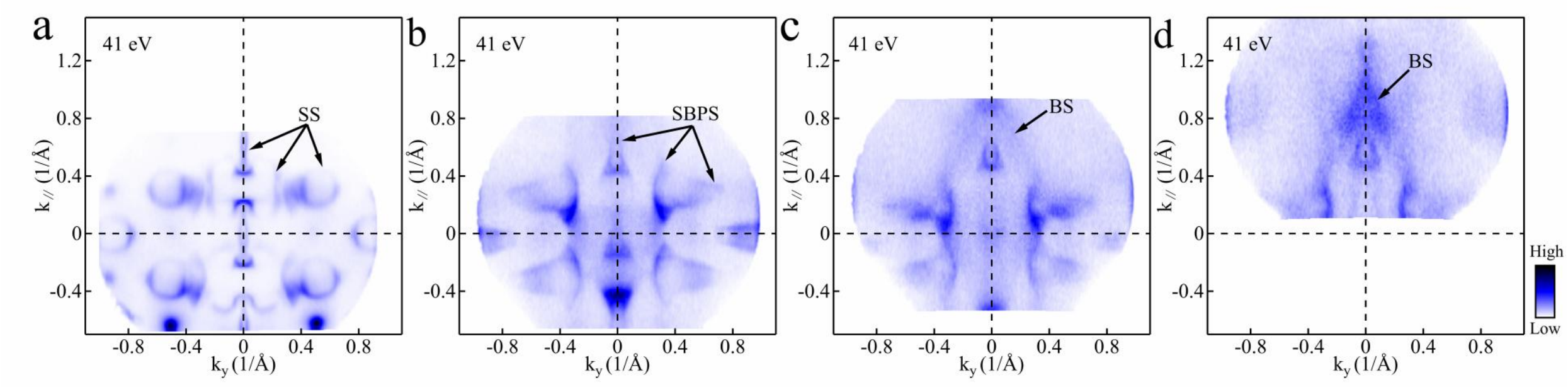

Supplementary Information Figure 16. Comparison of SSs, surface-bulk projected states (SBPSs), and bulk states (BSs) of NdAlSi (103) surface: (a) SSs dominated, (b) SBPSs dominated, (c-d) BSs dominated. All the Fermi surfaces measured with photon energy of 41 eV.

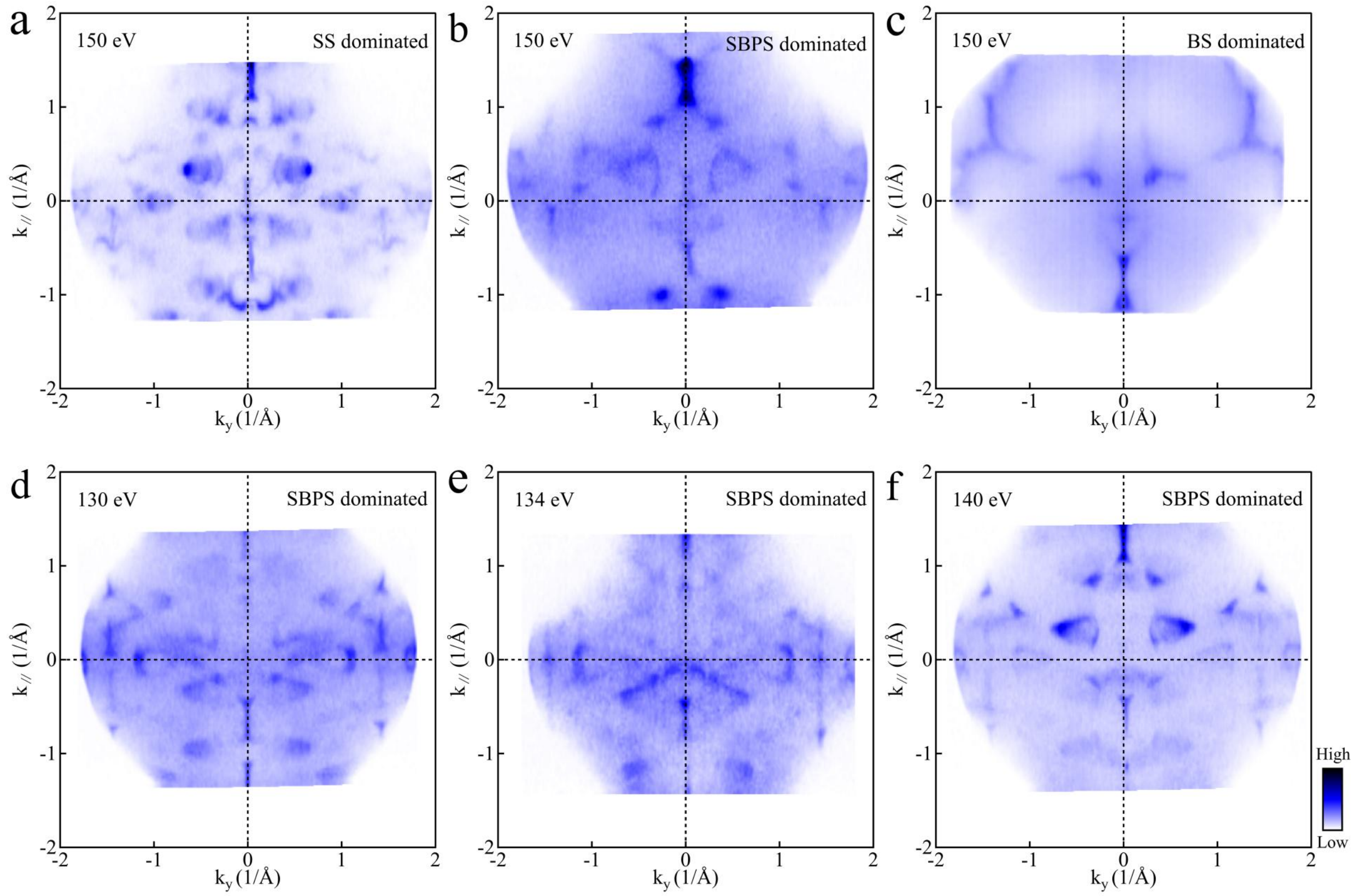

Supplementary Information Figure 17. **Photon energy dependence of Fermi surfaces highlighting SS, BS, SBPS contributions.** (a–c) Fermi surfaces measured at $h\nu = 150$ eV under different surface conditions. (a) On a fresh and flat (103) surface, the spectrum is dominated by sharp SSs. (b) After mild impurity adsorption that introduces surface disorder, coherent SSs are suppressed, allowing SBPSs to dominate. (c) On an uneven surface region, the spectrum is instead dominated by incoherent BSs. (d–f) Fermi surfaces measured at $h\nu = 130$ eV, 134 eV, and 140 eV, respectively, on a disordered flat surface. In all cases, SBPSs dominate the low-energy spectral weight, confirming their robustness across a wide photon-energy range.

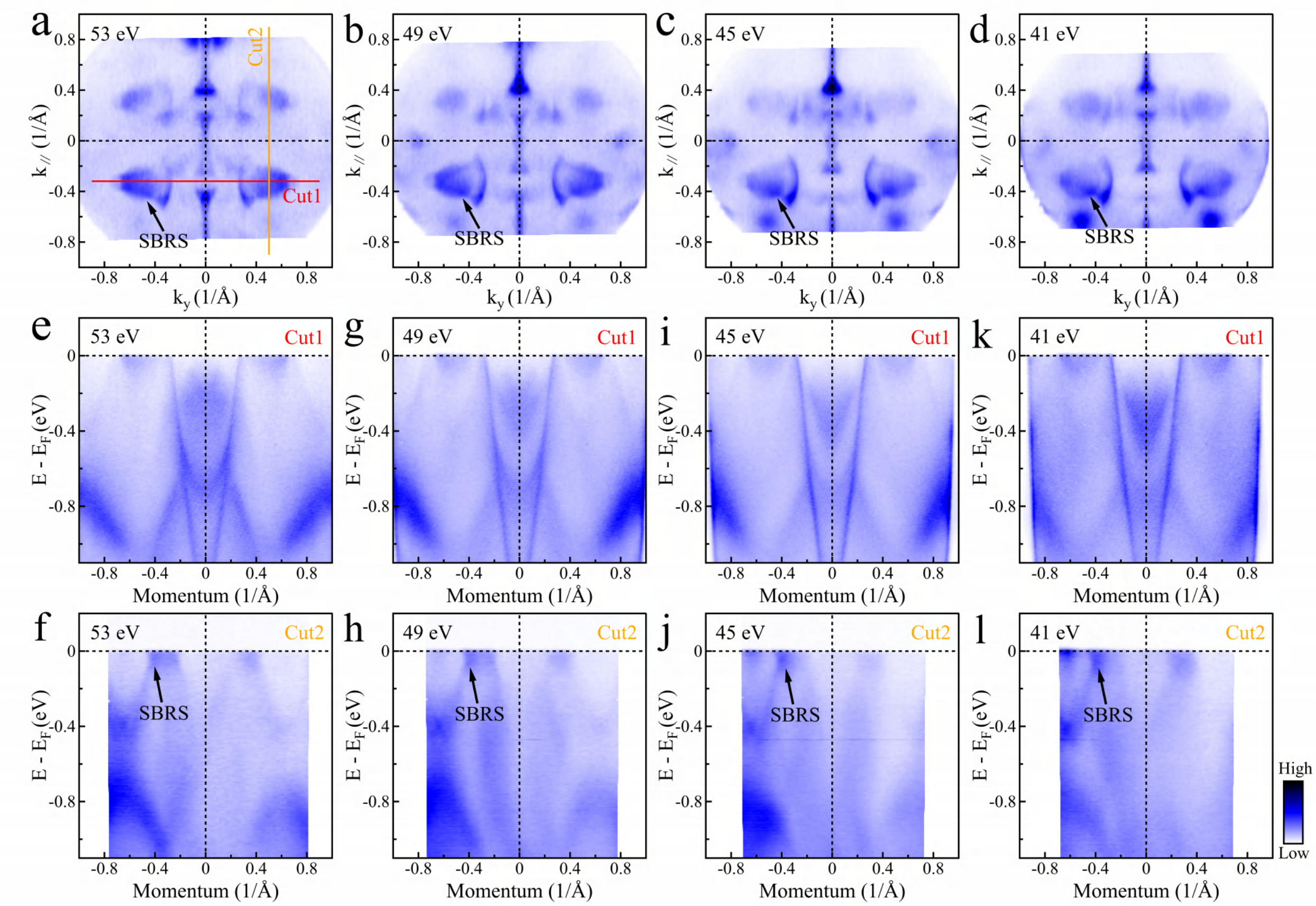

Supplementary Information Figure 18. **Distinguish of SBRS.** (a-d) Fermi surface maps recorded with photon energies of 53, 49, 45, and 41 eV, respectively. The sharp circular contours are attributed to SSs, while the broader triangular features originate from the SBPS. When the SSs overlap in energy and momentum with the SBPS continuum, they hybridize with the bulk Bloch states and evolve into SBRSs, as highlighted by the arrows. (e-l) The corresponding band dispersions taken along the momentum cuts Cut1 [red lines in (a)] (e, g, i, k)and Cut2 [orange lines in (a)] (f, h, j, l) defined in (a).
