# Supplementary material for: Topological Fermi arc interference on low-symmetry Weyl surfaces
Source: Nat Commun. 2026 Aug 13;17:9706. doi: 10.1038/s41467-026-76639-5 (PMC13562659; doi:10.1038/s41467-026-76639-5)
Supplement: Supplementary file 2 [file 41467_2026_76639_MOESM2_ESM.pdf]

nature portfolio

Peer Review File

Topological Fermi arc interference on Low-Symmetry Weyl Surfaces

Corresponding Author: Dr Cong Li

This file contains all reviewer reports in order by version, followed by all author rebuttals in order by version.

Version 0:

Reviewer comments:

Reviewer #1

(Remarks to the Author)
The authors investigate a low symmetry surface of NdAlSi using ARPES and first principles calculations. This material is a Weyl semimetal and the authors investigate the implications of the low symmetry surface on the topological bulk-boundary correspondence. The authors find that the mismatch between bulk and surface Brillouin zones creates phase-shifted replicas that only close after multiple unit cells, three in this particular case. The resulting interference pattern mimics a moire modulation and raises questions about the topology of the system. Through careful and systematic ARPES and DFT analysis the authors find a topological boundary reconstruction that extends the conventional notion of bulk-boundary correspondence. The results raises intriguing possibilities including a new route to generate flat band electronics structures with enhanced correlations in a topological system.

The manuscript is well written and the arguments are clearly presented. The conclusions are straight forward and supported by the combination of theory and experiment. Given this is an atypical surface to investigate, the in-depth supplemental information provides a useful guide and the authors carefully walk the readers through the concepts. They also show how to generalize the concept through a least common multiple criterion that can be applied to other material systems.

Given the novelty, clarity, and potential impact of the work, I recommend the manuscript for publication in Nature Communications. The only change that needs to be made is that they use the acronym SFAL at the bottom of page 6 before it is defined on page 7. With the small change, the manuscript should be published in its current form

Reviewer #2

(Remarks to the Author)
In this manuscript, the authors investigate the electronic structure of the low-symmetry (103) surface of the Weyl semimetal NdAlSi using ARPES and DFT. They report a mismatch between the conventional surface Brillouin zone (SBZ) and the projected bulk Weyl points. To resolve this, they propose a "Least Common Multiple" (LCM) framework, suggesting that the interference of multiple bulk Brillouin zones creates a "momentum-space moiré" pattern. They further claim the observation of Surface Fermi Arc Loops (SFALs).
The experimental data, particularly the preparation of the (103) surface and the high-resolution ARPES data, is of high quality, however, I have a few concerns regarding the theoretical interpretation and the conceptual framing of the results. The authors use strong terminology, e. g. "Moiré Topology", "New Paradigm", to describe what appears to be standard surface reconstruction/folding physics. Furthermore, there is a clear discrepancy between the proposed theory and the experimental data regarding the topological connectivity (loops vs. arcs) that is not adequately resolved.

1. The "Moiré" Terminology: The authors frame the observed periodicity mismatch and reconstruction as "momentum-space moiré topology". This terminology could be potentially misleading. Nowadays, "Moiré physics" (e.g., in twisted bilayers) implies a specific real-space interference leading to large-scale structural modulations and emergent flat bands due to twist or lattice mismatch. In this work, the phenomenon described seems to be a classic case of a surface reconstruction or a supercell effect required to satisfy boundary conditions on a vicinal/high-index surface. The "interference" described is simply the folding of bands due to the new surface potential periodicity.
The authors need to explicitly justify why this should be classified as "Moiré" physics rather than standard "surface reconstruction" or "Umklapp folding." Does this mechanism lead to the localized states or correlation physics typical of Moiré

systems? If not, the terminology should be toned down to avoid misleading readers.

2. Major Discrepancy between Theory and Experiment (SFALs):
A central claim of the paper is the observation of "Closed Fermi-arc loops (SFALs)". However, the authors' DFT calculations based on the LCM framework fail to reproduce these loops, yielding open arcs instead. The manuscript attributes this discrepancy to "zigzag-type reconstructions" or "mixed local terminations" not captured by the slab model.
This is a significant weakness. If the "LCM framework" is the "universal" solution to the bulk-boundary correspondence paradox, why does it fail to predict the most striking topological feature (the closed loops) in this work? The authors cannot claim the framework "quantitatively reproduces" the spectra while simultaneously admitting it fails to capture the topological connectivity (loops vs. open arcs). The authors should either provide a calculation that reproduces the loops (e.g., using the mixed termination they hypothesize) or significantly retreat from the claim that their theoretical framework fully explains the data.

3. The "LCM Criterion" – Is it Trivial?
The authors present the "Least Common Multiple (LCM)" criterion as a new discovery. From a crystallographic perspective, finding the commensurability between a bulk projection vector and a surface reciprocal lattice vector is a standard procedure to determine the surface supercell size. It is mathematically necessary that the projection closes after N unit cells if the slope is rational. I would suggest the authors to clarify: What is physically new about the LCM criterion beyond standard crystallographic projection arithmetic? The novelty seems to lie in its application to Weyl points, but the geometric principle itself appears well-established.

4. Surface vs. Bulk State Distinction:
The identification of surface states relies heavily on comparing "flat" regions (surface states) vs. "uneven/disordered" regions (bulk states). While the "disorder filter" argument is plausible, it is possible that the "new smaller SBZ" is simply a result of a physical surface reconstruction (i.e., atoms moving to new equilibrium positions) rather than just the electronic interference of bulk projections?
Also, the DFT calculations assume a specific ideal termination (Si-Al). Did the authors perform structural relaxation in their DFT? If the atoms physically reconstruct to a 3x1 supercell, then the electronic folding is a trivial consequence of the structural change, not necessarily a "hidden topology." This distinction is crucial.

5. Magnetic Order:
NdAlSi is a magnetic material (Nd moments). The authors state it is non-magnetic at the measurement temperature (paramagnetic) and use a non-magnetic Nd pseudopotential. Even in the paramagnetic phase, local moments and spin fluctuations can be significant, especially for surface states. Given that Weyl physics is sensitive to time-reversal symmetry, can the authors rule out that the "closed loops" are not a result of surface magnetism or surface-induced magnetic ordering, rather than the LCM mechanism?

In summary, this work shows very nice data on a complex system. However, the theoretical packaging ("Moiré") feels forced, and the failure of the proposed model to reproduce the key topological feature (closed loops) suggests the physical picture is incomplete. Before further consideration, I suggest that the authors provide a more rigorous reconciliation of the discrepancy between the theoretical model and the experimental results; and use a precise physical definition of the observed phenomena, avoiding overhyped terminology.

Reviewer #3

(Remarks to the Author)
In this study, C. Li et al. have used synchrotron-based ARPES and DFT-based surface Green's function methods to study the topological surface states and Fermi arcs on the low-symmetry/high-Miller-index (103) surface of NdAlSi, an inversion-breaking Weyl semimetal, above its magnetic phase transition. By using the monoclinic supercell in Fig.S9(b) with the whole (103) surface cell as the side surface, the calculated spectral functions on the semi-infinite (103) surface explain well the observed ARPES data for both flat region with Si termination in Fig.1(f) and disordered region with multiple terminations in Fig.1(e). The authors did a detailed analysis of bulk-to-surface Brillouin zone (BZ) projection and a systematic comparison between ARPES and topological surface state calculations in Fig.2, 3 and 4.

However, the manuscript has many overclaims and even misconception, which make it unacceptable for the high standard of Nat. Comm. Here are the main comments.

The abstract claims that "bulk and surface periodicities are inherently mismatched" by showing the partial (103) surface region in Fig.1(a) for a zigzag pattern with a single bulk unit cell. But it is well known that the high Miller index surfaces often span multiple bulk unit cells. For example, for groups of FCC high Miller index surfaces, please see Fig.15 Pt(310) in Santos et al. Results in Surfaces and Interfaces 3, 10006 (2021). The proper choice of the NdAlSi (103) surface unit cell is not Fig1.(a) but actually Fig.S9(b). Such a surface supercell can be obtained more intuitively by the real space axis intercept method, which is equivalent to the least-common-multiple (LCM) method in reciprocal space as the authors detailed in Supplementary Materials. The (103) surface periodicity still has the same underlying bulk periodicity as any other surface orientations. The "new smaller SBZ" (green in Fig.1(b-c)) comes naturally when the proper (103) surface unit cell is chosen in Fig.S9(b), which is more intuitive from the real space intercept method. Thus, there is no inherent mismatch between the bulk and surface periodicities. The proper (103) surface unit cell in Fig.S9(b) should be included in the main text Fig.1.

The manuscript also claims in the title, abstract and other places of the so-called “hidden moire in reciprocal space”, “translational commensurability is lost” and “bulk-surface incommensurability”. But there are no extra competing interactions beside the bulk projection to impose any additional long periodicity on the surface. There is no moire-like flat-band in the NdAlSi (103) surface spectral functions as shown in data and figures. The SBZ is perfectly commensurate with the bulk BZ when not a single but the whole 3D reciprocal space (including the Weyl points) is properly projected into the 2D sheet for SBZ, which is equivalent to the semi-infinite surface Green’s function. As the authors also noted on p.3 that “the mismatch is not a breakdown of BBC but a natural outcome of incomplete bulk projection”. Thus, there is no lost translational commensurability between bulk and surface, but the need of proper and complete bulk-to-surface projection.

The need of multiple bulk BZ projection to SBZ to fully account for the topological surface states is not uncommon. Even for the high-symmetry rock-salt (001) surface (please see Fig.4 in Kuroda et al, PRL 120, 086402 (2018)), where the projection of two bulk BZ is needed for the band inversion at the X points to fully explain the gapped surface Dirac cones at the M point of the (001) SBZ.

Thus, given that these key claims in the manuscript are exaggerated, the low-symmetry/high-index surface of inversion-breaking Weyl semimetal such as TaAs have already been studied by Zheng et al in Ref.19, the current manuscript does not meet the high standard of Nat. Comm.

Version 1:

Reviewer comments:

Reviewer #1

(Remarks to the Author)
After reviewing the full set of reviewer comments, the authors’ rebuttal, and the revised manuscript, my overall assessment is that the revision improves the paper in meaningful ways but does not fully resolve the concerns raised during peer review. The manuscript remains interesting and potentially impactful, especially because it combines technically strong ARPES measurements with theory to study an unusual low-symmetry surface of a Weyl semimetal. The revised version is stronger than the original in that it more clearly distinguishes between what is firmly supported by the data and what remains more interpretive. At the same time, several of the most important criticisms from Reviewers 2 and 3 were only partially addressed, and in some cases the rebuttal is more careful and persuasive than the revised manuscript itself.

To the authors’ credit, they responded constructively to the criticism about the discrepancy between theory and experiment regarding the claimed closed Fermi-arc loops. The revised manuscript now makes a clearer distinction between the LCM framework as a way to resolve the commensuration and periodicity problem on a low-symmetry surface, and the separate question of whether the detailed connectivity takes the form of open arcs or closed loops. The paper now acknowledges that the LCM-consistent slab calculation reproduces the reconstructed periodicity and dominant replica structure, while the loop-like connectivity likely depends on additional microscopic surface conditions such as termination mixing, reconstruction, disorder, or enhanced hybridization. That clarification strengthens the paper and directly addresses one of the most serious concerns of the Reviewers.

However, the revisions create a problem with internal consistency. The manuscript still contains language, particularly in the title and abstract, that overstates the level of agreement between theory and experiment. The abstract continues to suggest that the calculations quantitatively reproduce the observed spectra, even though the body of the paper now admits that the most visually striking feature, namely the loop-like connectivity, is not reproduced by the idealized slab model. That tension is insufficiently resolved. More broadly, both Reviewers 2 and 3 objected to the strongest conceptual language. The rebuttal offers a thoughtful defense of “momentum-space moiré” as a broader mathematical description of emergent modulation in reciprocal space, but the revised manuscript still retains much of the same expansive framing, including the title “Hidden Moiré Topology,” references to a “new paradigm,” and suggestions of flat-band engineering.

Reviewer 2’s concerns were therefore mostly fair, especially regarding the mismatch between the scope of the theory and the way the results are advertised. Reviewer 3 also raised some legitimate points, particularly about the risk of overstating the novelty of the LCM construction itself, which the authors appropriately softened by clarifying that the novelty lies in its topological application rather than in new crystallographic mathematics. However, Reviewer 3’s implication that the main phenomenon is merely a routine supercell issue or that the work lacks sufficient novelty altogether seems overly critical. Even if one prefers a more conventional crystallographic description, the data do support a real low-symmetry surface projection problem and a meaningful LCM-guided resolution of the periodicity mismatch.

The manuscript revision is improved, scientifically stronger, and is still suitable for Nature Communications but the revisions create internal inconsistencies and do not fully resolve the criticism of Reviews 2 and 3. It is recommended that the manuscript be revised to address these issues prior to publication.

Reviewer #3

(Remarks to the Author)
In the rebuttal letter, the authors have tried mostly to argue against the concerns of Reviewer 2 and 3 on packaging

overclaims and exaggerated statements with the usage of “hidden moire”, “bulk and surface periodicities are inherently mismatched”, “translational commensurability is lost” and “bulk-surface incommensurability”, without modifying these problematic statements in the manuscript. After carefully reading the lengthy arguments that the authors have presented, this reviewer find these arguments are not convincing and the revised manuscript is not suitable for publication.

The authors have acknowledged the equivalence of their LCM and the straightforward intercept-based method that is widely accepted in surface science and crystallography communities to generate the proper surface unit cell for low-symmetry or high Miller index surfaces. It is also well-known that the surface unit cells for high Miller index surfaces, like the NdAlSi(103) here, usually span serval primitive bulk unit cells in real space, thus, in reciprocal space, the surface Brillouin zones (SBZ) need a complete projection from 3D to 2D, not just a section of the first bulk BZ. The authors have also agreed that the key finding of the good agreement between the ARPES and surface Green’s function calculation relies on the proper (103) surface unit cell and the inherent complete bulk-to-surface projection from 3D to 2D in the surface Green’s function calculation.

Both Reviewer 2 and 3 pointed out that such proper surface unit cell and complete bulk-to-surface projection should be the default solution to consider for high Miller index surface. The reduced SBZ compared to bulk BZ is not from the so-called “hidden moire” but the results of normal practice in surface science and crystallography. The need of complete bulk-to-surface projection is also common in surface band structure calculations using surface Green’s function. Again one such example is the CeX (X=P, As, Sb and Bi) surface states (please see Fig.4 in Kuroda et al, PRL 120, 086402 (2018)). Even for the high-symmetry rock-salt (001) surface, where the projection of two bulk BZ is needed for the band inversion at the X points to fully explain the gapped surface Dirac cones at the M point of the (001) SBZ. However, the authors have ignored such example in the rebuttal letter.

If such “hidden moire” and similar overclaims are accepted for publication with just using the default practice to generate proper surface unit cell and complete bulk-to-surface projection, then should all high Miller surfaces of many other materials be called “hidden moire” from now on? Despite there is no competing interaction being introduced in the single phase of crystal structure for moire physics, but just BZ projection and folding.

Version 2:

Reviewer comments:

Reviewer #1

(Remarks to the Author)
I have reviewed the manuscript, the authors’ rebuttal, and the revised Supplementary Information. In my view, the authors have made meaningful and constructive revisions. The manuscript is now substantially better aligned with the evidence than the prior version, and most of the concerns raised by all reviewers have been addressed at the manuscript level rather than only in the rebuttal.

The strengths of the manuscript remain significant. The work reports technically demanding ARPES measurements on the low-symmetry NdAlSi(103) surface, a surface that is not straightforward to prepare or interpret. The authors describe a careful pre-orientation, pre-cutting, and in situ cleavage procedure, and the ARPES measurements were performed with good energy/angular resolution at two synchrotron beamlines. The central experimental observation that flat, well-defined regions show sharp surface-state features while uneven/disordered regions suppress these states and reveal more bulk-like spectral weight is compelling and provides a useful experimental basis for separating surface, bulk-projected, and resonance-like contributions. The photon-energy-dependent measurements and comparison with DFT further support the interpretation that bulk projections shift systematically with k_perp, which is essential to the authors’ LCM-based commensuration argument.

The most important improvement is the reframing of the theory-experiment comparison. The authors have changed the title to the more appropriate “Topological Fermi arc interference on Low-Symmetry Weyl Surfaces” and have rewritten the abstract to emphasize Fermi-arc interference, reconstructed periodicity, and dominant replica structure rather than the earlier, more expansive “hidden moiré topology” framing. They now explicitly distinguish the commensuration/periodicity problem, which is reasonably well captured by the LCM-guided calculations, from the detailed arc connectivity, including the loop-like features, which remain dependent on microscopic surface conditions not fully captured by the idealized slab model. This directly addresses the main concern from the previous review: the manuscript no longer implies that the calculations fully reproduce the most visually striking loop-like connectivity. The discussion now states more carefully that the calculations capture the reconstructed periodicity and dominant replica structure, while the detailed loop-like connectivity depends on termination mixing, reconstruction/disorder, and surface-bulk hybridization.

The authors also responded meaningfully to the concern that the LCM construction should not be advertised as new crystallographic mathematics. In the rebuttal, they explicitly state that the title, abstract, and conclusion were revised, that “hidden moiré” language was removed or softened, and that the LCM criterion is equivalent to standard intercept-based surface-cell construction. The Supplementary Information now gives a clear mathematical derivation of the LCM prescription and applies it to the NdAlSi(103) case, which justifies the three-cell surface repeat unit. It is reasonable to present the result not as a replacement for standard surface crystallography, but as an operational framework for making the projection problem explicit in the context of ARPES and Weyl surface states.

There are still weaknesses. The evidence for closed surface Fermi-arc loops remains suggestive rather than definitive. The Supplementary Information presents ARPES cuts and Green's-function calculations supporting an assignment to topological surface Fermi arcs, but it also acknowledges that the detailed experimental connectivity is not fully captured by the idealized calculations. The proposed formation of SFALs through hybridization of arcs from different projected bulk zones is plausible, but it remains an interpretation rather than a quantitatively demonstrated microscopic mechanism. I am satisfied that the main text now frames this distinction appropriately, but I would encourage the authors to keep the language around "closed loops" and "generalized BBC" cautious, especially in the Supplementary Information.

There is also an apparent figure-label inconsistency in the Supplementary Information: it refers to the surface-state Fermi surface in Fig. 1e and the suppression of surface states in Fig. 1f, whereas the main text and Fig. 1 caption identify Fig. 1e as the uneven/disordered region and Fig. 1f as the flat, well-defined surface-state region. This should be fixed, but it does not affect the scientific conclusion.

Overall, I find that the authors have addressed the major review concerns in a meaningful way. The manuscript is still somewhat ambitious in its conceptual framing, and the SFAL interpretation is not as firmly established as the periodicity/commensuration result. However, the revised paper now makes that distinction clear. The ARPES data are strong, the low-symmetry surface problem is interesting, and the LCM-guided projection framework is useful when presented with the current level of caution. I therefore recommend the manuscript for publication in Nature Communications after minor textual corrections.

Reviewer #3

(Remarks to the Author)
The revised manuscript has been substantially improved by removing the overclaims and exaggerated statements, such as "hidden moire", "bulk and surface periodicities are inherently mismatched", "translational commensurability is lost" and "bulk-surface incommensurability". The rewriting of key parts of the manuscript (title, abstract and main text) with the more direct and physically transparent way has been well done and presents a much clear picture of the study to be appreciated by the community.

The detailed analysis in both the experimental ARPES and DFT calculations on the low-indexed NdAlSi (103) reveal the inherent need for a complete 3D to 2D projection (multiple bulk BZ to surface BZ) in order to better understand Weyl Fermi arcs and connectivity reconstruction. The LCM closure is a nice addition to the standard intercept-based surface-cell construction method.

With these substantial improvements, the manuscript is now suitable for publication in Nat. Comm.

Open Access This Peer Review File is licensed under a Creative Commons Attribution 4.0 International License, which permits use, sharing, adaptation, distribution and reproduction in any medium or format, as long as you give appropriate credit to the original author(s) and the source, provide a link to the Creative Commons license, and indicate if changes were

made.
In cases where reviewers are anonymous, credit should be given to 'Anonymous Referee' and the source.
The images or other third party material in this Peer Review File are included in the article's Creative Commons license, unless indicated otherwise in a credit line to the material. If material is not included in the article's Creative Commons license and your intended use is not permitted by statutory regulation or exceeds the permitted use, you will need to obtain permission directly from the copyright holder.
To view a copy of this license, visit https://creativecommons.org/licenses/by/4.0/

Response to Reviewer's Comments

Reviewer #1 (Remarks to the Author):

The authors investigate a low symmetry surface of NdAlSi using ARPES and first principles calculations. This material is a Weyl semimetal and the authors investigate the implications of the low symmetry surface on the topological bulk-boundary correspondence. The authors find that the mismatch between bulk and surface Brillouin zones creates phase-shifted replicas that only close after multiple unit cells, three in this particular case. The resulting interference pattern mimics a moire modulation and raises questions about the topology of the system. Through careful and systematic ARPES and DFT analysis the authors find a topological boundary reconstruction that extends the conventional notion of bulk-boundary correspondence. The results raises intriguing possibilities including a new route to generate flat band electronics structures with enhanced correlations in a topological system.

The manuscript is well written and the arguments are clearly presented. The conclusions are straight forward and supported by the combination of theory and experiment. Given this is an atypical surface to investigate, the in-depth supplemental information provides a useful guide and the authors carefully walk the readers through the concepts. They also show how to generalize the concept through a least common multiple criterion that can be applied to other material systems.

Given the novelty, clarity, and potential impact of the work, I recommend the manuscript for publication in Nature Communications. The only change that needs to be made is that they use the acronym SFAL at the bottom of page 6 before it is defined on page 7. With the small change, the manuscript should be published in its current form.

Response to Reviewer #1

Thank you very much for your highly positive assessment of our manuscript and for recommending it for publication in *Nature Communications*. We sincerely appreciate your recognition of the novelty, clarity, and potential impact of our work, as well as the comments on the strength of the combined ARPES and DFT analysis and the usefulness of the Supplemental Information.

Regarding the only issue raised, we have corrected the manuscript so that the acronym SFAL is no longer used before it is defined (the definition now appears at its first occurrence, prior to any subsequent use). This change has been implemented in the revised version.

Reviewer #2 (Remarks to the Author):

In this manuscript, the authors investigate the electronic structure of the low-symmetry (103) surface of the Weyl semimetal NdAlSi using ARPES and DFT. They report a mismatch between the conventional surface Brillouin zone (SBZ) and the projected bulk Weyl points. To resolve this, they propose a "Least Common Multiple" (LCM) framework, suggesting that the interference of multiple bulk Brillouin zones creates a "momentum-space moiré" pattern. They further claim the observation of Surface Fermi Arc Loops (SFALs).
The experimental data, particularly the preparation of the (103) surface and the high-resolution ARPES data, is of high quality, however, I have a few concerns regarding the theoretical interpretation and the conceptual framing of the results. The authors use strong terminology, e. g. "Moiré Topology", "New Paradigm", to describe what appears to be standard surface reconstruction/folding physics. Furthermore, there is a clear discrepancy between the proposed theory and the experimental data regarding the topological connectivity (loops vs. arcs) that is not adequately resolved.

1. The "Moiré" Terminology: The authors frame the observed periodicity mismatch and reconstruction as "momentum-space moiré topology". This terminology could be potentially misleading. Nowadays, "Moiré physics" (e.g., in twisted bilayers) implies a specific real-space interference leading to large-scale structural modulations and emergent flat bands due to twist or lattice mismatch. In this work, the phenomenon described seems to be a classic case of a surface reconstruction or a supercell effect required to satisfy boundary conditions on a vicinal/high-index surface. The "interference" described is simply the folding of bands due to the new surface potential periodicity.
The authors need to explicitly justify why this should be classified as "Moiré" physics rather than standard "surface reconstruction" or "Umklapp folding." Does this mechanism lead to the localized states or correlation physics typical of Moiré systems? If not, the terminology should be toned down to avoid misleading readers.

2. Major Discrepancy between Theory and Experiment (SFALs):
A central claim of the paper is the observation of "Closed Fermi-arc loops (SFALs)". However, the authors' DFT calculations based on the LCM framework fail to reproduce these loops, yielding open arcs instead. The manuscript attributes this discrepancy to "zigzag-type reconstructions" or "mixed local terminations" not captured by the slab model.
This is a significant weakness. If the "LCM framework" is the "universal" solution to the bulk-boundary correspondence paradox, why does it fail to predict the most striking topological feature (the closed loops) in this work? The authors cannot claim the framework "quantitatively reproduces" the spectra while simultaneously admitting it fails to capture the topological connectivity (loops vs. open arcs). The authors should either provide a calculation that reproduces the loops (e.g., using the mixed termination they hypothesize) or significantly retreat from the claim that their theoretical framework fully explains the data.

3. The "LCM Criterion" – Is it Trivial?
The authors present the "Least Common Multiple (LCM)" criterion as a new discovery. From

a crystallographic perspective, finding the commensurability between a bulk projection vector and a surface reciprocal lattice vector is a standard procedure to determine the surface supercell size. It is mathematically necessary that the projection closes after N unit cells if the slope is rational. I would suggest the authors to clarify: What is physically new about the LCM criterion beyond standard crystallographic projection arithmetic? The novelty seems to lie in its application to Weyl points, but the geometric principle itself appears well-established.

4. Surface vs. Bulk State Distinction:
The identification of surface states relies heavily on comparing "flat" regions (surface states) vs. "uneven/disordered" regions (bulk states). While the "disorder filter" argument is plausible, it is possible that the "new smaller SBZ" is simply a result of a physical surface reconstruction (i.e., atoms moving to new equilibrium positions) rather than just the electronic interference of bulk projections?
Also, the DFT calculations assume a specific ideal termination (Si-Al). Did the authors perform structural relaxation in their DFT? If the atoms physically reconstruct to a 3x1 supercell, then the electronic folding is a trivial consequence of the structural change, not necessarily a "hidden topology." This distinction is crucial.

5. Magnetic Order:
NdAlSi is a magnetic material (Nd moments). The authors state it is non-magnetic at the measurement temperature (paramagnetic) and use a non-magnetic Nd pseudopotential. Even in the paramagnetic phase, local moments and spin fluctuations can be significant, especially for surface states. Given that Weyl physics is sensitive to time-reversal symmetry, can the authors rule out that the "closed loops" are not a result of surface magnetism or surface-induced magnetic ordering, rather than the LCM mechanism?

In summary, this work shows very nice data on a complex system. However, the theoretical packaging ("Moiré") feels forced, and the failure of the proposed model to reproduce the key topological feature (closed loops) suggests the physical picture is incomplete. Before further consideration, I suggest that the authors provide a more rigorous reconciliation of the discrepancy between the theoretical model and the experimental results; and use a precise physical definition of the observed phenomena, avoiding overhyped terminology.

Response to Reviewer #2

In this manuscript, the authors investigate the electronic structure of the low-symmetry (103) surface of the Weyl semimetal NdAlSi using ARPES and DFT. They report a mismatch between the conventional surface Brillouin zone (SBZ) and the projected bulk Weyl points. To resolve this, they propose a "Least Common Multiple" (LCM) framework, suggesting that the interference of multiple bulk Brillouin zones creates a "momentum-space moiré" pattern. They further claim the observation of Surface Fermi Arc Loops (SFALs).
The experimental data, particularly the preparation of the (103) surface and the high-resolution ARPES data, is of high quality, however, I have a few concerns regarding the

theoretical interpretation and the conceptual framing of the results. The authors use strong terminology, e. g. "Moiré Topology", "New Paradigm", to describe what appears to be standard surface reconstruction/folding physics. Furthermore, there is a clear discrepancy between the proposed theory and the experimental data regarding the topological connectivity (loops vs. arcs) that is not adequately resolved.

We appreciate the careful reading of our manuscript and for the constructive comments. We appreciate the positive assessment of the experimental work, particularly the high-quality (103) surface preparation and the high-resolution ARPES measurements.

The concerns regarding the theoretical interpretation and conceptual framing is something we have seriously considered and we have revised the manuscript to make the central physical message more explicit. In particular, we clarify why the concept of momentum-space moiré (topological) modulation is not merely a stylistic choice, but a natural and appropriate framework required by the low-symmetry bulk–surface mismatch: a purely phenomenological "surface folding/reconstruction" viewpoint tends to treat the surface and bulk Brillouin-zone (BZ) periodicities separately, which on a low-symmetry facet of a Weyl semimetal leads to an apparent bulk–boundary correspondence paradox—surface Fermi-arc states should respect the SBZ periodicity, while the projected Weyl nodes inherit the bulk BZ periodicity, yet topology binds the two. Our LCM-based construction resolves this by showing that the superposition of phase-shifted projections from multiple bulk Brillouin zones generates an emergent new-period modulation in k-space, yielding a consistent reconstructed boundary periodicity that restores a well-defined bulk–boundary correspondence. We also expand the discussion of the experimentally observed connectivity (loops versus arcs) and clarify how it should be interpreted within this reconstructed boundary electronic structure.

Below, we provide a point-by-point response to the reviewers specific comments.

1. The "Moiré" Terminology: The authors frame the observed periodicity mismatch and reconstruction as "momentum-space moiré topology". This terminology could be potentially misleading. Nowadays, "Moiré physics" (e.g., in twisted bilayers) implies a specific real-space interference leading to large-scale structural modulations and emergent flat bands due to twist or lattice mismatch. In this work, the phenomenon described seems to be a classic case of a surface reconstruction or a supercell effect required to satisfy boundary conditions on a vicinal/high-index surface. The "interference" described is simply the folding of bands due to the new surface potential periodicity.
The authors need to explicitly justify why this should be classified as "Moiré" physics rather than standard "surface reconstruction" or "Umklapp folding." Does this mechanism lead to the localized states or correlation physics typical of Moiré systems? If not, the terminology should be toned down to avoid misleading readers.

We thank the reviewer for raising this important point. We emphasize that Moiré is fundamentally a mathematical concept introduced long before the recent surge of interest in twisted bilayers: it describes an emergent new-period modulation generated by the superposition (interference) of two or more periodic structures with a relative mismatch in lattice constant, orientation, or phase, and

it is not restricted to real space. The popularity of twisted/lattice-mismatched bilayers has made real-space Moiré superlattices and flat bands a particularly visible and influential example, and in some 2D-material contexts "Moiré physics" is used almost synonymously with that specific scenario. In the present work, however, the relevant periodic structures are defined in reciprocal space: on a low-symmetry surface the conventional SBZ periodicity and the projected bulk BZ periodicity are mismatched, and their superposition produces phase-shifted replicas and an emergent new-period modulation in momentum space. We therefore use the term "momentum-space Moiré modulation" to make clear that the interference and the resulting moiré-like modulation occur in k-space, rather than arising from a real-space Moiré superlattice.

At the same time, we agree with the reviewer's phenomenological description that, when viewed purely from the surface side, the effect can be cast as a supercell/surface-reconstruction–like band folding required to satisfy boundary conditions on a vicinal/high-index surface, i.e., an effective new surface potential periodicity leading to Umklapp replicas. Our point is that this interpretation, while correct at the level of phenomenology, implicitly treats the SBZ periodicity and the projected bulk BZ periodicity as separate ingredients—an approach that has been commonly adopted in modeling high-symmetry surfaces, where the two are naturally commensurate. For a low-symmetry surface of a Weyl semimetal, however, this separation leads to an apparent paradox: the surface Fermi arcs should be SBZ-periodic, while the projected Weyl nodes are a bulk property and follow the bulk BZ periodicity; yet bulk–boundary correspondence ties the arcs to the Weyl nodes, so a naive treatment would suggest a topological connectivity whose periodicities do not match.

It is precisely in this context that we seek a more fundamental, "bottom-up" description. By explicitly considering the superposition of multiple projected bulk Brillouin zones onto the low-symmetry surface, one naturally obtains a new emergent period modulation in momentum space (set by the closure/LCM condition), which resolves the above mismatch: the phase-shifted bulk projections generate the same reduced periodicity that one would phenomenologically attribute to a surface supercell. In other words, the "new SBZ" inferred from an effective surface supercell and the "Moire-like" period obtained from the interference of higher-order bulk projections are identical, but the latter makes transparent why this reduced periodicity is required to maintain a consistent bulk–boundary correspondence on low-symmetry facets. In this sense, introducing the concept of momentum-space Moiré (topological) modulation is not an over-claim; it is an appropriate and compact way to describe the emergent k-space new-period structure arising from bulk–surface incommensurability, and to highlight the essential physics that is obscured in a purely phenomenological folding picture.

2. Major Discrepancy between Theory and Experiment (SFALs):
A central claim of the paper is the observation of "Closed Fermi-arc loops (SFALs)". However, the authors' DFT calculations based on the LCM framework fail to reproduce these loops, yielding open arcs instead. The manuscript attributes this discrepancy to "zigzag-type reconstructions" or "mixed local terminations" not captured by the slab model.
This is a significant weakness. If the "LCM framework" is the "universal" solution to the bulk-boundary correspondence paradox, why does it fail to predict the most striking topological feature (the closed loops) in this work? The authors cannot claim the framework

"quantitatively reproduces" the spectra while simultaneously admitting it fails to capture the topological connectivity (loops vs. open arcs). The authors should either provide a calculation that reproduces the loops (e.g., using the mixed termination they hypothesize) or significantly retreat from the claim that their theoretical framework fully explains the data.

We thank the reviewer for this comment. The key point is that the reviewer's criticism mixes two logically different levels of our analysis.

Why the LCM framework does not "fail" even if the slab does not close the loops.
The LCM framework is introduced to resolve a periodicity/commensuration problem that arises on low-symmetry facets: in a purely phenomenological picture one tends to discuss the surface Brillouin-zone (SBZ) periodicity and the projected bulk Brillouin-zone periodicity separately, which on a Weyl semimetal surface can produce an apparent bulk–boundary correspondence paradox (surface states should respect SBZ periodicity, while the projected Weyl nodes inherit bulk BZ periodicity, yet topology binds them). The role of the LCM construction is to reconcile these periodicities and determine the minimal number of projected bulk BZ replicas that must be superposed to recover the reconstructed periodicity (three in the present case). This is the "universal" part: it addresses the commensuration constraint that any realistic boundary description must satisfy.

Whether the connectivity inside the reduced SBZ appears as open arcs or closed loop-like trajectories is a second-level question that depends on microscopic boundary conditions and hybridization strengths (termination mixing, reconstruction/disorder, surface potential details, slab thickness and parameter choices such as SOC), which are not uniquely fixed by commensuration alone and are not fully captured by an idealized single-termination slab model. Therefore, the absence of loop closure in the present LCM-consistent slab calculation should be understood as a limitation of the specific boundary model, not a breakdown of the LCM framework itself.

To avoid any potential misunderstanding, we have revised the manuscript to clearly distinguish the scope of the LCM framework (which resolves the bulk–surface commensuration/periodicity) from the boundary-sensitive microscopic ingredients that control the detailed arc-to-loop connectivity.

The revised text now reads: " *In ARPES, loop-like surface connectivity (SFALs) is observed within the reduced SBZ, whereas the present LCM-consistent slab calculation captures the reconstructed periodicity and the dominant replica structure but yields predominantly open-arc connectivity. This difference indicates that the LCM framework resolves the bulk - surface commensuration (periodicity) required for a consistent bulk - boundary correspondence on a low-symmetry facet, while the detailed arc-to-loop connectivity is additionally controlled by microscopic boundary conditions (e.g., termination mixing, surface reconstruction/disorder, and enhanced hybridization among laterally shifted bulk-projection replicas) that are not uniquely fixed in an idealized slab model.*" **(On page 7 marked by red words)**

3. The "LCM Criterion" – Is it Trivial?
The authors present the "Least Common Multiple (LCM)" criterion as a new discovery. From a crystallographic perspective, finding the commensurability between a bulk projection vector

and a surface reciprocal lattice vector is a standard procedure to determine the surface supercell size. It is mathematically necessary that the projection closes after N unit cells if the slope is rational. I would suggest the authors to clarify: What is physically new about the LCM criterion beyond standard crystallographic projection arithmetic? The novelty seems to lie in its application to Weyl points, but the geometric principle itself appears well-established.

We agree that, from a purely crystallographic viewpoint, determining commensurability between a projected bulk reciprocal vector and the surface reciprocal lattice—i.e., that a rational "slope" implies closure after an integer number of surface unit cells—is mathematically standard and, in hindsight, necessary.

Our intended point is that the LCM criterion here is not claimed as a new piece of crystallographic arithmetic per se, but rather as a rigorous and previously underappreciated constraint that becomes essential on low-symmetry facets and has direct physical consequences for topological bulk–boundary correspondence in Weyl semimetals. Most prior work on Weyl semimetals has focused on low-index, high-symmetry surfaces, where the projected bulk BZ is typically commensurate with the SBZ. In that situation, it is usually adequate—and common practice—to treat the surface-state dispersion and the projected bulk features largely independently, without running into periodicity-related inconsistencies. By contrast, on a low-symmetry surface the projected bulk BZ can be intrinsically shifted relative to the SBZ, and treating "surface periodicity" and "bulk periodicity" as separate ingredients—precisely the phenomenological viewpoint noted in the reviewer's Comment 1 (supercell/folding due to a new surface periodicity)—naturally leads to an apparent bulk–boundary correspondence paradox: surface Fermi-arc states should respect SBZ periodicity, whereas Weyl nodes are bulk objects and inherit bulk-BZ periodicity, yet topology binds the arcs to the nodes.

In this setting, the LCM criterion provides a strict, quantitative, and general resolution: it specifies the minimal number of projected bulk BZ replicas that must be superposed to obtain the reconstructed reduced SBZ periodicity in momentum space, thereby restoring a consistent framework in which topological connectivity can be formulated on low-symmetry facets. In other words, what is physically new is not the existence of commensuration mathematics, but (i) recognizing that this commensuration is the missing ingredient behind the bulk–surface mismatch problem in Weyl semimetals, (ii) formulating it as an operational criterion that directly dictates the emergent momentum-space periodicity, and (iii) demonstrating experimentally (via ARPES) and theoretically (via DFT) that this criterion controls the boundary reconstruction relevant to topological bulk–boundary correspondence.

To avoid over-claiming, we will revise the manuscript to explicitly acknowledge the crystallographic origin of the construction and to emphasize that the novelty lies in its application and physical consequences for topological boundary states on low-symmetry surfaces, where this rigorous constraint has not been widely appreciated or exploited in the Weyl-semimetal context.

The revised text now reads: "*More broadly, the LCM framework provides a strict and operational way to restore a consistent BBC on low-symmetry facets: it determines the minimal number of projected bulk BZs that must be superposed for the projection to close, thereby fixing the emergent reduced boundary periodicity in momentum space and the associated reconstructed surface spectrum.***" (On page 9 marked by red words)**

4. Surface vs. Bulk State Distinction:
The identification of surface states relies heavily on comparing "flat" regions (surface states) vs. "uneven/disordered" regions (bulk states). While the "disorder filter" argument is plausible, it is possible that the "new smaller SBZ" is simply a result of a physical surface reconstruction (i.e., atoms moving to new equilibrium positions) rather than just the electronic interference of bulk projections?
Also, the DFT calculations assume a specific ideal termination (Si-Al). Did the authors perform structural relaxation in their DFT? If the atoms physically reconstruct to a 3x1 supercell, then the electronic folding is a trivial consequence of the structural change, not necessarily a "hidden topology." This distinction is crucial.

We thank the reviewer for raising this important point and we agree that distinguishing a real-space structural reconstruction from a k-space commensuration/interference effect is crucial.

(1) Is the "new smaller SBZ" simply due to a physical surface reconstruction?
In principle, any real-space surface supercell (e.g., a 3×1 reconstruction) would indeed produce band folding. However, the central result of our work is that, for the (103) facet, the emergence of a reduced periodicity is already dictated by a strict commensuration requirement between the projected bulk reciprocal lattice and the surface reciprocal lattice, quantified by the LCM criterion. This derivation does not assume any atomic reconstruction: it is a geometric/kinematic constraint of the low-symmetry projection that determines the minimal closure (here N=3) required to reconcile bulk and surface periodicities.

The "disorder filter" analysis is therefore not used to define the reduced SBZ; rather, it is used to separate surface-localized spectral weight from bulk-like contributions in ARPES. The reduced periodicity itself is independently fixed by the LCM commensuration and is then observed experimentally through the appearance of phase-shifted replicas consistent with that commensurate closure.

Moreover, if one insists on explaining the reduced SBZ purely by atoms moving to new equilibrium positions, the required reconstruction would have to simultaneously satisfy the full 2D commensuration imposed by the low-symmetry (103) lattice. In this case, the physically allowed surface supercell that restores commensuration would generically be larger than a simple 3×1 construction (i.e., not merely a minimal textbook folding), because the mismatch originates from the bulk–surface projection geometry rather than from a single real-space modulation wavevector.

(2) **Did we structurally relax the DFT slabs, and do they reconstruct?**
Yes. We performed structural relaxation for the slab calculations (including the Si–Al termination

used in the manuscript). Upon relaxation, the surface primarily exhibits local atomic relaxations (e.g., interlayer spacing adjustments), but we do not find a spontaneous reconstruction into a 3×1 (or other lateral) supercell. In other words, within DFT the ideal 1×1 lateral periodicity remains stable, and the calculations do not indicate that a 3×1 atomic reconstruction is the driving origin of the reduced periodicity.

5. Magnetic Order:
NdAlSi is a magnetic material (Nd moments). The authors state it is non-magnetic at the measurement temperature (paramagnetic) and use a non-magnetic Nd pseudopotential. Even in the paramagnetic phase, local moments and spin fluctuations can be significant, especially for surface states. Given that Weyl physics is sensitive to time-reversal symmetry, can the authors rule out that the "closed loops" are not a result of surface magnetism or surface-induced magnetic ordering, rather than the LCM mechanze1ism?

We thank the reviewer for this insightful suggestion and for offering an alternative perspective on the possible origin of the closed loops. While we cannot strictly rule out subtle surface-enhanced magnetic correlations, we consider it unlikely that surface magnetism is the primary cause of the observed loop-like connectivity. The magnetism in NdAlSi arises from localized Nd 4f moments, which are strongly shielded and couple to the near-Fermi level itinerant states mainly through relatively weak indirect (RKKY-type) exchange [Nature Communications **14**, 7185 (2023)]; in the paramagnetic regime such effects are expected to be very weak and largely incoherent for the low-energy dispersions relevant to our ARPES observations.

In summary, this work shows very nice data on a complex system. However, the theoretical packaging ("Moiré") feels forced, and the failure of the proposed model to reproduce the key topological feature (closed loops) suggests the physical picture is incomplete. Before further consideration, I suggest that the authors provide a more rigorous reconciliation of the discrepancy between the theoretical model and the experimental results; and use a precise physical definition of the observed phenomena, avoiding overhyped terminology.

We emphasize that the “momentum-space Moiré” description is not a forced packaging but follows naturally from the central low-symmetry issue addressed here: the intrinsic mismatch between the SBZ periodicity and the projected bulk-BZ periodicity leads, under a purely phenomenological separation of “surface folding” and “bulk projections,” to an apparent bulk–boundary correspondence paradox in a Weyl semimetal. The LCM framework resolves this at a fundamental level by establishing the minimal commensurate closure (three-zone superposition) and the emergent reduced periodicity/replica structure in k-space in precisely the sense in which the modulation is Moiré-like (interference between mismatched periodicities in momentum space, not a real-space Moiré lattice). We also agree that the current idealized slab does not reproduce the loop closure, and we have revised the manuscript to reconcile this more rigorously by clearly distinguishing what the LCM framework fixes robustly (periodicity/replicas and restored commensuration) from the boundary-sensitive arc-to-loop connectivity, which additionally depends on microscopic surface conditions (termination mixing, reconstruction/disorder, hybridization) not uniquely captured in a minimal slab model, while keeping the terminology and claims strictly tied to these physical definitions.

Reviewer #3 (Remarks to the Author):

In this study, C. Li et al. have used synchrotron-based ARPES and DFT-based surface Green's function methods to study the topological surface states and Fermi arcs on the low-symmetry/high-Miller-index (103) surface of NdAlSi, an inversion-breaking Weyl semimetal, above its magnetic phase transition. By using the monoclinic supercell in Fig.S9(b) with the whole (103) surface cell as the side surface, the calculated spectral functions on the semi-infinite (103) surface explain well the observed ARPES data for both flat region with Si termination in Fig.1(f) and disordered region with multiple terminations in Fig.1(e). The authors did a detailed analysis of bulk-to-surface Brillouin zone (BZ) projection and a systematic comparison between ARPES and topological surface state calculations in Fig.2, 3 and 4.

However, the manuscript has many overclaims and even misconception, which make it unacceptable for the high standard of Nat. Comm. Here are the main comments.

The abstract claims that "bulk and surface periodicities are inherently mismatched" by showing the partial (103) surface region in Fig.1(a) for a zigzag pattern with a single bulk unit cell. But it is well known that the high Miller index surfaces often span multiple bulk unit cells. For example, for groups of FCC high Miller index surfaces, please see Fig.15 Pt(310) in Santos et al. Results in Surfaces and Interfaces 3, 10006 (2021). The proper choice of the NdAlSi (103) surface unit cell is not Fig1.(a) but actually Fig.S9(b). Such a surface supercell can be obtained more intuitively by the real space axis intercept method, which is equivalent to the least-common-multiple (LCM) method in reciprocal space as the authors detailed in Supplementary Materials. The (103) surface periodicity still has the same underlying bulk periodicity as any other surface orientations. The "new smaller SBZ" (green in Fig.1(b-c)) comes naturally when the proper (103) surface unit cell is chosen in Fig.S9(b), which is more intuitive from the real space intercept method. Thus, there is no inherent mismatch between the bulk and surface periodicities. The proper (103) surface unit cell in Fig.S9(b) should be included in the main text Fig.1.

The manuscript also claims in the title, abstract and other places of the so-called "hidden moire in reciprocal space", "translational commensurability is lost" and "bulk-surface incommensurability". But there are no extra competing interactions beside the bulk projection to impose any additional long periodicity on the surface. There is no moire-like flat-band in the NdAlSi (103) surface spectral functions as shown in data and figures. The SBZ is perfectly commensurate with the bulk BZ when not a single but the whole 3D reciprocal space (including the Weyl points) is properly projected into the 2D sheet for SBZ, which is equivalent to the semi-infinite surface Green's function. As the authors also noted on p.3 that "the mismatch is not a breakdown of BBC but a natural outcome of incomplete bulk projection". Thus, there is no lost translational commensurability between bulk and surface, but the need of proper and complete bulk-to-surface projection.

The need of multiple bulk BZ projection to SBZ to fully account for the topological surface

states is not uncommon. Even for the high-symmetry rock-salt (001) surface (please see Fig.4 in Kuroda et al, PRL 120, 086402 (2018)), where the projection of two bulk BZ is needed for the band inversion at the X points to fully explain the gapped surface Dirac cones at the M point of the (001) SBZ.

Thus, given that these key claims in the manuscript are exaggerated, the low-symmetry/high-index surface of inversion-breaking Weyl semimetal such as TaAs have already been studied by Zheng et al in Ref.19, the current manuscript does not meet the high standard of Nat. Comm.

Response to Reviewer #3

In this study, C. Li et al. have used synchrotron-based ARPES and DFT-based surface Green's function methods to study the topological surface states and Fermi arcs on the low-symmetry/high-Miller-index (103) surface of NdAlSi, an inversion-breaking Weyl semimetal, above its magnetic phase transition. By using the monoclinic supercell in Fig.S9(b) with the whole (103) surface cell as the side surface, the calculated spectral functions on the semi-infinite (103) surface explain well the observed ARPES data for both flat region with Si termination in Fig.1(f) and disordered region with multiple terminations in Fig.1(e). The authors did a detailed analysis of bulk-to-surface Brillouin zone (BZ) projection and a systematic comparison between ARPES and topological surface state calculations in Fig.2, 3 and 4.

However, the manuscript has many overclaims and even misconception, which make it unacceptable for the high standard of Nat. Comm. Here are the main comments.

The reviewers careful reading of our manuscript and is gratefully acknowledged as are the detailed comments. We appreciate the recognition of the quality of the synchrotron ARPES data, the surface Green's function calculations based on the monoclinic supercell, and the systematic bulk–surface BZ projection analysis and ARPES–DFT comparisons in Figs. 2–4.

We also take the concerns regarding potential overclaims or misconceptions very seriously. In the revised manuscript, we have carefully re-examined the presentation and strengthened the wording and explanations to ensure that all statements are precise, well-supported, and aligned with the evidence. Below, we respond point-by-point to your main comments.

The abstract claims that "bulk and surface periodicities are inherently mismatched" by showing the partial (103) surface region in Fig.1(a) for a zigzag pattern with a single bulk unit cell. But it is well known that the high Miller index surfaces often span multiple bulk unit cells. For example, for groups of FCC high Miller index surfaces, please see Fig.15 Pt(310) in Santos et al. Results in Surfaces and Interfaces 3, 10006 (2021). The proper choice of the NdAlSi (103) surface unit cell is not Fig1.(a) but actually Fig.S9(b). Such a surface supercell can be obtained more intuitively by the real space axis intercept method, which is equivalent to the least-common-multiple (LCM) method in reciprocal space as the authors detailed in

Supplementary Materials. The (103) surface periodicity still has the same underlying bulk periodicity as any other surface orientations. The "new smaller SBZ" (green in Fig.1(b-c)) comes naturally when the proper (103) surface unit cell is chosen in Fig.S9(b), which is more intuitive from the real space intercept method. Thus, there is no inherent mismatch between the bulk and surface periodicities. The proper (103) surface unit cell in Fig.S9(b) should be included in the main text Fig.1.

We thank the reviewer for this comment. We agree that, from a crystallographic and phenomenological viewpoint, one can choose a larger commensurate surface supercell for a high-Miller-index facet—such as the construction shown in Fig. S9(b), which is naturally obtained via the real-space intercept method (and is equivalent to our reciprocal-space LCM closure). We stress, however, that this intercept-based truncation is only one valid way to represent/cut the same (103) plane and does not exclude other symmetry-equivalent cleavage registries. In fact, consistent with this, we experimentally observe multiple distinct surface electronic structures on different spatial regions of the same cleave (three representative cases are shown in Fig. S10, and additional terminations are likely), and two of these cannot be captured by the specific slab termination implied by Fig. S9(b) (see Fig. S11). This is precisely why we start from the primitive-cell/Bloch perspective (Fig. 1(a)), where the zigzag pattern reflects the equivalence of neighboring primitive cells under cleavage along (103) and provides a mathematically well-defined route to understand how the LCM-based commensuration emerges and why multiple local boundary realizations can occur on the same macroscopic surface.

Our use of the phrase "bulk and surface periodicities are inherently mismatched" was intended in a specific and restricted sense, and we recognize it may have been misunderstood. By "mismatched" we mean that, on a low-symmetry surface, the periodicity of the conventional first SBZ (the natural framework used to discuss surface electronic states) is generally inconsistent with the periodicity implied if one considers only the first bulk BZ (where Weyl nodes are typically identified and discussed). Thus, the mismatch we refer to is between the first-SBZ and first-bulk-BZ descriptions, rather than any claim that the underlying crystal cannot be made commensurate. Importantly, this issue is not removed simply by changing the surface real-space representation—for example, replacing the primitive-cell zigzag picture in Fig. 1(a) by an intercept-constructed surface supercell such as Fig. S9(b). Even with such a surface choice, if the bulk is still treated only within the first bulk BZ, the projected Weyl nodes do not, in general, map one-to-one into the conventional first SBZ on a low-symmetry facet. This "surface SBZ vs. bulk BZ treated independently" viewpoint is exactly what generates the apparent bulk–boundary correspondence paradox in this geometry.

The purpose of the LCM criterion (and the associated momentum-space moiré modulation picture) is to show that a consistent resolution requires superposing multiple-order projected bulk BZs: the resulting phase-shifted replicas generate an emergent new-period modulation and a reduced periodicity in momentum space, which coincides with the reconstructed/reduced SBZ one may also infer from a commensurate supercell construction. In this sense, the "new smaller SBZ" is not introduced to claim any intrinsic breakdown of crystalline periodicity, but to make explicit—step by step—how the effective boundary periodicity required by bulk–boundary correspondence emerges once one properly accounts for the superposition of multiple projected bulk Brillouin

zones at the boundary, i.e., beyond the restriction to only the first bulk BZ in the bulk-to-surface projection.

Regarding Fig. 1(a), the zigzag depiction is motivated by the primitive-cell (Bloch-theorem) viewpoint: from the underlying primitive lattice, adjacent primitive cells are equivalent, so the cleavage of a low-symmetry plane naturally samples equivalent breaking positions in a way that appears as a zigzag motif when represented using a single bulk unit. Starting directly from the larger supercell in Fig. S9(b) is of course possible, but it implicitly bundles three equivalent primitive cells into one object and can obscure the conceptual route by which the momentum-space interference/LCM closure resolves the low-symmetry bulk–surface correspondence issue.

The manuscript also claims in the title, abstract and other places of the so-called "hidden moire in reciprocal space", "translational commensurability is lost" and "bulk-surface incommensurability". But there are no extra competing interactions beside the bulk projection to impose any additional long periodicity on the surface. There is no moire-like flat-band in the NdAlSi (103) surface spectral functions as shown in data and figures. The SBZ is perfectly commensurate with the bulk BZ when not a single but the whole 3D reciprocal space (including the Weyl points) is properly projected into the 2D sheet for SBZ, which is equivalent to the semi-infinite surface Green's function. As the authors also noted on p.3 that "the mismatch is not a breakdown of BBC but a natural outcome of incomplete bulk projection". Thus, there is no lost translational commensurability between bulk and surface, but the need of proper and complete bulk-to-surface projection.

The need of multiple bulk BZ projection to SBZ to fully account for the topological surface states is not uncommon. Even for the high-symmetry rock-salt (001) surface (please see Fig.4 in Kuroda et al, PRL 120, 086402 (2018)), where the projection of two bulk BZ is needed for the band inversion at the X points to fully explain the gapped surface Dirac cones at the M point of the (001) SBZ.

We agree the reviewer for this comment. We believe there is some misunderstanding of what we mean by "hidden Moiré in reciprocal space," "lost translational commensurability," and "bulk–surface incommensurability," and how these relate to the LCM criterion and the resolution of the low-symmetry bulk–boundary correspondence issue.

First, we do not invoke any additional competing interaction beyond crystalline translation symmetry, nor do we claim that NdAlSi(103) already exhibits a pronounced moiré flat band in the present data. Our use of "moiré" follows its general mathematical meaning—an emergent new-period modulation from the superposition of two phase mismatched periodic structures—and in our case the superposition occurs in k-space through the boundary's reception of multiple laterally shifted projections of bulk BZs.

From a strict mathematical standpoint, both bulk and surface translational periodicities are defined by the primitive cell implied by Bloch's theorem. The LCM criterion is a universal consequence of this definition on low-symmetry/high-index facets: the projected bulk reciprocal lattice generally

appears as phase-shifted replicas relative to the conventional SBZ, and one must include a finite number of projected bulk BZ copies before the projection "closes" and a consistent reduced periodicity emerges. This statement is independent of whether one adopts the phenomenological truncation shown in Fig. S9(b) or the primitive-cell (zigzag) representation in Fig. 1(a): the LCM closure is a property of the projection geometry itself and applies to both constructions.

Second, when we refer to "incommensurability" or "lost commensurability," we are not claiming that the crystal truly lacks translational order. Rather, we refer to the apparent mismatch that arises in the conventional, phenomenological first-zone description, where surface states are discussed in the conventional first SBZ while bulk Weyl nodes are identified within the first bulk BZ. On a low-symmetry facet these two "first-zone" descriptions are generally not in one-to-one registry, which produces the apparent bulk–boundary correspondence paradox. This is exactly consistent with our statement on p.3 that the mismatch is a natural outcome of incomplete bulk projection.

In this sense, our wording "hidden moiré in reciprocal space / bulk–surface incommensurability / lost commensurability" is not meant to imply any additional competing interaction or real-space structural Moiré superlattice. Rather, it refers to the emergence of a new-period modulation in k-space that results when multiple, laterally shifted bulk-zone projections are superposed at the boundary—i.e., the same "complete projection" viewpoint that the reviewer advocates. The "Moiré" terminology is used in its general definition (an emergent new-period modulation from superposing mismatched periodicities), and here the modulation occurs in momentum space through the interference/superposition of higher-order bulk projections; its period is exactly the LCM-determined reduced periodicity, which coincides with the reduced SBZ one would infer phenomenologically from an enlarged surface supercell.

Finally, we emphasize that the absence of an obvious moiré-like flat band in the present NdAlSi(103) data most likely reflects that, for this facet, the momentum-space Moiré modulation set by the LCM closure (here N=3) is relatively modest. In more extreme high-index cases the effect can be parametrically stronger: for a surface such as (10N) with N=100, the phenomenologically defined surface repeat (constructed analogously to Fig. S9(b)) corresponds to a reduced SBZ that is 1/N of the primitive-cell/Bloch-defined one, and the experimentally observed surface/topological features would become correspondingly more finely replicated and discretized in momentum space. In such a regime, the superposition (interference) of many higher-order bulk-BZ projections at the boundary—i.e., a more pronounced momentum-space moiré modulation—would be expected to enhance band folding/hybridization and could make flatter surface dispersions more likely.

In summary, we discuss a k-space Moiré modulation emerging from the superposition of multiple projected bulk Brillouin zones required by complete bulk-to-surface projection, rather than an interaction-driven real-space moiré superlattice, and "incommensurability" refers specifically to the apparent mismatch between the conventional first SBZ and a description restricted to the first bulk BZ, which is resolved once multiple higher-order bulk BZ projections are superposed (summed) on the surface.

Thus, given that these key claims in the manuscript are exaggerated, the low-symmetry/high-index surface of inversion-breaking Weyl semimetal such as TaAs have already been studied by Zheng et al in Ref.19, the current manuscript does not meet the high standard of Nat. Comm.

Based on the clarifications above, we respectfully disagree that our key claims are exaggerated. The LCM criterion and the resulting momentum-space Moiré modulation are introduced as the natural and essential framework to resolve the apparent bulk–boundary correspondence paradox that arises specifically on low-symmetry/high-index Weyl surfaces when one treats the conventional first SBZ and the first bulk BZ independently; this is a foundational issue that is not addressed by most of standard high-symmetry-surface treatments. While Ref. 19 (Zheng et al. on TaAs) indeed represents important prior progress on low-symmetry surfaces, our work starts from an experimentally observed anomaly on the (103) facet and distills from it a general, mathematically grounded principle: multiple laterally shifted projections of bulk Brillouin zones superpose and interfere at the boundary, producing a momentum-space moiré modulation, with the LCM criterion uniquely determining the minimal number of bulk-BZ projections required for closure and the emergent reduced boundary periodicity. This framework resolves the apparent bulk–boundary correspondence paradox on low-symmetry Weyl surfaces and, guided by the LCM prescription, our DFT/surface Green's-function calculations yield surface and bulk-projection spectral features that can be directly and systematically matched to the ARPES measurements, providing a transferable criterion for other materials and surface orientations. As Reviewer 1 commented "*The manuscript is well written and the arguments are clearly presented. The conclusions are straight forward and supported by the combination of theory and experiment. Given this is an atypical surface to investigate, the in-depth supplemental information provides a useful guide and the authors carefully walk the readers through the concepts. They also show how to generalize the concept through a least common multiple criterion that can be applied to other material systems.*"

Summary of changes:

1. On Page 7 in the manuscript, we revised the descriptions as "*In ARPES, loop-like surface connectivity SFALs is observed within the reduced SBZ, whereas the present LCM-consistent slab calculation captures the reconstructed periodicity and the dominant replica structure but yields predominantly open-arc connectivity. This difference indicates that the LCM framework resolves the bulk-surface commensuration (periodicity) required for a consistent bulk-boundary correspondence on a low-symmetry facet, while the detailed arc-to-loop connectivity is additionally controlled by microscopic boundary conditions (e.g., termination mixing, surface reconstruction/disorder, and enhanced hybridization among laterally shifted bulk-projection replicas) that are not uniquely fixed in an idealized slab model.*"
2. On Page 9 in the manuscript, we revised the description as "*the LCM framework provides a strict and operational way to restore a consistent BBC on low-symmetry facets: it determines the minimal number of projected bulk BZs that must be superposed for the projection to close, thereby fixing the emergent reduced boundary periodicity in momentum space and the associated reconstructed surface spectrum.*"
3. All changes are marked in red in the revised version.

Response to Reviewer's Comments

Reviewer #1 (Remarks to the Author):

After reviewing the full set of reviewer comments, the authors' rebuttal, and the revised manuscript, my overall assessment is that the revision improves the paper in meaningful ways but does not fully resolve the concerns raised during peer review. The manuscript remains interesting and potentially impactful, especially because it combines technically strong ARPES measurements with theory to study an unusual low-symmetry surface of a Weyl semimetal. The revised version is stronger than the original in that it more clearly distinguishes between what is firmly supported by the data and what remains more interpretive. At the same time, several of the most important criticisms from Reviewers 2 and 3 were only partially addressed, and in some cases the rebuttal is more careful and persuasive than the revised manuscript itself.

To the authors' credit, they responded constructively to the criticism about the discrepancy between theory and experiment regarding the claimed closed Fermi-arc loops. The revised manuscript now makes a clearer distinction between the LCM framework as a way to resolve the commensuration and periodicity problem on a low-symmetry surface, and the separate question of whether the detailed connectivity takes the form of open arcs or closed loops. The paper now acknowledges that the LCM-consistent slab calculation reproduces the reconstructed periodicity and dominant replica structure, while the loop-like connectivity likely depends on additional microscopic surface conditions such as termination mixing, reconstruction, disorder, or enhanced hybridization. That clarification strengthens the paper and directly addresses one of the most serious concerns of the Reviewers.

However, the revisions create a problem with internal consistency. The manuscript still contains language, particularly in the title and abstract, that overstates the level of agreement between theory and experiment. The abstract continues to suggest that the calculations quantitatively reproduce the observed spectra, even though the body of the paper now admits that the most visually striking feature, namely the loop-like connectivity, is not reproduced by the idealized slab model. That tension is insufficiently resolved. More broadly, both Reviewers 2 and 3 objected to the strongest conceptual language. The rebuttal offers a thoughtful defense of "momentum-space moiré" as a broader mathematical description of emergent modulation in reciprocal space, but the revised manuscript still retains much of the same expansive framing, including the title "Hidden Moiré Topology," references to a "new paradigm," and suggestions of flat-band engineering.

Reviewer 2's concerns were therefore mostly fair, especially regarding the mismatch between the scope of the theory and the way the results are advertised. Reviewer 3 also raised some legitimate points, particularly about the risk of overstating the novelty of the LCM construction itself, which the authors appropriately softened by clarifying that the novelty lies in its topological application rather than in new crystallographic mathematics. However, Reviewer 3's implication that the main phenomenon is merely a routine supercell issue or that the work lacks sufficient novelty altogether

seems overly critical. Even if one prefers a more conventional crystallographic description, the data do support a real low-symmetry surface projection problem and a meaningful LCM-guided resolution of the periodicity mismatch.

The manuscript revision is improved, scientifically stronger, and is still suitable for Nature Communications but the revisions create internal inconsistencies and do not fully resolve the criticism of Reviews 2 and 3. It is recommended that the manuscript be revised to address these issues prior to publication.

Response to Reviewer #1

After reviewing the full set of reviewer comments, the authors' rebuttal, and the revised manuscript, my overall assessment is that the revision improves the paper in meaningful ways but does not fully resolve the concerns raised during peer review. The manuscript remains interesting and potentially impactful, especially because it combines technically strong ARPES measurements with theory to study an unusual low-symmetry surface of a Weyl semimetal. The revised version is stronger than the original in that it more clearly distinguishes between what is firmly supported by the data and what remains more interpretive. At the same time, several of the most important criticisms from Reviewers 2 and 3 were only partially addressed, and in some cases the rebuttal is more careful and persuasive than the revised manuscript itself.

We thank the reviewer for the thorough evaluation of the revised manuscript and rebuttal, and for recognizing the significance of combining technically strong ARPES measurements with theory to study the low-symmetry (103) surface of a Weyl semimetal. We also appreciate the reviewer's key point that, while the revision improved the manuscript, it did not fully resolve the most important concerns raised by Reviewers 2 and 3, and that in some places our rebuttal articulated the scope and conclusions more clearly than the manuscript itself. In this new revision, we have therefore carried out targeted edits to ensure that the tone and claims in the title, abstract, and main text consistently match the careful and defensible framing used in our rebuttal. In particular, we have further reduced language that could be read as over-claiming conceptual novelty, and we have sharpened the statements on what is quantitatively captured by theory versus what remains sensitive to microscopic surface conditions.

In addition, we clarify that the advance of this work is not limited to reproducing the NdAlSi(103) spectra by DFT. Using the (103) surface as an experimentally grounded case study, we elevate the analysis to an executable and reproducible mathematical framework for bulk-to-surface mapping (complete 3D-to-2D projection, fractional projection shifts, and LCM commensuration closure), and we show, mathematically and through direct experimental consistency, that it is fully equivalent to the standard intercept-based surface-cell construction and conventional surface-state calculations. This general framework both provides a transparent, broadly applicable methodology for complex surfaces and enables a distinct low-symmetry Weyl-surface consequence: phase-shifted multi-zone projections can displace and hybridize Fermi-arc manifolds, reconstructing the boundary connectivity beyond the high-symmetry picture.

To the authors' credit, they responded constructively to the criticism about the discrepancy between theory and experiment regarding the claimed closed Fermi-arc loops. The revised manuscript now makes a clearer distinction between the LCM framework as a way to resolve the commensuration and periodicity problem on a low-symmetry surface, and the separate question of whether the detailed connectivity takes the form of open arcs or closed loops. The paper now acknowledges that the LCM-consistent slab calculation reproduces the reconstructed periodicity and dominant replica structure, while the loop-like connectivity likely depends on additional microscopic surface conditions such as termination mixing, reconstruction, disorder, or enhanced hybridization. That clarification strengthens the paper and directly addresses one of the most serious concerns of the Reviewers.

We are grateful that the reviewer finds our response to the theory experiment discrepancy regarding the loop-like Fermi-arc connectivity constructive, and agrees that the revised manuscript more clearly separates two distinct issues: (i) the commensuration/periodicity problem on a low-symmetry surface, which is resolved within our LCM-based multi-BZ projection framework, and (ii) the detailed connectivity of reconstructed surface states (open arcs versus loop-like features), which is not uniquely fixed by an idealized termination-only slab model. Following the reviewer's guidance, we have strengthened this separation further and enforced it consistently throughout the manuscript. This clarification is now aligned across the abstract, results, and conclusion, and it directly addresses one of the most central concerns raised in the peer review.

However, the revisions create a problem with internal consistency. The manuscript still contains language, particularly in the title and abstract, that overstates the level of agreement between theory and experiment. The abstract continues to suggest that the calculations quantitatively reproduce the observed spectra, even though the body of the paper now admits that the most visually striking feature, namely the loop-like connectivity, is not reproduced by the idealized slab model. That tension is insufficiently resolved. More broadly, both Reviewers 2 and 3 objected to the strongest conceptual language. The rebuttal offers a thoughtful defense of "momentum-space moiré" as a broader mathematical description of emergent modulation in reciprocal space, but the revised manuscript still retains much of the same expansive framing, including the title "Hidden Moiré Topology," references to a "new paradigm," and suggestions of flat-band engineering.

We agree and have revised the manuscript to match the careful scope and framing already articulated in the rebuttal. Concretely, we implemented the following global changes to remove internal tension and avoid overclaiming:

1) We have changed the title to "**Topological Fermi arc interference on Low-Symmetry Weyl Surfaces**";

2) We have rewrite the abstract, discussion and conclusion sections. In the revised version, conceptual language was toned down to avoid framing that could be read as advertising a broader "new paradigm" beyond what is established in the present work;

3) We clarified that the LCM criterion is mathematically equivalent to the standard intercept-based surface-cell construction used in surface crystallography.

The detailed changes are marked by red words in the manuscript.

Reviewer 2's concerns were therefore mostly fair, especially regarding the mismatch between the scope of the theory and the way the results are advertised. Reviewer 3 also raised some legitimate points, particularly about the risk of overstating the novelty of the LCM construction itself, which the authors appropriately softened by clarifying that the novelty lies in its topological application rather than in new crystallographic mathematics. However, Reviewer 3's implication that the main phenomenon is merely a routine supercell issue or that the work lacks sufficient novelty altogether seems overly critical. Even if one prefers a more conventional crystallographic description, the data do support a real low-symmetry surface projection problem and a meaningful LCM-guided resolution of the periodicity mismatch.

We appreciate the reviewer's fair assessment. We agree that the concerns from Reviewers 2 and 3 regarding how the results were advertised relative to the scope of the theory were largely fair, and we have revised the manuscript accordingly. In particular, we further toned down language that could be read as overstating conceptual novelty, and we now state consistently that LCM-consistent calculations reproduce the reconstructed periodicity and dominant replica structure, while finer connectivity details can depend on microscopic boundary conditions beyond an idealized slab model.

We also appreciate the reviewer's point that the novelty of our work should not be interpreted as introducing new crystallographic mathematics. To avoid any ambiguity, we explicitly acknowledge in the main text that the LCM commensuration condition is mathematically equivalent to standard surface-cell constructions used in surface science/crystallography, and we emphasize that our contribution lies in its topological and spectroscopic application: (i) demonstrating experimentally, via ARPES, a directly observable low-symmetry surface projection/reconstruction problem with characteristic replica modulations, and (ii) providing an operational, LCM-guided route that enables consistent modeling and interpretation of the period reconstructed boundary spectrum on a Weyl surface. In this way, even when described in conventional crystallographic terms, the data still establish a nontrivial low-symmetry projection phenomenon and a practically useful commensuration guided resolution that goes beyond a routine "supercell book keeping", especially for complex low-symmetry surfaces where realistic boundaries may involve zigzag-type morphologies, mixed terminations, and enhanced reconstruction/hybridization.

The manuscript revision is improved, scientifically stronger, and is still suitable for Nature Communications but the revisions create internal inconsistencies and do not fully resolve the criticism of Reviews 2 and 3. It is recommended that the manuscript be revised to address these issues prior to publication.

We thank the reviewer for the fair and constructive recommendation. We revised manuscript and addressed the key criticisms raised by Reviewers 2 and 3 at the manuscript level, not only in the rebuttal. In response, we have implemented targeted revisions throughout the title, abstract, results, and conclusion to remove any remaining overstatements and to ensure a single, consistent

message: LCM-consistent calculations capture the reconstructed periodicity and dominant replica structure, whereas the detailed arc connectivity (including loop-like features) can depend on additional microscopic boundary conditions beyond an idealized slab model. We believe these changes resolve the internal tensions highlighted by the reviewer and bring the manuscript into closer alignment with the scope of our data and theory, thereby addressing the outstanding concerns prior to publication.

Reviewer #3 (Remarks to the Author):

In the rebuttal letter, the authors have tried mostly to argue against the concerns of Reviewer 2 and 3 on packaging overclaims and exaggerated statements with the usage of "hidden moire", "bulk and surface periodicities are inherently mismatched", "translational commensurability is lost" and "bulk-surface incommensurability", without modifying these problematic statements in the manuscript. After carefully reading the lengthy arguments that the authors have presented, this reviewer find these arguments are not convincing and the revised manuscript is not suitable for publication.

The authors have acknowledged the equivalence of their LCM and the straightforward intercept-based method that is widely accepted in surface science and crystallography communities to generate the proper surface unit cell for low-symmetry or high Miller index surfaces. It is also well-known that the surface unit cells for high Miller index surfaces, like the NdAlSi(103) here, usually span serval primitive bulk unit cells in real space, thus, in reciprocal space, the surface Brillouin zones (SBZ) need a complete projection from 3D to 2D, not just a section of the first bulk BZ. The authors have also agreed that the key finding of the good agreement between the ARPES and surface Green's function calculation relies on the proper (103) surface unit cell and the inherent complete bulk-to-surface projection from 3D to 2D in the surface Green's function calculation.

Both Reviewer 2 and 3 pointed out that such proper surface unit cell and complete bulk-to-surface projection should be the default solution to consider for high Miller index surface. The reduced SBZ compared to bulk BZ is not from the so-called "hidden moire" but the results of normal practice in surface science and crystallography. The need of complete bulk-to-surface projection is also common in surface band structure calculations using surface Green's function. Again one such example is the CeX (X=P, As, Sb and Bi) surface states (please see Fig.4 in Kuroda et al, PRL 120, 086402 (2018)). Even for the high-symmetry rock-salt (001) surface, where the projection of two bulk BZ is needed for the band inversion at the X points to fully explain the gapped surface Dirac cones at the M point of the (001) SBZ. However, the authors have ignored such example in the rebuttal letter.

If such "hidden moire" and similar overclaims are accepted for publication with just using the default practice to generate proper surface unit cell and complete bulk-to-surface projection, then should all high Miller surfaces of many other materials be called "hidden moire" from now on? Despite there is no competing interaction being introduced in the single phase of crystal structure for moire physics, but just BZ projection and folding.

Response to Reviewer #3

In the rebuttal letter, the authors have tried mostly to argue against the concerns of Reviewer 2 and 3 on packaging overclaims and exaggerated statements with the usage of "hidden moire", "bulk and surface periodicities are inherently mismatched", "translational commensurability is lost" and "bulk-surface incommensurability", without modifying these problematic statements in

the manuscript. After carefully reading the lengthy arguments that the authors have presented, this reviewer find these arguments are not convincing and the revised manuscript is not suitable for publication.

Before responding point by point, we would like to clarify what is new in this work. The manuscript is not limited to reproducing the NdAlSi(103) spectra by DFT. Rather, using the (103) surface as an experimentally grounded case study, we formulate an executable and reproducible mathematical framework that makes bulk-to-surface mapping explicit and operational: a complete 3D-to-2D projection generates fractional lateral phase shifts between neighboring bulk-zone projections, and an LCM commensuration condition determines their minimal closure and the resulting reconstructed surface periodicity. Within this framework we predict—and experimentally demonstrate—a topological boundary reconstruction specific to low-symmetry Weyl surfaces: phase-shifted multi-zone projections displace the surface-Fermi-arc manifolds so that they overlap and hybridize, producing replica-modulated spectra and, in certain cases, Fermi-arc loops (SFALs) as a collective reconstruction of topological surface states. We further show, both mathematically and through direct experimental consistency, that the LCM commensuration cycle is fully equivalent to the standard intercept-based surface-cell construction and the corresponding conventional surface-state calculations; this equivalence makes the underlying physical logic transparent and allows the ARPES data to serve as an experimental validation of the framework.

We thank the reviewer for the direct feedback regarding packaging and terminology. We acknowledge that in the previous round some of the clarifications expressed in our rebuttal were not sufficiently reflected in the manuscript text, which could leave an impression of over-packaging. In the present revision we therefore implemented substantive manuscript-level changes. We removed or substantially softened the highlighted phrases (e.g., "hidden moiré," "inherently mismatched," "translational commensurability is lost," and "bulk-surface incommensurability") and replaced them with more direct, physically transparent descriptions (phase-shifted multi-zone projections, LCM closure, replica modulation, and connectivity reconstruction). We also revised the title/abstract/conclusion to avoid overstating the scope of theory-experiment agreement and to ensure internal consistency: we now state explicitly that LCM-consistent calculations capture the reconstructed periodicity and dominant replica structure, while finer connectivity details can depend on microscopic boundary conditions beyond an idealized slab model.

The authors have acknowledged the equivalence of their LCM and the straightforward intercept-based method that is widely accepted in surface science and crystallography communities to generate the proper surface unit cell for low-symmetry or high Miller index surfaces. It is also well-known that the surface unit cells for high Miller index surfaces, like the NdAlSi(103) here, usually span serval primitive bulk unit cells in real space, thus, in reciprocal space, the surface Brillouin zones (SBZ) need a complete projection from 3D to 2D, not just a section of the first bulk BZ. The authors have also agreed that the key finding of the good agreement between the ARPES and surface Green's function calculation relies on the proper (103) surface unit cell and the inherent complete bulk-to-surface projection from 3D to 2D in the surface Green's function calculation.

We thank the reviewer for raising this point, but we would like to clarify the logical relation more precisely. We do not merely "acknowledge" the equivalence; rather, we **demonstrate** that the LCM commensuration criterion is **mathematically equivalent** to the straightforward intercept based construction for generating the proper surface unit cell. In this sense, the intercept based method can be viewed as a special-case implementation of the more general LCM criterion (i.e., one practical realization of the LCM commensuration condition for standard crystallographic settings). Moreover, our formulation provides a transparent mathematical explanation for why high-index surfaces often span multiple primitive bulk unit cells in real space, and why the surface Brillouin zone is properly defined only under a complete 3D to 2D bulk-to-surface projection, rather than by restricting attention to a section of the first bulk BZ. Importantly, in our Weyl-surface setting this is not merely a bookkeeping choice of a larger surface cell: the fractional projection shifts reorganize topological surface states because displaced Fermi arcs from different bulk zones overlap and hybridize, leading to reconstructed connectivity (including SFALs) with clear ARPES signatures.

Both Reviewer 2 and 3 pointed out that such proper surface unit cell and complete bulk-to-surface projection should be the default solution to consider for high Miller index surface.

We agree that using the proper surface unit cell and a complete bulk-to-surface mapping is the correct default approach; however, we would like to emphasize that a **complete 3D to 2D bulk-to-surface projection is not specific to high Miller-index surfaces**. Rather, it is a **general and, in principle, necessary prescription** for **any material and any surface** when formulating bulk-boundary correspondence and comparing to surface-sensitive spectroscopy. What makes many high-symmetry facets appear "special" in practice is that, for most common systems, the LCM commensuration condition yields N_{min}=1, so the complete projection reduces to the same result as a conventional first bulk Brillouin-zone projection picture; in these cases, restricting to the first bulk BZ is therefore effectively sufficient. In contrast, on low-symmetry facets (such as NdAlSi(103)), the complete projection generates fractionally shifted replicas that close only after N_{min}=3, making the full projection essential and its consequences experimentally visible. In a Weyl semimetal this has an additional topological consequence: when successive projections do not register, the displaced Fermi-arc manifolds can overlap and hybridize, reconstructing the boundary connectivity beyond what is encountered on high-symmetry facets.

The reduced SBZ compared to bulk BZ is not from the so-called "hidden moire" but the results of normal practice in surface science and crystallography.

We respectfully disagree with the implication that our earlier use of "hidden moiré" was meant to replace or contradict standard surface-science practice. The reduced/folded SBZ is indeed can obtain from the proper surface repeat unit, and its size is equivalent to that given by the straightforward intercept-based construction. However, the point of introducing "hidden moiré" was to provide a **mathematical and physically transparent interpretation** of how this reduced periodicity emerges in the projected electronic structure on a low-symmetry surface: projections of neighboring bulk Brillouin zones onto the surface generally differ by a non-integer lateral phase

shift, so their spectral weight overlaps and undergoes coherent hybridization in surface momentum space. The resulting moiré/interference pattern among multiple phase-shifted projections naturally produces the observed replica modulation and the emergent smaller repeating unit (the reduced SBZ). In this sense, "moiré" refers strictly to the coherent superposition of phase-shifted momentum-space projections, i.e., a purely mathematical description of the reconstructed periodicity, rather than to any specific moiré-physics scenario involving competing orders or twisted bilayers. In our Weyl-surface context, this mathematical mechanism has a direct physical consequence: phase-shifted projections displace Fermi arcs so that they can hybridize and reconstruct their connectivity, including the emergence of SFALs.

That said, we recognize that the term "hidden moiré" can be misunderstood, so in the revised manuscript we removed it and instead use the more conservative wording "moiré-like momentum-space modulation/replica structure," while keeping the same quantitative content and explicitly stating its equivalence to the intercept-based folded-SBZ construction.

The need of complete bulk-to-surface projection is also common in surface band structure calculations using surface Green's function. Again one such example is the CeX (X=P, As, Sb and Bi) surface states (please see Fig.4 in Kuroda et al, PRL 120, 086402 (2018)). Even for the high-symmetry rock-salt (001) surface, where the projection of two bulk BZ is needed for the band inversion at the X points to fully explain the gapped surface Dirac cones at the M point of the (001) SBZ. However, the authors have ignored such example in the rebuttal letter.

We feel the reviewer's summary does not fully capture the two-step logic underlying our use of the LCM criterion. First, we stress that one should consider the complete 3D to 2D projection in general. Importantly, for many common tetragonal (and other high-symmetry) situations, this complete projection is effectively equivalent to what one would infer from a conventional first-BZ projection picture, which is precisely why the "first-BZ-only" shortcut has become widespread in practice. We illustrate this equivalence (and its breakdown) explicitly in Fig. 3 and in the Supplementary Materials: for high-symmetry facets (e.g., (001) in NdAlSi), the projections from successive bulk BZs align in registry, so restricting to the first bulk BZ already reproduces the relevant surface periodicity. Second, this is the key point for low-symmetry facets, even when the full 3D to 2D projection is properly considered, projections of neighboring bulk BZs onto a low-symmetry surface generally acquire a non-integer lateral displacement (phase shift) relative to the surface reciprocal lattice. The LCM criterion is introduced specifically to determine when this fractional shift closes and thus fixes the minimal commensurate cycle (e.g., for tetragonal (103) we derive the 1/3 shift and the closure at N_{min}=3 in the Supplementary Materials). This closure directly determines the experimentally observed reduced periodicity/replica modulation and provides a practical prescription for commensurate modeling; for lower-symmetry lattice metrics, the situation can become more complex, and we explicitly note this in the Supplementary Materials Section 4. We emphasize that our main focus is not the general notion of including more than one bulk zone, but the resulting reconstruction of topological surface states on a Weyl boundary when phase-shifted projections cause Fermi arcs to overlap, hybridize, and reorganize their connectivity.

Regarding the CeX (X = P, As, Sb, Bi) example and related cases: we agree with the reviewer that such examples demonstrate that multi-zone projection can be essential even beyond the present material class, and we did not intend to dismiss them. Rather than "ignoring" these precedents, our point is that complete 3D to 2D projection should be treated as a necessary starting principle, not merely a "sometimes-used" option, many systems appear to be adequately described by a first-BZ picture only because the complete projection happens to reduce to the same result in those cases. The CeX example (and also well-known cases where more than one bulk BZ projection is needed to fully account for surface-state phenomenology) is therefore supportive of our central message: the correct projection framework is mandatory in general, and low-symmetry facets provide a particularly clear and experimentally accessible setting where its consequences fractional projection shifts, commensuration cycles, and the resulting reconstructed surface periodicity, become directly visible and must be handled systematically. To make this clearer to readers, we have revised the manuscript to acknowledge these precedents explicitly and to better position our contribution as an operational, ARPES-validated commensuration-guided route for modeling and interpreting reconstructed boundary spectra on low-symmetry Weyl surfaces.

If such "hidden moire" and similar overclaims are accepted for publication with just using the default practice to generate proper surface unit cell and complete bulk-to-surface projection, then should all high Miller surfaces of many other materials be called "hidden moire" from now on? Despite there is no competing interaction being introduced in the single phase of crystal structure for moire physics, but just BZ projection and folding.

We understand and share the reviewer's concern. Our intention is not to suggest that all high Miller-index surfaces should be labeled "hidden moiré." Here, "moiré" is used in a strictly mathematical sense: it denotes a moiré/interference-like modulation that can arise when projections of neighboring bulk BZs onto a surface are shifted by a non-integer phase (lateral displacement) and are then superposed, producing a new-period replica modulation whose closure is captured by the LCM criterion. This emergent reduced periodicity is fully equivalent to the folded SBZ obtained from the proper surface repeat unit in conventional surface crystallography; the "moiré" language simply makes the underlying phase-shift/superposition mechanism explicit rather than introducing new physics beyond projection and folding. Our emphasis is therefore not a label but a mechanism with measurable consequences: when phase-shifted multi-zone projections are coherent, they can reconstruct the topological boundary spectrum, including SFAL formation, which we directly resolve by ARPES on NdAlSi (103).

Importantly, we do not invoke the twisted-bilayer moiré-physics setting (competing real-space periodicities, correlated minibands, etc.). Instead, we present an operational mathematical framework for bulk-to-surface projection and commensuration that can be applied to any complex surface geometry, including high-index facets and more intricate boundaries such as zigzag-type surfaces or mixed local terminations, where the effective surface repeat unit between successive bulk-zone projections can be nontrivial. To avoid misunderstanding, we have rewritten the manuscript to remove "hidden moiré" phrasing and to use the more conservative term "moiré-like momentum-space modulation/replica structure," while explicitly stating its equivalence to standard intercept-based surface-cell construction and complete 3D to 2D bulk-to-surface projection.

Response to Reviewer's Comments

Reviewer #1 (Remarks to the Author):

I have reviewed the manuscript, the authors' rebuttal, and the revised Supplementary Information. In my view, the authors have made meaningful and constructive revisions. The manuscript is now substantially better aligned with the evidence than the prior version, and most of the concerns raised by all reviewers have been addressed at the manuscript level rather than only in the rebuttal.

The strengths of the manuscript remain significant. The work reports technically demanding ARPES measurements on the low-symmetry NdAlSi(103) surface, a surface that is not straightforward to prepare or interpret. The authors describe a careful pre-orientation, pre-cutting, and in situ cleavage procedure, and the ARPES measurements were performed with good energy/angular resolution at two synchrotron beamlines. The central experimental observation that flat, well-defined regions show sharp surface-state features while uneven/disordered regions suppress these states and reveal more bulk-like spectral weight is compelling and provides a useful experimental basis for separating surface, bulk-projected, and resonance-like contributions. The photon-energy-dependent measurements and comparison with DFT further support the interpretation that bulk projections shift systematically with k_perp, which is essential to the authors' LCM-based commensuration argument.

The most important improvement is the reframing of the theory-experiment comparison. The authors have changed the title to the more appropriate "Topological Fermi arc interference on Low-Symmetry Weyl Surfaces" and have rewritten the abstract to emphasize Fermi-arc interference, reconstructed periodicity, and dominant replica structure rather than the earlier, more expansive "hidden moiré topology" framing. They now explicitly distinguish the commensuration/periodicity problem, which is reasonably well captured by the LCM-guided calculations, from the detailed arc connectivity, including the loop-like features, which remain dependent on microscopic surface conditions not fully captured by the idealized slab model. This directly addresses the main concern from the previous review: the manuscript no longer implies that the calculations fully reproduce the most visually striking loop-like connectivity. The discussion now states more carefully that the calculations capture the reconstructed periodicity and dominant replica structure, while the detailed loop-like connectivity depends on termination mixing, reconstruction/disorder, and surface-bulk hybridization.

The authors also responded meaningfully to the concern that the LCM construction should not be advertised as new crystallographic mathematics. In the rebuttal, they explicitly state that the title, abstract, and conclusion were revised, that "hidden moiré" language was removed or softened, and that the LCM criterion is equivalent to standard intercept-based surface-cell construction. The Supplementary Information now gives a clear mathematical derivation of the LCM prescription and applies it to the NdAlSi(103) case, which justifies the three-cell surface repeat unit. It is reasonable to present the result not as a replacement for standard surface crystallography, but as an operational framework for making the projection problem explicit in

the context of ARPES and Weyl surface states.

There are still weaknesses. The evidence for closed surface Fermi-arc loops remains suggestive rather than definitive. The Supplementary Information presents ARPES cuts and Green's-function calculations supporting an assignment to topological surface Fermi arcs, but it also acknowledges that the detailed experimental connectivity is not fully captured by the idealized calculations. The proposed formation of SFALs through hybridization of arcs from different projected bulk zones is plausible, but it remains an interpretation rather than a quantitatively demonstrated microscopic mechanism. I am satisfied that the main text now frames this distinction appropriately, but I would encourage the authors to keep the language around "closed loops" and "generalized BBC" cautious, especially in the Supplementary Information.

There is also an apparent figure-label inconsistency in the Supplementary Information: it refers to the surface-state Fermi surface in Fig. 1e and the suppression of surface states in Fig. 1f, whereas the main text and Fig. 1 caption identify Fig. 1e as the uneven/disordered region and Fig. 1f as the flat, well-defined surface-state region. This should be fixed, but it does not affect the scientific conclusion.

Overall, I find that the authors have addressed the major review concerns in a meaningful way. The manuscript is still somewhat ambitious in its conceptual framing, and the SFAL interpretation is not as firmly established as the periodicity/commensuration result. However, the revised paper now makes that distinction clear. The ARPES data are strong, the low-symmetry surface problem is interesting, and the LCM-guided projection framework is useful when presented with the current level of caution. I therefore recommend the manuscript for publication in Nature Communications after minor textual corrections.

Response to Reviewer #1

We sincerely thank the reviewer for the careful and constructive assessment of our revised manuscript and for recommending its publication in *Nature Communications*. We appreciate the recognition of the clearer distinction between the commensuration/periodicity results and the interpretation of the loop-like features. We have also corrected the inconsistent assignments of Fig. 1e and Fig. 1f in the Supplementary Information in the revised version.

Reviewer #3 (Remarks to the Author):

The revised manuscript has been substantially improved by removing the overclaims and exaggerated statements, such as "hidden moire", "bulk and surface periodicities are inherently mismatched", "translational commensurability is lost" and "bulk-surface incommensurability". The rewriting of key parts of the manuscript (title, abstract and main text) with the more direct and physically transparent way has been well done and presents a much clear picture of the study to be appreciated by the community.

The detailed analysis in both the experimental ARPES and DFT calculations on the low-indexed NdAlSi (103) reveal the inherent need for a complete 3D to 2D projection (multiple bulk BZ to surface BZ) in order to better understand Weyl Fermi arcs and connectivity reconstruction. The LCM closure is a nice addition to the standard intercept-based surface-cell construction method.

With these substantial improvements, the manuscript is now suitable for publication in Nat. Comm.

Response to Reviewer #3

We sincerely thank the reviewer for the positive evaluation of our revised manuscript and for recommending it for publication in *Nature Communications*. We appreciate the recognition of the improved clarity of the presentation, the ARPES and DFT analyses, and the usefulness of the LCM closure framework.
